# Beyond KEAP1: The Context-Specific NRF2 Partner Code in Disease and Therapy

**DOI:** 10.3390/antiox15060759

**Published:** 2026-06-16

**Authors:** Seung-Jin Kwag, Jin-Kwon Lee, Seung-Jun Lee, Jeongyun Hwang, Young-Sool Hah

**Affiliations:** 1 Department of Surgery, Gyeongsang National University College of Medicine, 816-15 Jinju-daero, Jinju 52727, Republic of Korea; drksj77@gnu.ac.kr (S.-J.K.); 0789zxc@gnu.ac.kr (S.-J.L.); 2 Institute of Medical Science, Gyeongsang National University College of Medicine, 816-15 Jinju-daero, Jinju 52727, Republic of Korea; 3 Biomedical Research Institute, Gyeongsang National University Hospital, 79 Gangnam-ro, Jinju 52727, Republic of Korea; 4 Department of Surgery, Gyeongsang National University Changwon Hospital, 11 Samjungja-ro, Changwon 51472, Republic of Korea; gsbigcap@gmail.com; 5 Department of Convergence Medical Sciences, Gyeongsang National University, 816-15 Jinju-daero, Jinju 52727, Republic of Korea; dkdl10252@gnu.ac.kr

**Keywords:** NRF2, KEAP1, partner code, protein–protein interactions, BACH1, ferroptosis, cyclosporin A, transcriptional coactivators, drug repositioning, KEAP1-mutant cancer

## Abstract

Nuclear factor erythroid 2-related factor 2 (NRF2) has traditionally been framed as a Kelch-like ECH-associated protein 1 (KEAP1)-regulated stress-response transcription factor, but three observations now require a broader framework: NRF2 turnover is controlled by parallel E3 ligase systems; transcriptional output can be limited by coactivator assembly despite unchanged NRF2 abundance; and NRF2 activation can be beneficial or harmful depending on disease context, as illustrated by lung cancer models in which NRF2 paradoxically promotes metastasis through BTB and CNC homology 1 (BACH1) stabilization. We synthesize these observations into an NRF2 partner-code framework in which NRF2 acts as a context-dependent transcriptional platform assembled through four partly independent modules: a degradation module (KEAP1; β-transducin repeat-containing protein, β-TrCP; HMG-CoA reductase degradation protein 1/synoviolin 1, Hrd1/SYVN1; WD repeat-containing protein 23/DDB1- and CUL4-associated factor 11, WDR23/DCAF11); a cytoplasmic scaffold module (p62/sequestosome 1, p62/SQSTM1; IQ motif-containing GTPase-activating protein 1, IQGAP1; type I phosphatidylinositol 4-phosphate 5-kinase γ/heat shock protein 27, PIPKIγ–HSP27; peptidyl-prolyl cis-trans isomerase NIMA-interacting 1, PIN1; peptidyl-prolyl isomerase A/cyclophilin A, PPIA); a nuclear coactivator module at Neh4/5 (CREB-binding protein/p300, CBP/p300; receptor-associated coactivator 3/steroid receptor coactivator 3, RAC3/SRC-3; protein arginine methyltransferase 1/coactivator-associated arginine methyltransferase 1, PRMT1/CARM1; Mediator complex subunit 16, MED16); and a DNA/chromatin module at Neh1 (small musculoaponeurotic fibrosarcoma [Maf] proteins, BACH1, and chromodomain helicase DNA-binding protein 6, CHD6). Mapping 22 partners onto the Neh-domain architecture identifies approximately 25 pharmacologically addressable interfaces, stratified into four translational tiers. The framework reframes NRF2 pharmacology around one principle: the most actionable target is often a partner rather than NRF2 itself, with disease context dictating the direction of modulation. We close with five testable hypotheses and a partner-code decision matrix linking disease, biomarker, and candidate target.

## 1. Introduction

### 1.1. The Canonical NRF2 Model and Its Limits

For three decades, NRF2 biology has largely been interpreted through a two-component regulatory model. NRF2 was first identified by Moi and colleagues in 1994 [1] and was soon shown to heterodimerize with small Maf proteins through its cap’n’collar (CNC) basic-leucine zipper (bZIP) domain [2]. Functional studies in *Nfe2l2*-null mice then positioned NRF2 as a central regulator of cytoprotective transcription rather than as an erythroid-restricted factor [3]. In unstressed cells, KEAP1 acts as the cytoplasmic substrate adaptor that captures NRF2 and delivers it to the CUL3-RBX1 ubiquitin ligase complex for proteasomal degradation. The physiological importance of this brake is illustrated by the postnatal lethality of *Keap1*-null mice, which results from constitutive NRF2 activation [4]. Oxidative stress modifies reactive KEAP1 cysteines, particularly Cys151, Cys273, and Cys288, within a multi-residue sensor system that recognizes distinct electrophile classes [5,6,7,8]. This releases NRF2 from KEAP1-dependent turnover, allows NRF2 to accumulate in the nucleus, and activates the antioxidant response element (ARE) transcriptional program, with PKC-mediated phosphorylation of Ser40 contributing to nuclear translocation [9]. The resulting KEAP1-centric model has guided basic NRF2 biology and therapeutic development, including approved NRF2-pathway drugs such as dimethyl fumarate and omaveloxolone [10,11].

The model is correct, but it is no longer sufficient. Three observations, now supported by independent mechanistic studies, are difficult to explain with a binary KEAP1–NRF2 switch and motivate a broader regulatory description.

First, NRF2 stability is controlled by at least four E3 ligase systems. β-TrCP (SCF) ubiquitinates NRF2 at Neh6 in a redox-independent, GSK-3β-driven manner [12,13]. Hrd1/SYVN1 ubiquitinates NRF2 from the endoplasmic reticulum (ER) membrane in response to unfolded-protein-response signaling [14]. WDR23/DCAF11 (CRL4) ubiquitinates NRF2 in the nucleus and differs from KEAP1 in both localization and structural logic [15]. These pathways function in settings where KEAP1 remains intact, indicating that NRF2 stability integrates redox, growth-factor, proteostasis, and nuclear surveillance inputs.

Second, NRF2 transcriptional output is not determined solely by NRF2 abundance. Disruption of MED16, the Mediator tail subunit that bridges NRF2 to RNA polymerase II, attenuates the induction of approximately 75% of NRF2 target genes without reducing nuclear NRF2 levels [16]. Related constraints arise from small Maf availability [17], BACH1-mediated repression at shared AREs [18,19], and the SRC-3/RAC3–CBP/p300 coactivator complex at Neh4/5 [20,21]. Nuclear NRF2 abundance, therefore, is only one component of transcriptional competence.

Third, NRF2 activation is not uniformly protective. In *KEAP1*-mutant lung cancer, NRF2 activation can promote metastasis by stabilizing the transcriptional repressor BACH1 [22,23]. Antioxidants such as N-acetylcysteine and vitamin E, which can prevent primary tumorigenesis in some settings, accelerated metastasis through this axis in those models. Similarly, p62 accumulation can drive hepatocellular carcinogenesis through NRF2 activation in autophagy-deficient livers [24] while also protecting hepatocytes against ferroptosis [25]. These examples show that the disease consequences of NRF2 activation depend not only on the magnitude of activation but also on the partner subset engaged. The pan-cancer enrichment of KEAP1 and NFE2L2 alterations across The Cancer Genome Atlas further indicates that context-specific NRF2 rewiring is a recurrent oncogenic mechanism [26].

### 1.2. The NRF2 Partner Code

**Figure 1:**
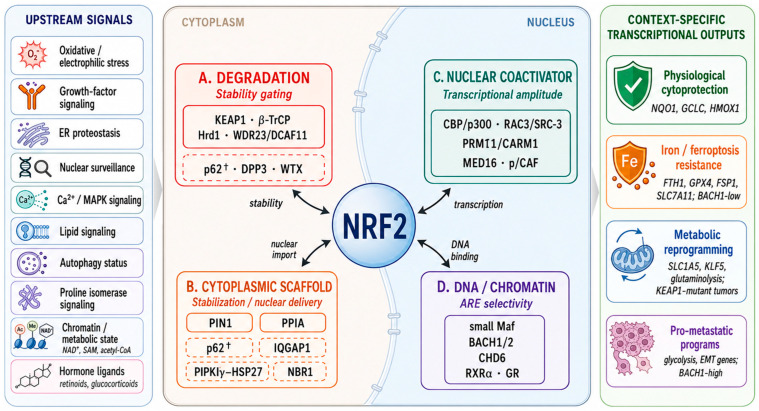
The NRF2 partner code: a four-module combinatorial framework. NRF2 is modeled as a context-dependent transcriptional platform whose output is set by partner assembly across four modules. (**A**) Degradation module: direct E3 substrate receptors (KEAP1, β-TrCP, Hrd1, WDR23/DCAF11) and indirect KEAP1 sequestrators (p62, DPP3, WTX) controlling NRF2 stability. (**B**) Cytoplasmic scaffold module (p62, IQGAP1, PIPKIγ–HSP27, PIN1, PPIA, NBR1) linking non-redox signals to stabilization and nuclear delivery. (**C**) Nuclear coactivator module (CBP/p300, RAC3/SRC-3, PRMT1/CARM1, MED16, p/CAF) controlling transcriptional amplitude at Neh4/5. (**D**) DNA/chromatin module (small Maf, BACH1/2, CHD6, RXRα, GR) shaping ARE selectivity. p62 acts in more than one module. Flanking columns map upstream signals (**left**) to context-specific transcriptional outputs (**right**). Solid outlines indicate direct NRF2-binding partners, whereas dashed outlines indicate indirect regulators that modulate NRF2 through an intermediary, such as KEAP1 sequestration, MEK–ERK scaffolding, or HSP27-dependent stabilization. p62^†^ is a bridging partner that functions in more than one module, acting both as a KEAP1-sequestering degradation modulator and as a cytoplasmic scaffold. Representative partners are shown; detailed evidence strength for each interaction is provided in the partner tables of Section 2, Section 3 and Section 4 and Supplementary Table S2 using the Evidence tier system.

These observations are not isolated exceptions. Together, they support a model in which NRF2 functions less as a single-switch master regulator and more as a context-dependent transcriptional platform. Its output is shaped by the combinatorial assembly of partners at four modules (Figure 1): degradation partners that gate stability; cytoplasmic scaffolds that translate non-redox signals; nuclear coactivators that determine transcriptional amplitude and gene selectivity; and DNA/chromatin partners that specify which ARE-containing genes are activated or repressed. We refer to this regulatory architecture as the NRF2 partner code. Key concepts of this framework are defined concisely in Box 1 (a fuller glossary of partner-code terminology is provided in Supplementary Materials, S1).

Box 1 **Key concepts of the partner-code framework.**

Partner code. The combinatorial assembly of NRF2-interacting partners that, collectively, determines the stability, localization, transcriptional amplitude, and target-gene selectivity of NRF2 in each cellular context.

Partner-code framework. The four-module conceptual organization introduced in this review (degradation, cytoplasmic scaffold, nuclear coactivator, and DNA/chromatin modules) is used to read the partner code.

Module. A group of partners defined by the upstream signal they convert into an NRF2-relevant output and by the biochemical step they control (stability, scaffolding/trafficking, transcriptional amplitude, or ARE selectivity).

Bridge partner. a partner that acts in more than one module (e.g., p62, which both sequesters KEAP1 and scaffolds NRF2; PIN1, which scaffolds NRF2 and isomerizes the Neh6 phospho-degron). Bridge partners are assigned to a primary module, with cross-module activity explicitly noted.

Partner switching. a change in which partner is dominant—or in the direction of its effect—between cellular or disease contexts, such that the same partner can favor NRF2 stabilization in one setting and degradation or repression in another.

Rate-limiting partner. The partner whose abundance or activity sets the level of NRF2 output in a particular disease context; the most informative therapeutic target is often the rate-limiting partner rather than NRF2 itself.

Evidence tier. The graded strength of evidence for a partner interaction: Tier I (structural and genetic), Tier II (mechanistic with orthogonal validation), or Tier III (emerging or inferred). Used throughout the tables and figures.

Therapeutic tier. The translational maturity of a partner-selective intervention, from approved agents engaging a partner interface (Tier 1) through repositioning candidates (Tier 2) to partner-adjacent or hypothesis-stage agents (Tier 3, sub-stratified 3a/3b/3c).

The four-module organization is intended as a conceptual advance over previous partner catalogs in two specific senses. First, it is signal-input-organized rather than localization-organized: each module is defined by the upstream signal that the partner converts into an NRF2-relevant output (redox, growth-factor, ER proteostasis, and nuclear surveillance for the degradation module; Ca^2+^/MAPK, lipid, autophagy, and proline isomerase signals for the cytoplasmic scaffold; coactivator amplitude at Neh4/5; ARE selectivity and repression at Neh1), rather than by where the partner happens to be found. Second, the framework is predictive at the level of disease phenotype: it implies that disrupting different modules in the same disease should produce qualitatively different outputs (for example, KEAP1 loss versus β-TrCP loss versus MED16 loss should not be expected to phenocopy one another), and that the same partner can require activation in one disease and inhibition in another when the rate-limiting module differs. To our knowledge, existing categorizations of NRF2 interactors do not explicitly make either claim.

Several clarifications follow from this organization. The four modules were selected because they correspond to the four operationally distinct biochemical steps that determine NRF2 output (stability, scaffolding/trafficking, transcriptional amplitude, and ARE selectivity), each of which can be perturbed in disease and addressed pharmacologically. They are not intended to be mutually exclusive: partners that act at more than one step—most notably p62 (KEAP1 sequestration plus cytoplasmic scaffolding) and PIN1 (cytoplasmic scaffolding plus phospho-degron isomerization at Neh6)—are bridge partners by design. We adopt the convention of assigning a bridge partner to its primary functional context and explicitly noting cross-module activity (Box 2, criteria 3 and 4; Supplementary Table S3 records dual-module partners). Module membership, therefore, follows the dominant biochemistry of the partner rather than the exclusivity principle.

Box 2 **Defining the NRF2 partner code: criteria for module assignment.**

The framework organizes regulatory partners into four functional modules, with assignment based on operational criteria applied in priority order. Bridging partners—those that satisfy criteria for two modules—are listed in their primary module and explicitly cross-referenced.

Degradation module. A partner controls NRF2 protein turnover via direct or indirect ubiquitination, and its loss measurably prolongs NRF2 half-life. This module includes E3 ligase substrate adaptors that bind NRF2 directly (KEAP1 at Neh2; β-TrCP at Neh6; Hrd1/SYVN1 at the C-terminal region; WDR23/DCAF11 at the DIDLID motif within Neh2) and indirect modulators that operate at the same physical hub by sequestering KEAP1 (p62, NBR1, DPP3, WTX/AMER1, PALB2, p21/CDKN1A).

Cytoplasmic scaffold module. A partner binds NRF2 in the cytoplasm without primarily targeting it for degradation, instead modulating its conformation, lipid environment, chaperone association, kinase coupling, or nuclear import rate. Members include condensate-forming receptors (p62 in its non-degradative role), kinase scaffolds (IQGAP1), lipid-signaling and chaperone scaffolds (PIPKIγ–HSP27), conformational regulators (PIN1), and structural KEAP1-blockers that bind NRF2 directly (PPIA).

Nuclear coactivator module. A partner binds NRF2 in the nucleus, primarily at Neh4/5 or the Neh1 bZIP region, and enhances or modulates RNA Pol II recruitment and transcriptional amplitude. Members include acetyltransferases (CBP, p300, p/CAF/KAT2B), p160 coactivators (RAC3/SRC-3), arginine methyltransferases (PRMT1, CARM1), and Mediator subunits (MED16, MED23, MED24).

DNA/chromatin module. A partner binds DNA together with NRF2 at the bZIP heterodimer interface (small Maf proteins), competes with NRF2 for DNA binding (BACH1, BACH2), remodels chromatin at NRF2-bound enhancers (CHD6), or modulates NRF2 transcriptional output through nuclear receptor crosstalk (RXRα, GR).

Bridging cases. p62 is listed under the degradation module because its dominant cellular function is KEAP1 sequestration, but its condensate-forming activity is a cytoplasmic scaffold function. PPIA is listed in the scaffold module because its primary structural feature is direct NRF2 binding, but its functional consequence is the indirect occlusion of KEAP1. Both are explicitly noted in the relevant sections.

The framework makes three claims that are developed across the review. First, the modules are functionally distinguishable: each integrates a different upstream signal, including redox stress, growth-factor/AKT signaling, ER proteostasis, lipid/Ca^2+^/MAPK signaling, endocrine inputs, and chromatin state. Second, the modules can be mapped onto the seven Neh domains of NRF2 and the disordered linkers between them, providing a coordinate system for partners, post-translational modifications, and disease-associated mutations. Third, the same map is pharmacologically informative because each module exposes interfaces that may be modulated independently.

### 1.3. What This Review Delivers

This review develops the framework in five steps. Section 2 describes the multi-E3 degradation module, treating KEAP1 as one arm of a broader turnover network. Section 3 defines the cytoplasmic scaffold module, with emphasis on recently characterized partners such as PPIA, PIPKIγ–HSP27, IQGAP1, and PIN1. Section 4 maps the nuclear transcriptional module at Neh4/5 and Neh1. Section 5 consolidates these layers into a Neh-domain partner atlas. Section 6 and Section 7 then read disease biology and therapeutic pharmacology through the partner-code lens, distinguishing evidence-supported interventions from hypothesis-generating opportunities. Section 8 converts the framework into five experimentally testable hypotheses.

This review differs in scope and orientation from several recent syntheses of NRF2 partner biology. Nam and Keum [27] comprehensively reviewed direct NRF2 binding partners and organized them around Neh-domain interactions and their effects on NRF2 stability or activity, but did not impose a four-module partly independent module framework. Poh and colleagues [28] generated a high-density NRF2 interactome by DULIP/FCCS and identified 46 new partners with quantitative binding affinities, but did not systematically project all partners onto a comprehensive Neh-domain coordinate system or map them to therapeutic interfaces. The most recent 30-year synthesis [29] traces the chronological arc of NRF2 drug discovery but retains a KEAP1-centric pharmacological organization. The present review contributes three elements that none of these provides: a four-module partly independent module framework with explicit assignment criteria, a Neh-domain coordinate atlas onto which 22 partners, recurrent disease mutations, and post-translational modifications are simultaneously projected (Section 5), and a four-tier therapeutic-maturity stratification (Tier 1, approved NRF2-pathway therapeutics; Tier 2, approved drugs with partner-code repositioning potential; Tier 3, clinical-stage partner-adjacent agents; Tier 4, preclinical or conceptual interfaces) that keeps biological attractiveness and clinical readiness explicitly separate (Section 7).

### 1.4. Scope and Evidence-Grading Approach

This article is a mechanistic narrative review with structured evidence grading rather than a systematic review. We prioritize primary studies that identify direct NRF2 partners, map binding surfaces or post-translational modifications, test functional consequences by genetic or pharmacological perturbation, or connect partner engagement to disease models or clinical-stage therapeutics. Throughout the review, we distinguish four evidence levels: direct biochemical/structural evidence, cellular mechanistic evidence, disease-model evidence, and clinical or translational evidence. This distinction separates mature therapeutic opportunities from biologically attractive but still speculative interfaces.

To clarify how the present review differs from existing syntheses, Table 1 contrasts it with five representative recent NRF2 reviews along four axes: primary scope, interactome coverage, therapeutic emphasis, and conceptual frame. Previous reviews have been organized either around the canonical KEAP1–NRF2 axis, downstream transcriptional output, compound catalogs, or systems-level disease networks. None, to our knowledge, integrates all four regulatory modules with a residue-level Neh-domain coordinate system and a biomarker-stratified therapeutic decision matrix, which is the specific contribution of the present work.

**Table 1:** Positioning of this review against representative recent NRF2 reviews.

Review	Primary Scope	Interactome Coverage	Therapeutic Emphasis	Conceptual Frame	Ref.
Yamamoto, Kensler & Motohashi	Molecular mechanism of KEAP1–NRF2 redox sensing	KEAP1-centric; cysteine sensor detail	KEAP1 cysteine modifiers; aging	Canonical single-axis	[30]
Tonelli, Chio & Tuveson	Transcriptional regulation and target-gene programs	DNA-binding and coactivator emphasis	NRF2 in cancer metabolism	Downstream-output focused	[31]
Cuadrado et al.	Systems medicine view of NRF2 in chronic disease	KEAP1 plus selected signaling inputs	NRF2 activators; diseasome map	Network/systems	[10]
Cuadrado et al.	Drug discovery against the KEAP1–NRF2 partnership	KEAP1–NRF2 interface; some E3 context	KEAP1–NRF2 PPI inhibitors; activators	KEAP1-partnership centric	[11]
Robledinos-Antón et al.	Catalog of NRF2 activators and inhibitors	Limited (compound-indexed, not partner-indexed)	Clinical-stage activators and inhibitors	Compound-centric	[32]
This review (2026; Antioxidants)	Integrated four-module partner code across stability, scaffolding, coactivation, and ARE selectivity	All four modules; 22 partners mapped to Neh domains	Partner-selective pharmacology; biomarker-stratified decision matrix	Signal-input-organized, structurally anchored, predictive	

Clinical translation follows three principles: the optimal target is often not NRF2 itself; several approved drugs intersect the partner code mechanistically and may warrant biomarker-stratified repositioning studies; and the same partner may require activation in one disease but inhibition in another. These principles shift NRF2 pharmacology away from broad pathway activation toward partner-selective modulation.

In reading this framework, it is also useful to distinguish partners that bind NRF2 directly from those that act indirectly through an upstream step. Direct interactors define a discrete molecular interface and are, in principle, addressable at that interface, whereas indirect regulators (for example, KEAP1 sequestrators such as p62, or kinase- and chaperone-dependent inputs such as IQGAP1 and PIPKIγ–HSP27) act one step removed from NRF2 and are often more naturally approached through the upstream pathway. We treat both categories within a single framework but explicitly label them—in the partner tables (the “Type” annotation) and in the module-assignment criteria (Box 2)—so that their integration does not obscure this mechanistic difference. We deliberately refrain from ranking the two categories by druggability, since tractability depends on interface geometry, achievable selectivity, and on-target liabilities that remain unresolved for several of these nodes.

Literature selection. Primary literature was retrieved from PubMed, Web of Science, and Scopus from January 1994 (the year NFE2L2 was cloned) through January 2026 using combinations of the terms “NRF2,” “NFE2L2,” “KEAP1,” “binding partner,” “interactome,” “Neh domain,” “BACH1,” “MED16,” “β-TrCP,” “WDR23,” “Hrd1/SYVN1,” “PPIA,” “PIN1,” “IQGAP1,” “HSP27,” “p62/SQSTM1,” “ferroptosis,” “*KEAP1*-mutant lung cancer,” “omaveloxolone,” and “dimethyl fumarate.” Inclusion criteria prioritized primary studies identifying direct NRF2 partners, mapping binding surfaces or post-translational modifications, testing functional consequences by genetic or pharmacological perturbation, or connecting partner engagement to disease models or clinical-stage therapeutics. Foundational papers were retained even when older; conference abstracts, non-peer-reviewed preprints, and non-English publications without translated abstracts were excluded. This article is a structured narrative review and does not claim PRISMA-level systematic completeness.

## 2. The Multi-E3 Degradation Code: Parallel Routes to NRF2 Turnover

The canonical model—KEAP1 captures NRF2, oxidative stress releases it—is accurate but incomplete. At least three additional E3 ligase systems ubiquitinate NRF2 in parallel with the CRL3^KEAP1 axis. Each engages a distinct NRF2 region and responds to a different cellular cue [11] (Table 2). Together, KEAP1 (CRL3), β-TrCP (SCF), Hrd1/SYVN1, and WDR23/DCAF11 (CRL4) create a multi-input degradation code in which redox stress, growth-factor signaling, ER proteostasis, and nuclear residence can independently influence NRF2 half-life.

**Table 2:** NRF2 degradation partners: the multi-E3 code.

Partner	Type	Binding Region on NRF2	Recognition Signal	Dominant Cellular Context	Disease Relevance	Evidence Level (Tier)	Refs.
KEAP1	Cul3-RING E3 substrate adaptor	Neh2 (DLG + ETGE motifs)	Basal redox; Cys151/273/288 sensing	Basal turnover in all cells; cytoplasm	NSCLC, HNSCC, HCC mutations (ETGE/DLG)	Tier I; Structural + Mutagenesis	[2,33,34,35]
β-TrCP/SCF	F-box E3 substrate receptor	Neh6 (DSGIS + DSAPGS)	GSK-3β-dependent phosphorylation of S335/338/342/347	Insulin/PI3K-AKT off; redox-independent	T2D, Alzheimer’s, Parkinson’s	Tier I; Mutagenesis + Functional KO	[11,12,13]
Hrd1/SYVN1	ER-resident RING E3	C-terminal region (Neh3 vicinity)	ER stress/IRE1α-XBP1 axis	ER stress; chronic liver disease	Cirrhosis, hepatic fibrosis, renal IRI	Tier II; Co-IP + Functional KO	[14,36]
WDR23/DCAF11	DDB1-CUL4 substrate receptor	NEH2 (DIDLID MOTIF)	Basal nuclear surveillance	Nuclear NRF2 turnover; KEAP1-independent	Cancer, aging	Tier II; Mutagenesis + Functional	[15,37,38,39]
p62/SQSTM1	Indirect—sequesters KEAP1	(KIR motif binds KEAP1 Kelch)	Autophagy stress; TBK1 phospho-S349	Autophagy-deficient HCC; Mallory-Denk bodies	HCC, NASH, neurodegeneration	Tier II; Structural (KIR-Kelch) + Functional	[24,40,41,42,43,44]
DPP3, WTX, PALB2, p21	Indirect—sequester KEAP1	(ETGE/DLG-like motifs)	Stress, Wnt, DDR, cell cycle	Stress contexts; *KEAP1*-mutant tumors	Cancer, DDR-driven NRF2 stabilization	Tier III; Structural (DPP3-Kelch) + cellular	[45,46,47,48,49,50]

Evidence tiers (column “Evidence level”): Tier I, supported by crystal/cryo-EM structure, site-directed mutagenesis at the interface, and orthogonal genetic loss-of-function evidence (KEAP1, β-TrCP); Tier II, supported by co-immunoprecipitation, pulldown, or interactome data with at least one orthogonal functional assay (Hrd1/SYVN1, WDR23/DCAF11); Tier III, single-laboratory or inferred (selected sequestrators). Direct substrate receptors bind NRF2 directly, whereas KEAP1 sequestrators (p62, DPP3, WTX) act indirectly by competing for KEAP1 rather than by binding NRF2. Full evidence annotations are provided in Supplementary Table S2.

### 2.1. The Canonical KEAP1 Axis: Redox-Sensing Dimer That Ubiquitinates a Single Substrate

KEAP1, the substrate adaptor for the CUL3-RBX1 E3 ligase complex, has dominated the NRF2 literature since 1999 [2,33]. Two KEAP1 monomers form a dimer through their BTB domains and engage one NRF2 molecule through simultaneous binding of two degron motifs in Neh2: the high-affinity ETGE (residues 77–82) and the low-affinity DLG (residues 23–31), each occupying one Kelch-domain pocket [34,35]. Crystal structures of the Kelch domain bound to ETGE and DLG peptides, solved independently by the Yamamoto and Hannink groups [51,52], established the molecular basis for this asymmetric two-site recognition. This hinge-and-latch model positions seven lysines between DLG and ETGE for CUL3-RBX1-mediated polyubiquitination, driving NRF2 to the proteasome with a basal half-life of 15–30 min. Quantitative measurements of intracellular complex stoichiometry confirm that KEAP1, NRF2, and CUL3 exist in fixed ratios that constrain regulatory dynamics [53].

**Figure 2:**
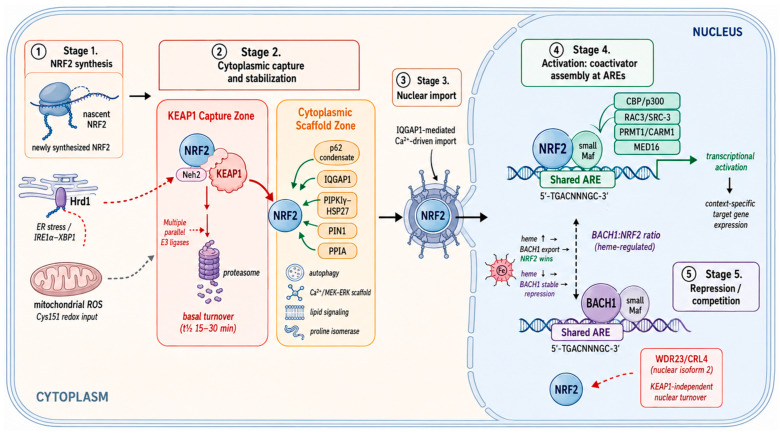
Cellular spatial framework of the NRF2 partner code. NRF2 is followed across five stages. Stage 1: cytoplasmic synthesis. Stage 2: KEAP1 capture at Neh2 and proteasomal turnover, with cytoplasmic scaffold partners (p62, IQGAP1, PIPKIγ–HSP27, PIN1, PPIA) providing alternative stabilization, ER-membrane Hrd1 adding a proteostasis-sensitive route, and mitochondrial ROS feeding KEAP1. Stage 3: partner-assisted nuclear import. Stage 4: assembly with small Maf, coactivators, and chromatin partners to activate or repress ARE genes. Stage 5: BACH1:small-Maf competes with NRF2:small-Maf at shared AREs (heme-set ratio), while nuclear WDR23/DCAF11–CRL4 drives KEAP1-independent turnover. Spatial separation among cytoplasmic, ER-associated, and nuclear control points is emphasized.

Redox sensing is mediated by reactive cysteines in the KEAP1 IVR and BTB domains. Cys151, Cys273, and Cys288 are major sensors for distinct electrophiles [54,55], forming a multi-residue “cysteine code” that has been tested through transgenic complementation studies in mice [56]. Saito and colleagues [7] classified NRF2 inducers into four cysteine-sensor classes: Cys151-preferring agents (sulforaphane and tert-butylhydroquinone), Cys288-preferring agents (15-deoxy-Δ12,14-prostaglandin J2), Cys151/Cys273/Cys288 cooperative agents (4-hydroxynonenal), and Cys151-independent/Cys273-Cys288-dependent electrophiles (nitro-oleic acid). This logic explains why KEAP1 is best understood not as a single redox switch but as a sensor array (the cellular spatial framework integrating these inputs is illustrated in Figure 2).

The clinical importance of this hub is reflected in two distinct mutational patterns. *KEAP1* loss-of-function mutations were first systematically cataloged in non-small-cell lung cancer (NSCLC) by Singh and colleagues [57], who established the initial clinicopathological association. Subsequent studies refined the prevalence to 12–20% of NSCLC adenocarcinomas, with additional frequency in head and neck squamous cell carcinoma (HNSCC) and esophageal squamous carcinomas [58,59,60,61,62]; loss of KEAP1 function provides cancer-relevant growth advantages in lung adenocarcinoma cells [63], and the KEAP1/NFE2L2 axis is consistently identified among the ten canonical oncogenic signaling pathways across The Cancer Genome Atlas (TCGA) pan-cancer atlas [26,64]. *NFE2L2* gain-of-function mutations, in contrast, are almost exclusively confined to the DLG and ETGE motifs of Neh2 [65,66], with R34, D29, W24, L30, D77, E79, T80, and E82 emerging as recurrent residues—precisely the residues that crystal structures predict to be critical for KEAP1 Kelch-pocket engagement [51,52]. Both mutation classes converge on the same outcome—uncoupling of NRF2 from KEAP1-mediated turnover—but importantly, they leave the β-TrCP, Hrd1, and WDR23 axes intact. As Section 7 argues, this asymmetry makes the non-KEAP1 axes attractive synthetic-lethal targets in *KEAP1*/*NFE2L2*-mutant tumors [67,68].

### 2.2. The β-TrCP/SCF Axis: Redox-Independent, AKT-Tunable Degron at Neh6

β-TrCP (encoded by *FBXW1A* and *FBXW11*) recognizes a GSK-3β-primed phosphodegron in the Neh6 domain comprising two motifs—DSGIS (centered on S335/338/342) and DSAPGS (around S347)—phosphorylated in tandem by GSK-3β [12,13]. β-TrCP–NRF2 binding triggers SCF-driven ubiquitination entirely independently of redox status: oxidative stress that fully inactivates KEAP1 has no effect on β-TrCP-mediated turnover.

The physiological logic lies upstream of GSK-3β. PI3K–AKT signaling phosphorylates and inhibits GSK-3β, which de-primes the Neh6 phosphodegron and stabilizes NRF2—a redox-independent regulatory axis comprehensively characterized in a systems medicine review by Cuadrado and colleagues [69]. The β-TrCP axis, therefore, couples NRF2 protein levels to growth-factor and insulin signaling. This redox-independent dimension has emerged as central in three disease contexts. In type 2 diabetes, chronic activation of the PI3K/AKT pathway inhibits GSK-3, reducing β-TrCP-mediated NRF2 turnover by Neh6 even when KEAP1 is functional [70,71], and the resulting paradoxical NRF2 stabilization contributes to pancreatic β-cell dysfunction by blunting glucose-stimulated ROS signaling [70,71]. In neurodegeneration, GSK-3β hyperactivity in Alzheimer’s and Parkinson’s models destabilizes NRF2 [72,73,74]. In oncogenic RAS-driven tumors, hyperactive AKT elevates NRF2 in a Neh6-dependent manner, independent of KEAP1 or NFE2L2 mutations [75], establishing β-TrCP as a mechanism for KEAP1-independent oncogenic NRF2 activation; the same mechanism participates in NRF2-driven metabolic reprogramming that fuels tumor growth [76].

Therapeutically, the β-TrCP axis is actionable because several GSK-3β inhibitors already have clinical experience, including lithium for bipolar disorder and tideglusib in neurodegenerative or neuromuscular indications. These agents can stabilize NRF2 by de-priming the Neh6 phosphodegron. Their repositioning for diseases of redox dyshomeostasis remains a translational hypothesis that requires disease-specific pharmacodynamic and safety validation.

### 2.3. The Hrd1/SYVN1 Axis: Er-Proteostasis-Gated Brake

Hrd1 (synoviolin/*SYVN1*) is a multi-pass ER-membrane RING-type E3 ligase classically associated with ER-associated degradation. Wu and colleagues [14] showed that Hrd1 ubiquitinates NRF2 directly through binding to the C-terminal half of NRF2 (around the Neh3 region). The functional significance was striking: in cirrhotic liver, the IRE1α–XBP1 arm of the unfolded-protein response transcriptionally upregulates Hrd1, which then catalyzes NRF2 ubiquitination—explaining the paradoxical loss of NRF2 protein in the very tissue most flooded with reactive oxygen species.

The Hrd1 axis is supported in renal ischemia–reperfusion injury [36], chronic alcoholic liver disease, and cardiac ER-stress models. Its conceptual contribution is that NRF2 stability is gated by proteostasis state, not only by redox state. Hrd1 inhibitors or IRE1α–XBP1 pathway blockers may preserve NRF2 in cirrhotic and post-ischemic contexts in which KEAP1 inhibition alone would be insufficient [14].

### 2.4. The WDR23/DCAF11–CRL4 Axis: Nuclear Surveillance Distinct from KEAP1

WDR23/DCAF11 is a DDB1-binding substrate adaptor of the CUL4 E3 ligase complex (CRL4), evolutionarily conserved from *C. elegans*—where its ortholog WDR-23 was originally characterized as a regulator of oxidative stress resistance [77]. Lo et al. [15] demonstrated that human WDR23 ubiquitinates NRF2 in a manner structurally distinct from KEAP1: WDR23 binds the DIDLID motif within the Neh2 domain, an interface distinct from the DLG/ETGE motifs used by KEAP1 and operates even in KEAP1-null A549 lung cancer cells. WDR23 exists as two isoforms with distinct subcellular localizations: cytoplasmic isoform 1 and nuclear-enriched isoform 2 [15]. Spatola and colleagues subsequently established the functional asymmetry of these isoforms toward distinct substrates, including SKN-1 and GEN-1 in *C. elegans* [39]. The nuclear isoform mediates a substantial fraction of NRF2 turnover specifically within the nucleus, providing a regulatory layer that operates after KEAP1 has released NRF2 and after nuclear translocation.

The partner-code implication is that cytoplasmic and nuclear compartments run distinct turnover programs. KEAP1 acts primarily in the cytoplasm, whereas WDR23 functions predominantly in the nucleus. This spatial separation may shape the timing and duration of NRF2 transcriptional pulses. WDR23 also modulates drug-metabolizing enzyme expression [38] and mitochondrial homeostasis [37], positioning the WDR23–NRF2 interface as a candidate nuclear control point for future therapeutic development.

Evidence strength and open questions (WDR23). The WDR23/DCAF11–CRL4 axis is supported by direct biochemical demonstration in mammalian cells [15] and by independent functional confirmation in *C. elegans* *wdr*-23 studies [37]. Disease-stage human evidence and pharmacodynamic biomarkers, however, remain limited. No chemical lead disrupts the WDR23–NRF2 interface, leaving the axis at the conceptual stage for therapeutic exploitation. Whether WDR23 surveillance dominates over KEAP1 turnover in any specific disease context is an open question.

### 2.5. KEAP1 Sequestration Partners: Indirect Modulators at the Same Hub

A fifth class of degradation-modulating partners does not bind NRF2 directly but instead sequesters KEAP1 itself, occupying the Kelch pocket that would otherwise capture the NRF2 ETGE. The archetypal example is p62/SQSTM1, whose KIR motif (DPSTGE) mimics the NRF2 ETGE [40,41,42]. When p62 oligomerizes through its PB1 domain, it forms liquid–liquid phase-separated condensates—a behavior independently demonstrated by three groups using complementary approaches: Sun et al. showed that polyubiquitin chains drive p62 phase separation [43]; Zaffagnini et al. reconstituted the process biochemically and revealed that p62 filaments capture and present ubiquitinated cargos for autophagy [78]; and Kageyama et al. visualized p62 droplets as platforms simultaneously serving autophagosome formation and antioxidative stress response [79]. Yang et al. further showed that cytoplasmic DAXX drives p62 phase condensation as an NRF2-activating signal [80], extending the mechanism to non-autophagic stress contexts. This convergent evidence—four independent labs, complementary techniques—establishes that p62 condensates physically immobilize KEAP1 within autophagy-targeted droplets, a non-redox, condensate-driven NRF2 stabilization that operates whenever autophagy flux is impaired. The mechanism drives constitutive NRF2 activation in autophagy-deficient hepatocellular carcinoma [24,44], in alcoholic steatohepatitis, and in pathological p62 inclusions seen in liver disease and neurodegeneration. NBR1 can substitute for p62 in some contexts [81].

Additional KEAP1 sequestration partners include DPP3 [48,49], WTX/AMER1 [45], PALB2 [47,50], and p21/CDKN1A [46], each carrying ETGE- or DLG-like motifs that compete with NRF2 for KEAP1 binding. They operate in different cellular contexts: DPP3 in stress and *KEAP1*-mutant tumors; WTX bridging Wnt and NRF2 signaling; PALB2 linking the BRCA-mediated DNA-damage response to NRF2 stabilization; p21 connecting p53-driven cell-cycle arrest. Because these partners operate at the same physical hub (the KEAP1 Kelch pocket), they can be conceptualized as endogenous ETGE-mimetic peptides—a natural pharmacology that has inspired the design of synthetic KEAP1–NRF2 PPI inhibitors (Section 7).

### 2.6. Reading the Multi-E3 Code

The four E3 axes create a spatial and signaling logic on NRF2. Neh2 is the redox-sensing and KEAP1-competition zone. Neh6 is the metabolic-state zone, controlled by GSK-3β–β-TrCP and PI3K–AKT signaling. The C-terminal region near Neh3 and adjacent surfaces contribute to proteostasis and nuclear surveillance control through Hrd1 and WDR23. These zones are partially orthogonal, allowing one NRF2 molecule to be stabilized through one axis while remaining susceptible to another.

Three implications follow. First, disease specificity can often be read from the perturbed E3 axis: Hrd1 induction in cirrhosis, β-TrCP activation in diabetes and neurodegeneration, KEAP1 inactivation in cancer, and p62-driven KEAP1 sequestration in autophagy-deficient hepatocellular carcinoma (HCC). Second, spatial segregation creates opportunities for partner-selective therapy rather than uniform NRF2 activation. Third, *KEAP1*-mutant tumors are not NRF2-maximal states; they remain dependent on β-TrCP, WDR23, and nuclear coactivator partners.

## 3. The Cytoplasmic Scaffold Module: NRF2 Stability as Signaling Integration

In the classical model, cytoplasmic NRF2 is a short-lived intermediate captured by KEAP1, ubiquitinated, and degraded. The scaffold module expands this view. Cytoplasmic NRF2 can be stabilized by partners that are not E3 ligases and do not simply mimic oxidative stress. Instead, these partners link calcium flux, MAPK signaling, lipid signaling, autophagy, chaperone state, and proline isomerization to NRF2 half-life and nuclear delivery.

The five partners discussed below—p62/SQSTM1, IQGAP1, PIPKIγ–HSP27, PIN1, and PPIA—illustrate distinct signal-translation logics that converge on a common output: non-degradative stabilization of NRF2. Some are druggable with existing compounds, whereas others remain mechanistically attractive but pharmacologically immature. Together with the nuclear coactivator partners discussed in Section 4, these scaffold and coactivator partners are summarized in Table 3.

**Table 3:** Cytoplasmic scaffolds and nuclear coactivators of NRF2: the functional module map.

Partner	Module	Binding Region on NRF2	Functional Output	Signal Integrated	Evidence Level	Refs.
p62/SQSTM1	Cytoplasmic scaffold (also degradation hub)	KEAP1 KIR motif (DPSTGE)—sequesters KEAP1	KEAP1 sequestration; NRF2 stabilization; condensate formation; positive feedback loop	Autophagy flux, oxidative stress, TBK1-S349/351	Structural + functional	[24,40,41,42,43]
NBR1	Cytoplasmic scaffold	Indirect via p62 condensates	Co-condensate formation; selective autophagy	Autophagy receptor crosstalk	Co-IP/inferred	[81]
IQGAP1	Cytoplasmic scaffold + MAPK relay	IQ domain (aa 699–905) ↔ NRF2 (Neh1/bZIP region)	NRF2 stabilization; Ca^2+^-driven nuclear import; MEK-ERK scaffolding	Ca^2+^/calmodulin, MEK-ERK	Co-IP + functional	[82,83,84]
PIPKIγ/HSP27	Cytoplasmic scaffold + lipid signaling	Direct PIPKIγ–NRF2; HSP27 stabilization complex	Lipid-dependent NRF2 stabilization; chemoresistance	PI(4,5)P_2_ pool, proteostasis	Co-IP, recently described	[85,86]
PIN1	Cytoplasmic scaffold (cis-trans isomerase)	Phospho-S/T-P motifs at S215 (Neh7), S408 (Neh6/1 linker), S577 (Neh3)	Phospho-conformational switching; KEAP1 sequestration	Proline-directed kinases (CDK, MAPK)	Mutagenesis + cellular	[87,88,89]
PPIA/cyclophilin A	Cytoplasmic scaffold (PPIase)	Interdomain linker around trans-Pro174 (between Neh2 and Neh4)	Blocks KEAP1 access to NRF2 → stabilization	PPIase activity; cyclosporin-modulable	Co-IP + functional	[90]
CBP/p300	Nuclear coactivator (HAT)	Neh4 + Neh5 (TADs)	NRF2 acetylation; ARE chromatin opening	Acetyl-CoA, nutrient state	Mutagenesis + functional	[16,91,92]
RAC3/SRC-3/NCOA3	Nuclear coactivator (p160)	Neh4 + Neh5 (via pasB and R3B3 of RAC3)	Synergistic TAD activation; recruited to ARE chromatin	Hormone signaling, oncogenic amplification	Co-IP + functional	[20,21]
PRMT1/CARM1	Nuclear coactivator (arginine methyltransferase)	Neh4/5 (synergy with RAC3)	TAD methylation; cofactor synergy	SAM availability	Functional + drug-target validation	[20,21]
MED16 (Mediator)	Nuclear coactivator (transcription bridge)	Neh4/5 + Neh1	Tethers Mediator tail to NRF2; required for RNA Pol II CTD phosphorylation; abolishes 75% of NRF2 target genes when lost	General transcription machinery	Mutagenesis (Sekine et al.) + functional	[16]
CHD6	Chromatin remodeler	Neh1/Neh3 region	ARE-selective chromatin remodeling	Nucleosome positioning	Co-occupancy ChIP + functional	[93,94]
small Maf (MafF/G/K)	DNA-binding partner (obligate)	Neh1 (bZIP heterodimerization)	ARE recognition; obligate partner for DNA binding	Maf availability, stoichiometry	Structural (obligate heterodimer)	[17,95]
BACH1	DNA-binding competitor	Neh1 (competes with NRF2 for small Maf)	ARE repression at heme-responsive genes; ferroptosis switch	Heme levels (heme inactivates BACH1)	Structural + functional	[18,19,22,23]
RXRα	Nuclear receptor cross-modulator	Neh7	Repression of NRF2 transactivation	Retinoid signaling	Co-IP + functional (Wang 2013)	[96]
GR (glucocorticoid receptor)	Nuclear receptor cross-modulator	Neh4/5 region	Context-dependent NRF2 modulation	Glucocorticoid signaling	Co-IP/inferred	[97]
Prothymosin α (PTMA)	Nuclear coactivator	Neh1/Neh3 region	Enhances NRF2 transactivation; competes with KEAP1	Histone chaperone activity	Co-IP/inferred	[98]

Evidence tiers (column “Evidence level”): Tier I (structural + genetic), e.g., small Maf, CBP/p300, BACH1; Tier II (mechanistic with orthogonal validation), e.g., PIN1, MED16, PRMT1, RAC3/SRC-3, CHD6; Tier III (emerging/preliminary), e.g., PIPKIγ–HSP27, selected IQGAP1 and PPIA observations. Direct vs. indirect: partners that contact NRF2 directly are distinguished from indirect regulators that act through an intermediary (e.g., IQGAP1 via MEK–ERK; PIPKIγ via HSP27 phosphorylation of KEAP1; p62 via KEAP1 sequestration). The mechanism of indirect action is given in the “Functional output” column and detailed in Supplementary Table S2.

### 3.1. p62/SQSTM1 and NBR1: Condensate-Mediated KEAP1 Sequestration

p62 was introduced in Section 2.5 as a KEAP1-sequestration partner that binds the same Kelch pocket as the NRF2 ETGE through its KIR motif (DPSTGE) [40,41,42]. Beyond competitive inhibition, p62 oligomerizes through its PB1 domain and forms micron-scale liquid–liquid phase-separated condensates that physically immobilize KEAP1 within autophagy-targeted droplets [43]. The droplets are subsequently engulfed by autophagosomes, removing KEAP1 from the soluble pool entirely—a non-redox, condensate-driven NRF2 stabilization that operates whenever autophagy flux is impaired.

NRF2 is transcriptionally upregulated by phosphorylated p62 binding to KEAP1 through an ARE in the *SQSTM1* promoter, creating a positive feedback loop [41]: more p62 → more KEAP1 sequestration → more nuclear NRF2 → more p62 transcription. In autophagy-deficient hepatocytes, this drives constitutive NRF2 activation causally linked to hepatocellular carcinogenesis [24,44]. NBR1 harbors a region that interacts with KEAP1 and substitutes for p62 in some condensates [81]. TBK1 phosphorylates p62 at S349/S351, dramatically increasing its KEAP1 affinity [40,99]; thus, TBK1 inhibitors reduce NRF2 stabilization in autophagy-defective cancer cells, providing a partner-selective route to suppress constitutive NRF2 activity in HCC.

### 3.2. IQGAP1: A Ca^2+^-Responsive Scaffold and MEK–ERK Relay

IQGAP1 is a 189 kDa multi-domain scaffold protein with binding interfaces for actin, ERK2 [100], calmodulin, Cdc42/Rac1, and E-cadherin/β-catenin—a versatile cytoskeletal/signaling integrator extensively reviewed by Hedman and colleagues [101]. It was identified as a direct NRF2-binding partner by Kim and colleagues [102] using One-strep tag pulldown coupled with LTQ Orbitrap LC-MS/MS, with the interaction subsequently characterized at residue resolution to map to the IQ domain (aa 699-905) of IQGAP1, binding the Neh1/bZIP region of NRF2 [83]. Functionally, IQGAP1 binding stabilizes NRF2 (cycloheximide chase half-life extended ~2-fold), drives Ca^2+^-dependent nuclear translocation of the IQGAP1-NRF2 complex [102], and serves as a scaffold that brings MEK, ERK, and NRF2 into proximity [83,100]. IQGAP1 knockdown attenuates phenethyl isothiocyanate- and MEK-induced NRF2 activation [83]; IQGAP1’s broader carcinogenic activity in head and neck cancer further supports a disease-relevant role [84].

Three features make IQGAP1 a prototypical cytoplasmic scaffold partner. First, it decouples NRF2 activation from redox cues entirely: IQGAP1-mediated stabilization is triggered by intracellular Ca^2+^ rises rather than Cys151 modification of KEAP1. Second, it physically integrates two signaling pathways—MEK–ERK phosphorylation of NRF2 at S40 and Ca^2+^/calmodulin-driven nuclear translocation. Third, it explains a class of cellular contexts in which NRF2 is hyperactivated despite wild-type KEAP1 and NFE2L2. The TRPM2–IQGAP1–NRF2 axis in neuroblastoma [82] illustrates how this scaffolding contributes to chemoresistance: TRPM2 channel activity raises intracellular Ca^2+^, activating IQGAP1-mediated NRF2 nuclear translocation and doxorubicin resistance; depletion of either TRPM2 or IQGAP1 collapses NRF2 protein levels and re-sensitizes the cells.

IQGAP1 also illustrates that scaffolds extend the partner code beyond canonical Neh domains. Neh1 is the obligate dimerization surface for small Maf and the competition site for BACH1, but IQGAP1 occupies the same region in the cytoplasm and before small Maf engagement—temporally segregating its function from nuclear DNA-binding partners. This temporal-spatial separation is recurring: the same NRF2 surface can host different partners at distinct cellular locations and at different points along the activation trajectory.

Evidence strength and open questions (IQGAP1). The IQGAP1–NRF2 interaction is supported by co-immunoprecipitation and functional data in cancer cell lines ([82]; TRPM2-NRF2-IQGAP1 axis), but the direct binding interface on NRF2 has not been mapped at residue resolution. Peptidomimetic disruption of the IQGAP1 IQ domain remains entirely conceptual; no chemical lead exists. Whether IQGAP1 functions as a scaffold in non-cancer disease contexts requires independent validation.

### 3.3. PIPKIγ–HSP27: A Phosphoinositide-Coupled Chaperone Module

A recent study, first posted as a preprint and subsequently published in the Journal of Biological Chemistry (2025), identified an unexpected lipid-coupled mechanism of NRF2 stabilization [85]. Type I phosphatidylinositol phosphate kinase γ (PIPKIγ) generates phosphatidylinositol 4,5-bisphosphate (PI(4,5)P_2_) at defined membrane and perinuclear pools. The authors showed that PIPKIγ promotes an interaction between NRF2 and the chaperone HSP27, thereby increasing NRF2 stability and target-gene induction. This finding links phosphoinositide metabolism to NRF2 transcriptional control through a chaperone interface rather than through KEAP1 cysteine modification. This module was recently described and remains to be independently validated in additional cell systems.

This module is conceptually distinct because it connects a lipid second messenger to a transcription factor stability pathway via HSP27. It also provides a translational lead: the HSP27 antisense agent apatorsen (OGX-427) has reached Phase II testing in several cancers. Whether its clinical activity involves NRF2 destabilization remains untested and should be evaluated directly in archived trial material and prospective biomarker studies.

### 3.4. PIN1: Phospho-Conformational Switching Across Three Neh Domains

PIN1 is a peptidyl-prolyl cis–trans isomerase recognizing phosphorylated S/T-P motifs through its WW domain and catalyzing proline isomerization through its catalytic domain—a regulatory mechanism extensively reviewed in the foundational Lu and Zhou paper that established PIN1 as a pivotal phosphorylation-coupled signaling regulator [103]. NRF2 has three distinct phospho-recognition sites: S215 in Neh7, S408 in the Neh6/Neh1 linker, and S577 in Neh3 [88,94]. All three are phosphorylated by proline-directed kinases—primarily MAPKs and CDKs. Notably, PIN1 dysregulation has been implicated in the inverse epidemiological association between cancer (where PIN1 promotes oncogenic stabilization) and Alzheimer’s disease (where PIN1 loss exacerbates tau pathology) [104]—a directionality reversal that fits the partner-code framework’s prediction that the same partner can require activation in one disease and inhibition in another.

Two mechanisms of PIN1-mediated NRF2 stabilization have been documented. Direct mechanism: PIN1 binding induces a conformational change in NRF2 that reduces accessibility of NRF2 to the KEAP1 Kelch pocket and the β-TrCP phosphodegron, prolonging its half-life. Indirect mechanism: PIN1 binds KEAP1 itself through phospho-S/T-P motifs in the BTB and IVR domains, sequestering KEAP1 away from NRF2 [88]. PIN1 is overexpressed in many human cancers, including breast (100%), lung (67%), and bladder (56%) [105], and PIN1 directly binds and stabilizes NRF2 in triple-negative breast cancer to drive tumor progression [88]. PIN1 also maintains redox balance via a c-Myc/NRF2 axis in *KRAS*-driven pancreatic cancer [106], positioning PIN1 as a context-dependent partner across multiple cancer types. KPT-6566 (covalent, IC_50_ 640 nM, dual mechanism via Cys113 binding plus quinone release) [107] and sulfopin (covalent, double-digit nM, highly selective, validated *in vivo* in *MYCN*-driven neuroblastoma and pancreatic cancer) [108] represent the most advanced PIN1 inhibitors. Juglone, all-trans retinoic acid (ATRA), and arsenic trioxide (ATO) are additional PIN1 inhibitors in clinical use for unrelated indications.

### 3.5. PPIA/Cyclophilin A: A Structural KEAP1-Blocker Drugged by an FDA-Approved Compound

The most recent addition is peptidylprolyl isomerase A (PPIA; cyclophilin A), a cyclophilin-family peptidyl-prolyl isomerase with well-characterized biochemical and pharmacological properties [109,110]. Lu and colleagues [90] identified PPIA as an NRF2-binding partner that recognizes a hydrophobic region surrounding trans-Pro174 in the Neh4–Neh5 linker. Structural analysis showed that PPIA binding can occlude KEAP1 access and stabilize NRF2. This is one of the clearest examples of an interdomain linker acting as an active regulatory surface rather than as a passive spacer.

PPIA is therapeutically relevant because cyclosporin A (CsA), an approved immunosuppressant, disrupts the PPIA–NRF2 interaction. In *KEAP1*-mutant non-small-cell lung cancer (NSCLC) models, CsA promoted NRF2 ubiquitination, reduced glutamine-driven metabolic programs, and slowed tumor progression in patient-derived xenograft models [90]. These data support a repositioning hypothesis in NRF2-hyperactive cancer, although immunosuppression, calcineurin-dependent toxicity, and the therapeutic window must be addressed before clinical testing.

PPIA also illustrates a structural principle: NRF2 interdomain linkers can host partner interactions. The Neh4–Neh5 linker contains the PPIA site, the Neh6/Neh1 linker contains a PIN1 docking site at phospho-S408, and additional linker-proximal interactions are likely to emerge from proteomic and structural screens. These regions, therefore, deserve systematic mapping in future NRF2 interaction atlases.

### 3.6. Reading the Cytoplasmic Scaffold Logic

The partners surveyed here show that cytoplasmic NRF2 stability is an integration layer rather than a passive waiting state. Each partner translates a distinct upstream signal—autophagy flux (p62), Ca^2+^ and MEK–ERK activity (IQGAP1), phosphoinositide and chaperone state (PIPKIγ–HSP27), MAPK/CDK-dependent phospho-conformation (PIN1), or proline-isomerase-mediated KEAP1 competition (PPIA)—into extended NRF2 half-life and enhanced nuclear delivery. Several partners are connected to clinical-stage or approved agents, but most require direct NRF2 pharmacodynamic validation before they can be considered therapeutic targets.

## 4. The Nuclear Transcriptional Module: Partner Assembly at Chromatin

Once NRF2 reaches the nucleus, a second tier of the partner code determines which target genes are transcribed, how rapidly they respond, and how strongly they are induced. These outputs are not encoded solely in NRF2 abundance. They emerge from partners assembled on the transactivation domains and DNA-binding interface. This section organizes the nuclear module into four layers: small Maf-dependent DNA binding, BACH1-linked repression, Neh4/5 coactivator assembly, and chromatin or nuclear-receptor modulation.

### 4.1. The DNA-Binding Hub: An Obligate Dimer with a Built-In Repressor

NRF2 is a basic-leucine zipper (bZIP) transcription factor of the cap’n’collar (CNC) family, but it does not bind DNA as a monomer or homodimer. ARE recognition requires obligate heterodimerization with one of three small Maf proteins—MafF, MafG, or MafK—via the Neh1/CNC-bZIP domain [17,95,111]. The ARE core consensus, first defined as 5′-TGACNNNGC-3′ [112] and refined to the extended consensus 5′-TMAnnRTGAYnnnGCR-3′ [113], is bound by the NRF2–small Maf heterodimer through its bZIP basic region. The small Maf family was established as the obligate partner system for the entire CNC family by Itoh and colleagues in the founding 1995 study [111], and the MafF/G/K biology has been comprehensively reviewed [114,115]. This obligate dependence has two consequences. First, the stoichiometry of small Mafs is rate-limiting: in cells with low MafG/MafK levels, NRF2 nuclear translocation alone is insufficient to drive ARE-driven transcription. Second, the same bZIP surface is the competition site for BACH1—a CNC-family transcriptional repressor first characterized by Oyake and colleagues [116]—which heterodimerizes with the same MafF/G/K pool through MARE/ARE elements [18,19]. The cell contains a shared pool of small Mafs that is competed for by NRF2 (activator) and BACH1 (repressor).

The BACH1–NRF2–small Maf axis is a central example of partner-driven transcriptional specification. BACH1 is heme-regulated: increased intracellular heme binds BACH1 at multiple sites with distinct coordination structures [104,105], promoting nuclear export and proteasomal degradation [117]. The mechanism extends across the CNC family; BACH2 is regulated similarly and contributes to B-cell differentiation by controlling heme-responsive antibody gene expression [118]. Because BACH1 and NRF2 compete for small Maf partners at overlapping AREs, the BACH1:NRF2 ratio can determine whether iron-handling and ferroptosis-related genes are activated or repressed.

### 4.2. The Coactivator Complex: A Combinatorial Machine on Neh4/5

Once NRF2–small Maf heterodimers occupy ARE chromatin, the transactivation engine assembles on Neh4 and Neh5. This engine is a combinatorial complex whose composition determines transcriptional amplitude, kinetics, and gene selectivity. Five major components have been mapped to direct interactions with Neh4/5.

CBP and p300 were the first coactivators identified at NRF2’s transactivation domains [91]. Both are histone acetyltransferases (HATs) whose activation through transcription-factor dimerization has been structurally elucidated [119]. They bind Neh4 and Neh5 in a synergistic two-site manner through their KIX-CH1 surfaces, and both are recruited to ARE chromatin together with NRF2 after electrophile stimulation [16]. Their primary function is histone H3 and H4 acetylation at the ARE-flanking nucleosomes. CBP/p300 also acetylates NRF2 itself at multiple lysine residues in the Neh1 region, enhancing DNA binding and prolonging chromatin residence [92].

The acetylation marks placed by CBP/p300 are reversed by NAD^+^-dependent sirtuin deacetylases, providing the metabolic input that grounds the “chromatin/metabolic state” signal of Figure 1. SIRT1 deacetylates NRF2 at K588 and K591 in the Neh3 region, decreasing ARE binding and shifting NRF2 toward cytoplasmic relocalization [120]; SIRT7 deacetylates NRF2 at K443 and K518 to reduce KEAP1 binding and enhance nuclear translocation under oxidative stress [121]. These opposing readouts illustrate that the coactivator module is not only a writer but also an eraser of activating PTMs, with NAD^+^ availability gating the writer:eraser balance. The same logic extends to PRMT1/CARM1 (SAM-dependent writers, with no characterized arginine demethylase at NRF2) and to CBP/p300 (acetyl-CoA-dependent writers): the cellular pools of NAD^+^, SAM, and acetyl-CoA collectively gate the nuclear coactivator module’s output, establishing a direct biochemical link between metabolic state and NRF2 transcriptional amplitude.

RAC3/SRC-3/NCOA3 is the most thoroughly characterized p160-family coactivator at NRF2 [122,123]. Lin et al. [21] first demonstrated using a Gal4-Nrf2-(1–370) chimeric reporter system that RAC3 directly enhances NRF2 transactivation domain activity in cooperation with CBP/p300, p/CAF, PRMT1, and CARM1, with the combination producing transactivation amplitudes that none of the individual components achieves alone [21]. Kim et al. [20] subsequently mapped the direct interaction at residue resolution: the N-terminal pasB and C-terminal R3B3 domains of RAC3 bind Neh4 and Neh5 of NRF2 (verified by co-IP, FRET, and GST pulldown), and RAC3 is recruited together with NRF2 to the ARE enhancer of the HMOX1 promoter [20]. This biochemical synergy generates a testable prediction: breast and prostate tumors with NCOA3 amplification may show heightened NRF2-target gene output even when KEAP1 and NFE2L2 are wild-type, although a direct quantitative link between NCOA3 copy number and NRF2-target gene panel induction in matched human tumor specimens has not yet been systematically established.

PRMT1 and CARM1 add a third combinatorial layer through arginine methylation, working synergistically with RAC3 [21]. Protein arginine methylation as a mammalian regulatory mechanism has been comprehensively reviewed by Bedford & Clarke [124]. p/CAF (KAT2B) further extends the lysine-acetylation activity. Therapeutically, type I protein arginine methyltransferase (PRMT) inhibitors (GSK3368715, MS023) and CARM1 inhibitors (EZM2302) are in Phase I/II trials in acute myeloid leukemia (AML), multiple myeloma, and solid tumors and would be expected to suppress oncogenic NRF2 transactivation as a partner-selective effect (Section 7).

Mediator complex (anchored by MED16) completes the coactivator engine. The Mediator complex serves as a central integrator of transcription, bridging transcription factors to RNA Pol II [125,126]. Sekine et al. [16] demonstrated that MED16 directly binds both Neh4/5 and Neh1, and that *Med16* disruption attenuates the electrophile-induced expression of approximately 75% of NRF2 target genes without affecting hypoxia-responsive transcription. MED16 functions as a gene-selective conduit that tethers the Mediator tail submodule (MED23 and MED24) to NRF2 and enables RNA Pol II C-terminal domain phosphorylation [125,126]. Without MED16, NRF2 still binds DNA—but RNA Pol II is not productively recruited. This finding is critical: NRF2 transcriptional output is rate-limited not by DNA occupancy but by Mediator-mediated RNA Pol II coupling, which depends on a single Mediator subunit. Although MED16 is a mechanistically attractive node, the Mediator complex is challenging to drug in the near term; we therefore include *MED16* disruption as a conceptual rather than actionable interface in the partner-code pharmacopeia.

The combinatorial nature of this coactivator complex predicts that NRF2 transactivation depends on assembly state rather than on any single coactivator. This helps explain why NRF2 transcriptional amplitude can vary across tissues despite comparable nuclear NRF2 levels: different cells assemble different subsets of HATs, p160 coactivators, PRMTs, and Mediator subunits at Neh4/5. It also predicts that partner-selective modulation will be most effective when targeted to the coactivator component that is rate-limiting in each disease state.

### 4.3. Chromatin Remodelers and Transcriptional Fine-Tuners

Beyond the Neh4/5 coactivator hub, NRF2 engages partners that act on the chromatin context of ARE elements. CHD6, a chromodomain ATPase from the broader CHD family of chromatin remodelers [127], has been implicated in NRF2-associated antioxidant transcription and oxidative DNA damage responses [93,94]. Moore and colleagues showed that CHD6 is stabilized during oxidative stress and that CHD6 ablation impairs antioxidant transcriptional responses, including induction of NRF2 targets such as HMOX1 and TXNRD1 [93]. The extent to which CHD6 functions as a direct NRF2-domain-specific partner versus a broader chromatin-remodeling factor that supports NRF2 target gene induction requires clearer experimental distinction. The ancient regulatory architecture of the NRF2 chromatin program extends beyond canonical antioxidant genes [128]. Prothymosin α (PTMA) is a small acidic histone chaperone that binds the Neh1/Neh3 region and enhances NRF2 transactivation by modulating histone H1 displacement [98]. KAP1/TRIM28 has been reported to bind the Neh3 region with context-dependent effects, reflecting its dual roles as an HP1 recruiter (heterochromatin) and SUMO-driven coregulator at active enhancers.

### 4.4. Nuclear Receptor Cross-Modulators: Integrating Non-Redox Endocrine Signals

A fourth class of nuclear partners modulates NRF2 by integrating non-redox signals from nuclear receptors. RXRα is the prototype. Wang et al. [96] identified Neh7 as a direct binding interface for RXRα, demonstrating that RXRα binding occludes the neighboring Neh4/5 coactivator hub and represses NRF2 transactivation. The glucocorticoid receptor (GR) represses NRF2 transactivation through a partially overlapping Neh4/5–Neh7 region [97], suggesting that chronic glucocorticoid use can blunt the antioxidant response.

The S215 phospho-site recognized by PIN1 (Section 3.4) lies within the Neh7 RXRα-binding region. We propose that phosphorylation at S215 may protect NRF2 from RXRα by creating a competing PIN1 docking site. This would provide a single-residue example of partner switching: phospho-S215 favors PIN1-linked stabilization, whereas unphosphorylated S215 favors RXRα-linked repression. This hypothesis remains to be tested directly by residue-resolved interactomics and functional reporter assays.

### 4.5. Reading the Nuclear Module: Combinatorial Output from a Finite Partner Alphabet

The four nuclear layers share one organizing principle: NRF2 transcriptional output emerges from a finite partner alphabet assembled at a limited set of surfaces. Three implications follow. First, the same nuclear NRF2 pool can drive different transcriptional programs in different cell types because the available partners differ. Second, NRF2 hyperactivation in cancer cannot be understood solely from KEAP1 status; it also depends on BACH1, small Maf proteins, RAC3, CBP/p300, PRMTs, MED16, and chromatin state. Third, the nuclear module offers the most direct route to inhibit oncogenic NRF2 without altering NRF2 protein stability.

## 5. The Neh-Domain Partner Atlas: A Coordinate System for the Partner Code

**Figure 3:**
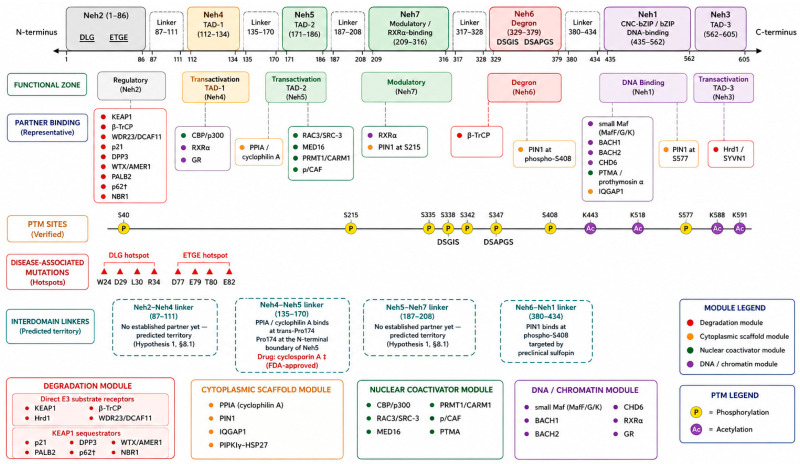
The NRF2 Neh-domain partner atlas. The 605-residue human NRF2 (NFE2L2) is shown with its seven Neh domains and interdomain linkers: Neh2 (DLG/ETGE degrons), Neh4/Neh5 (transactivation), Neh7 (RXRα-binding), Neh6 (β-TrCP phosphodegrons), Neh1 (CNC-bZIP DNA-binding), and Neh3 (C-terminal transactivation). Partners are mapped to experimentally supported binding regions and color-coded by module; verified phosphorylation and acetylation sites are marked where they define docking or output. Four organizing patterns are highlighted: degradation and activation surfaces are spatially segregated; antagonistic partners can share a domain; recurrent cancer mutations cluster at trafficked interfaces; and interdomain linkers are emerging as a regulatory territory. Binding confidence levels are detailed in Table 2 and Supplementary Table S2. Module color coding: degradation module (red), cytoplasmic scaffold module (amber), nuclear coactivator module (green), and DNA/chromatin module (purple). Symbols: a yellow circle labeled “P” denotes a verified phosphorylation site, and a purple circle labeled “Ac” denotes a verified acetylation site. A red triangle marks a recurrent cancer-associated hotspot for gain-of-function mut. Dashed boxes around the interdomain-linker regions denote predicted partner territory that is currently under-mapped (Hypothesis 1, Section 8.1), as distinct from the solid-bordered domains with experimentally established partners. The double-dagger (‡) denotes an FDA-approved drug that engages the indicated interface—here, cyclosporin A, which targets the PPIA-binding site at trans-Pro174 in the Neh4–Neh5 linker.

Section 2, Section 3 and Section 4 introduced individual partners and their mechanisms. Section 5 takes an alternative view: rather than walking through partners again, we ask what becomes visible only when all partners are simultaneously projected onto the linear NRF2 sequence. Figure 3 displays this projection—22 partners (cataloged comprehensively in Supplementary Table S1), 8 recurrent disease-associated mutations, and the principal phosphorylation and acetylation sites mapped onto the 7 Neh domains and the disordered linkers between them. Four organizing patterns emerge from the atlas, and they would not be apparent from any single partner study. The remainder of this section discusses these four patterns; specific partner mechanisms are not re-derived here, and the reader is referred to Section 2, Section 3 and Section 4.

### 5.1. Pattern I—Spatial Segregation of Degradation and Activation Logic

Neh2 (residues 1–86) is the most extensively studied region of NRF2 and the locus where the canonical KEAP1-driven partner code is established. It harbors two short degron motifs—the high-affinity ETGE (residues 77–82) and the low-affinity DLG (residues 23–31)—that together constitute the “two-site recognition” interface for the KEAP1 Kelch domain [34,35]. KEAP1 dimers engage one NRF2 molecule through both motifs simultaneously, positioning a cluster of seven lysines between DLG and ETGE for CUL3-RBX1-mediated ubiquitination [2,33].

Yet Neh2 is not a KEAP1-exclusive zone. Several proteins compete with KEAP1 for the same interface and thereby stabilize NRF2 without altering its synthesis: p21/CDKN1A binds the DLG motif and antagonizes KEAP1 engagement [46]; DPP3 carries an ETGE-like motif that displaces NRF2 from KEAP1 in stress and cancer contexts [48,49]; and WTX/AMER1 and PALB2 sequester KEAP1 itself through their own ETGE-like degrons, indirectly raising free NRF2 levels [45,47,50]. The p62/SQSTM1 receptor occupies a related but distinct position: rather than binding NRF2 directly, its KIR motif binds the same KEAP1 Kelch pocket that recognizes the NRF2 ETGE, sequestering KEAP1 into autophagy-associated condensates and freeing NRF2 [40,41,42].

The clinical significance of this hub is substantial. Cancer-associated NRF2 gain-of-function mutations are concentrated in the DLG and ETGE motifs [65,66], with R34, D29, W24, L30, D77, E79, T80, and E82 recurring across NSCLC, head and neck, esophageal, and bladder carcinomas. These mutations do not create a new NRF2 program; they erase the KEAP1-recognition entry while leaving downstream coactivator interactions intact.

### 5.2. Pattern II—Antagonistic Partners Share Single Domains

The atlas reveals a recurring architectural motif: the same domain frequently hosts partners with opposite functional outputs, creating intrinsic switches embedded in NRF2 itself. At Neh2, KEAP1 (degradation) competes with p21/p62/DPP3 (stabilization through KEAP1 sequestration). At Neh1, the obligate small Maf heterodimer (activation) competes with BACH1 (repression) for the same MafF/G/K pool—the BACH1:NRF2 ratio at small-Maf-dependent AREs determines ferroptosis sensitivity (Section 4.1 and Section 6.2). At Neh7, RXRα (repression) and PIN1 phospho-S215 (stabilization) compete for an overlapping, disordered surface, resulting in a single-residue partner switch (Section 4.4). At the Neh6/Neh1 linker, β-TrCP (degradation) and PIN1 (stabilization) compete for distinct phospho-states of the same residue cluster. This architecture means that perturbing partner stoichiometry—not just NRF2 abundance—can flip output, explaining why tissues with similar nuclear NRF2 levels can show qualitatively different transcriptional programs depending on partner availability.

### 5.3. Pattern III—Disease Mutations Concentrate at the Most Heavily Trafficked Interfaces

Projecting cancer-associated NRF2 gain-of-function mutations onto the atlas reveals a marked spatial concentration. Recurrent missense mutations in NSCLC, HNSCC, esophageal cancer, and HCC cluster near the DLG (W24, D29, L30, R34) and ETGE (D77, E79, T80, E82) motifs of Neh2 [26,60,65,66], the residues that mediate KEAP1 Kelch-pocket engagement [51,52]. In contrast, fewer recurrent mutations are found in Neh4/5, Neh1, or Neh3. This pattern is instructive: cancer most often rewires NRF2 by disabling its degradation code while preserving its transcriptional machinery.

### 5.4. Pattern IV—Interdomain Linkers and the C-Terminal Region as Emerging Territory

Until recently, the disordered linkers between Neh domains were often treated as passive spacers. Two findings argue against that view. PPIA binds a hydrophobic sequence centered on trans-Pro174 in the Neh4–Neh5 linker and can interfere with KEAP1 access [90]. PIN1 binds the phospho-S408 site in the Neh6/Neh1 linker as one of three PIN1 docking points on NRF2 [87,88]. The PIPKIγ–PI(4,5)P_2_–HSP27 complex may involve additional linker-proximal surfaces [85]. These examples suggest that linker regions should be incorporated explicitly into NRF2 structural and pharmacological maps.

### 5.5. What the Atlas Adds Beyond the Partner-by-Partner Sections

The four patterns above are visible only when partners are displayed simultaneously on a shared coordinate system. Pattern I (spatial segregation) explains why partner-selective pharmacology is possible at all. Pattern II (antagonistic partners at single domains) explains why partner stoichiometry, not NRF2 abundance, sets transcriptional output. Pattern III (mutation clustering) confirms that disease pathology arises through partner-code disruption. Pattern IV (linker territory) identifies where the next generation of partners and druggable interfaces will be found. None of these patterns is fully derivable from any single partner study—they are emergent properties of the integrated atlas. In the disease and therapeutic sections that follow, we analyze each disease and each drug class using these four atlas-level patterns rather than individual partner mechanisms. The atlas relates to prior efforts in a specific way. The Poh et al. interactome [28] reported binding affinities for 46 new NRF2 partners but presented them as a partner-protein matrix without protein-level spatial mapping; the Nam and Keum et al. catalog [27] assigned partners as coactivators or corepressors without a domain coordinate system. The atlas presented here repositions each partner onto NRF2’s primary sequence so that competition, antagonism, mutation clustering, and linker territory become directly readable. This positioning is the conceptual core of the present review and is distinct from the chronological drug-discovery synthesis of Zhang et al. [29].

## 6. Disease Rewiring of the Partner Code

**Figure 4:**
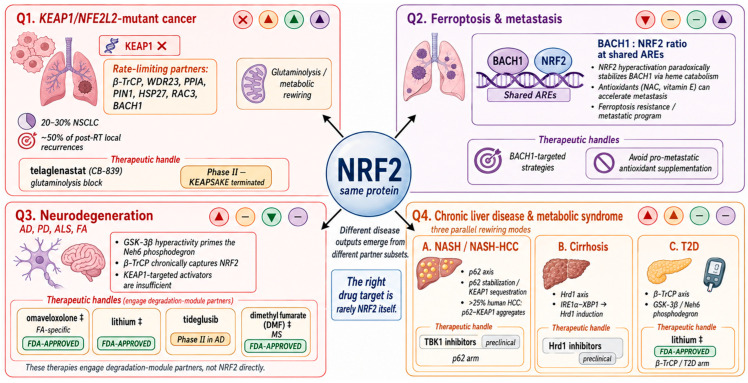
Disease rewiring of the NRF2 partner code. The four-module framework is rewired across disease contexts; perturbation icons indicate whether a module is hyperactive, suppressed, mutated, or self-reinforcing. In *KEAP1*/*NFE2L2*-mutant cancer, KEAP1 loss shifts dependency toward β-TrCP, WDR23, PPIA, PIN1, HSP27, BACH1, and coactivators. In ferroptosis-linked disease, the BACH1:NRF2 ratio at small-Maf AREs sets iron and lipid peroxidation responses. In neurodegeneration, GSK-3β–β-TrCP may restrain NRF2. In chronic liver and metabolic disease, three modes co-exist: (A) p62-driven hyperactivation (NASH/HCC), (B) Hrd1-driven suppression (cirrhosis), and (C) β-TrCP/Neh6-driven degradation (type 2 diabetes). Drug-stage badges distinguish approved, clinical-stage, and preclinical agents. The central principle: the most useful target is often the rate-limiting partner, not NRF2 itself. Perturbation symbols: a filled up-triangle (▲) denotes a hyperactive module; a filled down-triangle (▼), a suppressed module; a cross (✕), a mutated or genetically inactivated module; and a minus sign within the circle (–), a module that is not appreciably altered in that context. The module color circles in the upper-right corner of each quadrant indicate which of the four modules is perturbed: degradation (red), cytoplasmic scaffold (amber), nuclear coactivator (green), and DNA/chromatin (purple). Drug-stage badges: a double-dagger (‡) with a green badge marks an FDA-approved agent; an amber badge, a Phase I–III clinical-stage agent; and a gray badge, a preclinical agent.

The framework developed in Section 2, Section 3, Section 4 and Section 5 is not a static catalog. In disease, the partner code is rewired: specific partners become hyperactive or suppressed, mutations remove one layer of the code while preserving others, and the same NRF2 protein can produce distinct transcriptional programs depending on the partner subset deployed. This view refines classical interpretations of NRF2 in cancer and chronic disease by identifying which partner, rather than which pathway label, is rate-limiting in each context (Figure 4 illustrates these disease-specific rewirings).

Tier I—Strongly supported disease rewiring

The following two subsections describe disease contexts in which partner-code rewiring is supported by multiple independent lines of human, genetic, and mechanistic evidence: *KEAP1*/*NFE2L2*-mutant lung cancer and the BACH1:NRF2 axis in ferroptosis-prone tissues.

### 6.1. KEAP1/NFE2L2-Mutant Cancer: Oncogenic NRF2 with Intact Downstream Machinery

Approximately 20–30% of NSCLC tumors harbor KEAP1 loss-of-function or NFE2L2 gain-of-function alterations [57,58,60,61,62,65,66], with prevalence supported by pan-cancer analyses [26,64]. Both alteration classes uncouple NRF2 from the canonical KEAP1 turnover pathway. The partner-code framework adds a critical point: only one layer of the code is corrupted, whereas the remaining partner interfaces remain intact and may become rate-limiting. This is consistent with the observation that *KEAP1*-mutant cancers can remain dependent on PPIA, PIN1, HSP27, BACH1, and nuclear coactivators.

The transcriptional output of NRF2 hyperactivation in this context is dominated by a metabolic reprogramming program in which SLC1A5 (ASCT2, glutamine transporter) and the transcription factor KLF5 are jointly upregulated by NRF2 and cooperate to sustain glutaminolysis [90,99,129], aligning with the metabolic reprogramming output indicated in Figure 1. This output is the proximal driver of the glutamine-addicted phenotype that motivates the downstream glutaminase-inhibitor trials discussed below.

This reframing motivates a therapeutic logic that may be distinct from direct NRF2 inhibition. Glutaminase inhibition with telaglenastat (CB-839) has been evaluated in *KEAP1*/*NFE2L2*-mutant NSCLC through clinical programs including KEAPSAKE (NCT04265534), reported as terminated for lack of clinical benefit, and BeGIN (NCT03872427), listed as active/not recruiting or closed to accrual depending on registry view. These trials targeted the metabolic dependency created by NRF2 hyperactivation, particularly in *LKB1*-deficient backgrounds. The mixed clinical outcome indicates that downstream metabolic targeting is biologically rational but insufficient without more precise partner-code and metabolic biomarker stratification.

A second logic is to target partner interfaces that remain active after *KEAP1* loss. *KEAP1*-mutant NSCLC retains dependence on PPIA in models in which cyclosporin A disrupts PPIA–NRF2 binding and destabilizes NRF2 [90]. PIN1 inhibitors have shown activity in *KEAP1*-mutant lung and triple-negative breast cancer models [89,107,108]. HSP27, RAC3/SRC-3, and CBP/p300 represent additional candidate dependencies, although direct NRF2 engagement should be verified for each therapeutic context. The central point is that KEAP1 loss does not make NRF2 undruggable; it shifts tractability from the degradation module to scaffold, coactivator, and metabolic modules.

Binkley et al. [130] showed that *KEAP1*/*NFE2L2*-mutant NSCLC accounts for a large fraction of in-field local recurrences after stereotactic body radiotherapy and that glutaminase inhibition can radiosensitize these tumors through glutathione depletion. A partner-code interpretation is that DNA damage may activate additional NRF2-stabilizing inputs, including the PALB2–BRCA1 axis [47,50], thereby adding a second induction layer in irradiated tumors. Evidence strength: this disease context is among the strongest in the review because it links recurrent human genomics, mechanistic biology, xenograft data, and early clinical testing.

### 6.2. Ferroptosis and Iron Metabolism: The BACH1:NRF2 Ratio at Small-Maf-Dependent AREs

The observation that NRF2 induces, whereas BACH1 represses, overlapping ferroptosis-protective genes—including FTH1, FTL, SLC7A11, GPX4, and FSP1—reframes ferroptosis as a partner-ratio problem as much as a redox-stress problem [22,23,131,132,133]. Ferroptosis biology rests on at least two convergent protective arms: GPX4-mediated detoxification of lipid peroxides [134] and FSP1-dependent CoQ10-based suppression of lipid peroxidation [135]. In the partner-code framework, whether these arms are engaged depends on the balance between NRF2–small Maf activation and BACH1–small Maf repression.

Two back-to-back Cell studies in 2019 clarified the therapeutic implications. Lignitto et al. [22] showed that *KEAP1*-mutant lung cancers can stabilize BACH1 through NRF2-driven HO-1 activity, which catabolizes heme that would otherwise promote BACH1 export and degradation. Wiel et al. [23] showed that N-acetylcysteine and vitamin E accelerated metastasis in mouse models through the NRF2–BACH1 axis. Together, these studies explain how antioxidant exposure can have opposite effects at different stages of cancer progression.

A potential therapeutic implication, which remains to be tested, is that dual NRF2-activator/BACH1-inhibitor pharmacology may outperform pure NRF2 activation in selected ferroptosis-relevant contexts. HPPE, cannabidiol quinones with dual BACH1/NRF2 activity, and hemin-mimetic strategies that promote BACH1 nuclear export remain preclinical. The clinical opportunity is broad but not yet mature, spanning ischemia–reperfusion injury, neurodegeneration, intervertebral disc degeneration, pulmonary hypertension, and therapy-resistant cancer. For each indication, the BACH1:NRF2 ratio should be tested as a biomarker rather than assumed.

A second ferroptosis-relevant rewiring involves p62. The p62–KEAP1–NRF2 axis protects against ferroptosis in HCC [25] and has been implicated in diabetic wound healing. NRF2 induction of p62 creates a positive feedback loop that may reinforce ferroptosis resistance, particularly in NASH-to-HCC progression. Evidence strength: the BACH1–NRF2 mechanism is supported by strong biochemical and mouse model evidence, but its disease-level translation outside lung cancer remains hypothesis-generating.

Tier II—Mechanistically plausible but incompletely translated

The contexts below—neurodegeneration (including Friedreich’s ataxia) and chronic liver disease—show mechanistically coherent partner-code rewiring in cell and animal models, but disease-stage human evidence and pharmacodynamic biomarkers remain limited.

### 6.3. Neurodegeneration: The GSK-3β/β-TrCP Axis as a Rate-Limiting Partner

In Alzheimer’s disease, Parkinson’s disease, amyotrophic lateral sclerosis (ALS), and Huntington’s disease, NRF2 protein levels and target-gene output often decline with aging and disease progression [136,137], and genetic reduction in Nrf2 worsens AD-like phenotypes in mouse models [138]. The partner-code framework identifies the GSK-3β/β-TrCP/Neh6 axis as a plausible rate-limiting module in these settings [139,140,141]. Salazar et al. [142] first showed that GSK-3β phosphorylates NRF2 within Neh6, enabling β-TrCP-dependent degradation independently of KEAP1. Because GSK-3β is hyperactive in multiple neurodegenerative contexts, excessive Neh6 priming may restrain NRF2 when antioxidant and proteostatic programs are needed.

Our disease-specific reading suggests that KEAP1 appears largely intact in neurodegeneration, while the β-TrCP arm may be constitutively engaged because GSK-3β hyperactivity (driven by reduced PI3K-AKT signaling in aging neurons, by Aβ-driven kinase activation in AD, and by α-synuclein-driven kinase activation in PD) keeps the Neh6 phosphodegron primed. NRF2 is therefore degraded as fast as it is synthesized, regardless of redox status. The proteostasis crisis intrinsic to neurodegeneration further amplifies this loss [140]. This is consistent with the observation that pure KEAP1-targeted activators show only modest efficacy in late-stage neurodegeneration.

Three therapeutic implications follow. First, GSK-3β inhibitors may be more effective NRF2 activators than KEAP1-targeted compounds in neurodegeneration. Tideglusib reached Phase II in AD [143] and is in development for ALS and progressive supranuclear palsy; lithium has long been used in bipolar disorder and shows GSK-3β-driven NRF2 stabilization in preclinical AD models. Second, dual GSK-3β-inhibitor/NRF2-activator hybrid molecules have shown additive neuroprotection [69,144]. Third, dimethyl fumarate (Tecfidera, FDA-approved for MS) owes part of its efficacy to KEAP1 Cys151 modification, but its activity in MS likely depends on its ability to overcome both the KEAP1 and β-TrCP brakes simultaneously [145,146].

Friedreich’s ataxia (FA) is the proof-of-concept disease for partner-code-guided therapy. Despite oxidative stress, NRF2 levels are paradoxically reduced in FA patient cells, with KEAP1 upregulation and hyperactivation of the GSK-3β axis. Omaveloxolone (SKYCLARYS) became the first FDA-approved drug for FA in February 2023 based on the MOXIe trial showing significant modified Friedreich Ataxia Rating Scale (mFARS) improvement [147,148]. A single layer of the code (KEAP1) is targeted, and clinical benefit follows. We propose that combining omaveloxolone with a GSK-3β inhibitor should produce additive effects in FA. Evidence strength: preclinical and mechanistic evidence for the GSK-3β/β-TrCP axis in neurodegeneration is strong; FA represents the only disease in this category with a partner-code-validating approved therapy. For Alzheimer’s disease (AD), Parkinson’s disease (PD), and amyotrophic lateral sclerosis (ALS), clinical translation of the framework remains preliminary, with Phase II GSK-3β inhibitor trials providing the most directly relevant data.

### 6.4. Metabolic Disease and Chronic Liver Pathology: p62-Axis and Hrd1 Paradox

Metabolic and liver diseases illustrate why the partner code is not reducible to “more NRF2 is better.” In autophagy-deficient livers, p62 accumulates, sequesters KEAP1, and sustains NRF2 activation. In autophagy-deficient livers, p62 accumulates, sequesters KEAP1, and sustains NRF2 activation. Komatsu and colleagues showed that this axis drives hepatocellular carcinoma in autophagy-impaired mice. Komatsu and colleagues showed that this axis drives hepatocellular carcinoma in autophagy-impaired mice [24,40,41,42,44]. In nonalcoholic steatohepatitis (NASH), p62/NRF2 signaling may initially protect against oxidative injury but later promote metabolic reprogramming, inflammation, and tumor initiation if autophagic flux remains impaired.

Tier III—Exploratory extensions

The applications below are exploratory: the partner-code framework offers plausible mechanistic hooks, but disease-specific evidence is sparse, often confined to single studies, single model systems, or analogical reasoning from related contexts.

Hrd1 represents an opposing liver-disease logic. In cirrhosis, IRE1α–XBP1 induces Hrd1, which directly ubiquitinates NRF2 and suppresses cytoprotective transcription despite ongoing oxidative stress [14]. This creates a paradox: a diseased liver can contain both NRF2-hyperactivation states driven by p62 and NRF2-suppression states driven by Hrd1, depending on disease stage, cell type, and proteostasis status.

The therapeutic implication is stage-specific. In early stages of steatohepatitis or in ferroptosis-prone hepatocytes, NRF2 activation may be protective. In autophagy-deficient preneoplastic hepatocytes with p62 accumulation, suppressing p62 phosphorylation, restoring autophagy, or targeting downstream oncogenic outputs may be preferable. In cirrhosis with Hrd1 induction, protecting NRF2 from Hrd1-mediated degradation may be more rational than additional KEAP1 inhibition. Evidence strength: p62-driven HCC biology is supported by strong genetic mouse model data; Hrd1-mediated NRF2 suppression is supported by mechanistic liver disease models; clinical translation remains early.

In type 2 diabetes (T2D) and metabolic syndrome, the β-TrCP/Neh6 axis becomes dominant. Chronic insulin resistance reduces PI3K-AKT signaling, de-represses GSK-3β, primes the Neh6 phosphodegron, and leads to β-TrCP-driven NRF2 degradation [11,149]; the same axis underlies pancreatic β-cell dysfunction [71]. A related metabolic rewiring—the PIPKIγ-HSP27 axis—has been implicated in cancer cachexia and chemoresistance in chemotherapy-stressed tissues [85]; HSP27 antisense (apatorsen) has been tested at Phase II for castration-resistant prostate cancer.

The framework’s partner-code reading extends, more tentatively, to chronic kidney disease (CKD) [150], cardiovascular pathology [151], and pulmonary diseases (chronic obstructive pulmonary disease, COPD; idiopathic pulmonary fibrosis, IPF; asthma) [152]. In each of these contexts, partner-specific NRF2 dysregulation may be invisible to KEAP1-only readouts, but disease-stage human evidence and pharmacodynamic biomarkers are currently sparse, and the application of partner-code remains exploratory.

The framework also intersects with two broader biological dimensions captured in earlier comprehensive surveys: NRF2’s mitochondrial-protective functions [153,154], which converge with the β-TrCP/Neh6 axis in neurodegeneration and metabolic disease, and the evolutionary conservation of CNC-bZIP stress-response biology from *Drosophila* intestinal stem cells to mammalian aging [155,156]. The integrated NRF2-ome web resource [157] catalogs partner-code-relevant protein–protein and signaling interactions, target genes, transcription factors that regulate transcription, and miRNAs, providing a community-curated lookup tool. Subsequent work has highlighted the relevance of the NRF2 ’addiction’ phenotype in the tumor microenvironment, particularly in lung cancer [158], against which the framework can be applied.

Evidence strength varies across these applications. The p62-KEAP1 axis in NASH/HCC is supported by strong human histopathological data (>25% of HCC specimens show p62-KEAP1 aggregates) and validated mouse models. The Hrd1-cirrhosis and β-TrCP-T2D mechanisms are mechanistically grounded but have limited clinical translation data. The broader extension to cardiovascular, pulmonary, and renal disease is currently exploratory and should be interpreted as hypothesis-generating rather than evidence-based.

### 6.5. Reading the Disease Rewiring: Four Principles

First, disease specificity emerges from which partner becomes rate-limiting, not from NRF2 abundance alone. In *KEAP1*/*NFE2L2*-mutant lung cancer (Section 6.1), the degradation module is genetically disabled, and constitutive NRF2 output is then shaped by intact downstream partners (β-TrCP, WDR23, PPIA, PIN1, HSP27, BACH1, and the coactivator complex). In neurodegeneration (Section 6.3), the KEAP1 axis is largely preserved, but the β-TrCP/Neh6 axis is constitutively engaged because chronic GSK-3β hyperactivity primes the Neh6 phosphodegron. In autophagy-deficient liver disease (Section 6.4), p62 accumulation drives NRF2 hyperactivation by sequestering KEAP1, whereas Hrd1 induction during cirrhosis suppresses NRF2 from the ER side. The same nominal pathway thus produces opposite NRF2 dysregulation states depending on which partner is the rate-limiting node.

Second, the same partner can require opposite modulation directions in different diseases. BACH1 (Section 6.2) is the clearest example: BACH1 destabilization is therapeutically desirable in ferroptosis-prone neurodegeneration and ischemic tissue because it derepresses the NRF2:small-Maf antioxidant program, whereas in *KEAP1*-mutant lung cancer, BACH1 stabilization drives metastasis and represents a target for inhibition rather than relief. p62 follows a similar bidirectional logic, being protective in early ferroptosis-prone hepatocytes but oncogenic when accumulated in autophagy-deficient preneoplastic livers. A partner-code reading, therefore, requires both the identification of the dominant partner and the assignment of the modulation direction appropriate to that disease context.

Third, several approved or clinically tested drugs intersect partner-code nodes even though their approved indications are not NRF2-related. Omaveloxolone and dimethyl fumarate activate the KEAP1 module in approved indications. Lithium and cyclosporin A engage β-TrCP/GSK-3β and PPIA axes, respectively, in ways that could be evaluated through biomarker-stratified repositioning. CCS1477 and GSK3368715 target nuclear coactivator-adjacent dependencies. A partner-code reading, therefore, separates mechanistic plausibility from clinical readiness.

Fourth, evidence strength varies sharply across disease contexts, and the framework should not be applied uniformly across them. The p62-KEAP1 axis in NASH/HCC and the *KEAP1*-mutant cancer paradigm are supported by strong human histopathological, genetic, and mouse-model evidence (Tier I). The BACH1:NRF2 ratio in ferroptosis-prone tissues and the GSK-3β/β-TrCP axis in selected neurodegenerative contexts have mechanistic support but limited stratified clinical data (Tier II). Extensions to chronic kidney, cardiovascular, and pulmonary disease, while mechanistically plausible, remain hypothesis-generating (Tier III). Biomarker stratification—rather than broad NRF2 activation—therefore defines clinical applicability, and the appropriate evidence tier should be matched to the strength of the claim.

## 7. Therapeutic Implications: A Partner-Selective NRF2 Pharmacopeia

The four-module structure of the partner code can be read as a map of pharmacologically addressable interfaces. Each module contains protein–protein interactions, post-translational modifications, or signaling dependencies that can be targeted by approved drugs, clinical-stage agents, or preclinical tools. This section organizes the existing pharmacology around the partner-code structure (Table 4), distinguishes four levels of translational maturity, and outlines biomarker-stratified combination logic. The therapeutic literature on NRF2 activators and inhibitors provides the historical scaffold [29,32], but the partner-code framework reorganizes that pharmacology around disease-specific rate-limiting partners. Throughout this section, the therapeutic proposals are repositioning and mechanistic hypotheses rather than clinical recommendations: each agent carries an explicit evidence-origin label—(M) mechanistic, (A) animal-model, (EC) early clinical, or (CT) established clinical trial evidence (defined in the footnote to Table 4 and applied per agent in Table 4)—and readers should inspect this label, together with the Tier I/II/III evidence grade of the underlying partner interaction, before considering any specific agent for translational development. Caution is warranted for proposals resting solely on mechanistic evidence (e.g., IQGAP1- and MED16-directed strategies) and for repurposing agents whose established clinical use is unrelated to NRF2 (e.g., cyclosporin A, lithium, PRMT inhibitors).

**Table 4:** Druggable NRF2 protein–protein interactions: a partner-selective pharmacopeia.

Target Interface	Mechanism	Lead Compounds	Therapeutic Concept	Indication	Stage	NRF2 Partner Engagement	Refs.
T1; KEAP1–NRF2 (Neh2) Cys151 modifiers	Covalent KEAP1 inactivation	Omaveloxolone ‡; DMF ‡; sulforaphane; bardoxolone methyl; CDDO derivatives	NRF2 activation	FA (omaveloxolone); MS (DMF); CKD/PAH (bardoxolone)	Approved (omaveloxolone, DMF); Phase III terminated (bardoxolone) (M; A; CT)	Yes—direct (canonical)	[145,147]
T4; KEAP1–NRF2 (Neh2) Kelch PPI inhibitors	Direct PPI block at Kelch domain	KI-696; ML334; newer monoacidic chemotypes	NRF2 activation, non-electrophilic	Research probes; preclinical	Preclinical (M)	Yes—direct (canonical)	[159,160]
T2; β-TrCP–NRF2 (Neh6)	GSK-3β inhibition (upstream)	Lithium ‡; tideglusib; CHIR-99021	NRF2 stabilization in insulin resistance, neurodegeneration	T2D, AD, PD, ALS	Approved (Li); Phase II (tideglusib) (M; A)	Inferred—via GSK-3β axis	[69,70,142,144]
T4; Hrd1–NRF2	Hrd1 RING inhibition; IRE1α–XBP1 axis blockade	LS-102 and related	NRF2 preservation in cirrhosis, renal IRI	Cirrhosis, ischemia–reperfusion injury	Preclinical (M)	Inferred—functional KD studies	[14]
T4; WDR23–NRF2	DCAF11/WDR23 disruption; PROTAC	Early-stage degraders, peptide blockers	Selective nuclear NRF2 destabilization	Aging, *KEAP1*-mutant cancer	Preclinical (early) (M)	Inferred—functional studies	[15,39]
T3/4; p62–KEAP1 (KIR motif)	p62 phospho-S349 disruption; TBK1 inhibition	TBK1 inhibitors (BX795, momelotinib), p62 mimetic peptides	Suppress NRF2 in autophagy-deficient HCC	HCC, NASH-driven cancer	Preclinical (M; A)	Yes—direct (canonical)	[42,99]
T2; PPIA–NRF2	Direct PPI disruption at trans-Pro174	Cyclosporin A ‡	NRF2 destabilization in *KEAP1*-mutant cancer	*KEAP1*-mutant NSCLC	Approved (drug repositioning) (M)	Yes—direct; CsA inferred via PPIA	[90]
T2/4; PIN1–NRF2	Covalent Cys113 PIN1 inhibition	Sulfopin; KPT-6566; juglone; ATRA ‡; arsenic trioxide ‡	Block oncogenic NRF2 stabilization	*KEAP1*-mutant NSCLC, TNBC, MYCN-NB	Preclinical (sulfopin); approved (ATRA, ATO) (M; A)	Yes—direct binding established	[87,107,108]
T3; HSP27/PIPKIγ–NRF2	HSP27 antisense; lipid kinase inhibition	OGX-427 (apatorsen)	NRF2 destabilization in chemoresistant tumors	CRPC, NSCLC	Phase II completed/terminated; repositioning hypothesis only (M; EC)	Inferred—HSP27 modulates NRF2 indirectly	[85,86]
T4; IQGAP1–NRF2	IQ-domain peptide blockers; indirect via Ca^2+^/MEK-ERK	Early peptidomimetics; trametinib ‡; cobimetinib ‡	Suppress NRF2 in Ca^2+^-driven chemoresistance	Neuroblastoma (TRPM2 axis), HCC	Concept stage (M)	Conceptual—peptidomimetic	[83,102]
T3; CBP/p300–NRF2 (Neh4/5)	HAT inhibition or PPI block	A-485; CCS1477 (inobrodib)	Reduce oncogenic NRF2 transactivation	*KEAP1*/*NFE2L2*-mutant cancer; AML	Phase I/II (M; EC)	Yes—validated NRF2 coactivator	[91,92,161]
T4; RAC3/SRC-3–NRF2 (Neh4/5)	SRC-3 SMI; degradation	SI-2 and fluorinated analogs; bufalin; phospho-bufalin (p-Buf); gossypol; verrucarin A	Indirect oncogenic NRF2 suppression in SRC-3-amplified cancers	Breast (TNBC), prostate, ovarian	Preclinical (M)	Inferred—NRF2-specific engagement not validated	[20,162]
T3; PRMT1/CARM1–NRF2	Type I PRMT/CARM1 inhibition	GSK3368715; MS023; EZM2302	Disrupt coactivator synergy	AML, MM, solid tumors	Phase I/II (M; EC)	Inferred—via coactivator complex	[124]
T4; MED16-Mediator axis	Disruption of MED16-MED23/24 submodule; targeted degradation	Concept stage	Suppress 75% of NRF2 target genes with high gene-selectivity	*KEAP1*-mutant cancer	Concept stage (M)	Yes—direct (Sekine); CONCEPTUAL ONLY	[16]
T4; BACH1–small Maf axis	BACH1 inhibition; HO-1 inhibition; heme delivery	HPPE; CBD-quinones; hemin; ZnPP/SnPP	Selectively boost antioxidant/iron program; block metastasis	Pulmonary hypertension, lung cancer metastasis, ferroptosis	Preclinical (M; A)	Yes—direct competitor at ARE	[131,132]
T3; Glycolysis (downstream of BACH1)	MCT1 inhibition	AZD3965	Block BACH1-driven metastasis	*KEAP1*-mutant lung cancer metastasis	Phase I (M; EC)	Yes—direct competitor at ARE	[22]
T3; Glutaminase (downstream of NRF2)	Glutaminase inhibition	Telaglenastat (CB-839)	Exploit metabolic dependency in NRF2-active tumors	*KEAP1*/*NFE2L2*-mutant NSCLC	Phase II (KEAPSAKE terminated; BeGIN active/closed to accrual) (M; A; CT)	Downstream (glutaminase inhibitor)	[67]
T3; mTORC1/2 (downstream of NRF2)	Dual mTORC1/2 inhibition	Sapanisertib	Exploit mTOR dependency	*KEAP1*/*NFE2L2*-mutant NSCLC	Phase II (M; EC)	Inferred—functional evidence	[68]
T4; Direct NRF2/mRNA	siRNA-LNP; PROTAC against NRF2; brusatol (translational inhibitor)	siRNA-LNP; brusatol	Suppress NRF2 in mutant cancer	*KEAP1*-mutant NSCLC	Preclinical (M)	Inferred—functional evidence	[60]

Evidence origin for each therapeutic concept is graded as: (M) mechanistic studies in cell systems only; (A) animal models with disease-relevant readouts; (EC) early clinical observations or single-arm studies; (CT) established clinical trials with controlled outcomes. The label reflects evidence for the specific NRF2 partner-code mechanism, not the general clinical familiarity of the compound: for example, cyclosporin A (M; CT for its established immunosuppressant use, but no NRF2-targeted trial), lithium (M; A), PRMT inhibitors such as GSK3368715 (M; EC in unrelated oncology indications), IQGAP1-directed approaches (M only; not currently druggable), and MED16-directed interventions (M only; hypothesis-generating). Clinical-stage agents are further sub-stratified in Table 5 as Tier 3a (active), 3b (terminated), and 3c (negative outcome in unstratified populations). FDA-approved drugs are marked ‡. Tier classification (prefix in first column): T1 = approved NRF2-pathway therapeutics; T2 = approved drugs with partner-code repositioning potential (not approved for NRF2 indication); T3 = clinical-stage partner-adjacent agents; T4 = preclinical or conceptual interfaces. Combined tiers (e.g., T2/4) indicate that distinct compounds at different maturities target the same interface.

Scope note. The drug repositioning concepts developed in this section are biomarker-driven mechanistic hypotheses, not clinical recommendations. Each candidate requires direct demonstration of NRF2 partner engagement at therapeutic exposures in disease-relevant models, biomarker-stratified clinical evaluation, and assessment of indication-specific safety liabilities (e.g., immunosuppression with cyclosporin A, narrow therapeutic window with lithium, calcineurin-dependent off-target effects, on-target hematologic toxicities with ATRA/arsenic trioxide). The tiering in Table 4 reflects mechanistic plausibility and pharmacological accessibility, not regulatory readiness. The added “NRF2 partner engagement” column distinguishes interfaces where partner-NRF2 interaction has been directly validated from those inferred from class-level pharmacology or downstream functional readouts.

To avoid conflating biological attractiveness with clinical readiness, we use four tiers. Tier 1 comprises approved NRF2-pathway therapeutics whose indications are directly linked to NRF2 pathway activation: currently, dimethyl fumarate for multiple sclerosis and omaveloxolone for Friedreich ataxia. Tier 2 comprises approved drugs with partner-code repositioning potential, such as cyclosporin A, lithium, all-trans retinoic acid, and arsenic trioxide. Tier 3 comprises partner-adjacent agents that have reached clinical evaluation and are sub-stratified to distinguish current clinical readiness (Supplementary Table S3 catalogs the registered clinical trials underlying this sub-stratification). Tier 3a (active clinical-stage) includes CCS1477/inobrodib, which is currently in Phase I/II trials for hematologic malignancies and selected solid tumors. Tier 3b (completed or terminated with explicit negative or limited clinical outcome) includes telaglenastat (KEAPSAKE Phase II terminated for lack of clinical benefit; BeGIN Phase II completed), bardoxolone methyl (BEACON Phase III terminated for excess cardiovascular events; subsequent ADPKD and PAH programs also terminated), sapanisertib (Phase II completed in *KEAP1*-mutant NSCLC), and GSK3368715 (Phase I terminated). Tier 3c (historical clinical-stage with negative or limited evidence in unstratified populations) includes tideglusib (Phase II completed in progressive supranuclear palsy without significant benefit) and apatorsen (Phase II completed/terminated in castration-resistant prostate cancer and NSCLC). This sub-stratification makes explicit that Tier 3 exposure does not imply clinical readiness; several of these agents require biomarker-stratified re-evaluation rather than direct repositioning. Tier 4 comprises preclinical or conceptual interfaces such as *MED16* disruption, WDR23/CRL4 modulation, IQGAP1 peptidomimetics, and Hrd1 inhibition. This tiering clarifies what the framework supports now and what remains speculative.

The approximately 25 interfaces enumerated in Table 4 are counted by distinct molecular contact site rather than by drug compound: KEAP1 Kelch-pocket PPI inhibitors (e.g., KI-696) and KEAP1 Cys151 covalent modifiers (e.g., omaveloxolone) are counted as separate interfaces, whereas multiple GSK-3β inhibitors targeting the same β-TrCP-Neh6 phosphodegron axis are counted as one. Supplementary Table S4 enumerates each interface and its developmental stage.

### 7.1. The Case for Partner-Selective NRF2 Modulation

For three decades, NRF2 pharmacology has been dominated by attempts to activate NRF2 by disabling KEAP1. This strategy has produced approved therapies in specific indications, including dimethyl fumarate for multiple sclerosis and omaveloxolone for Friedreich ataxia, but it has also exposed the limitations of broad NRF2 activation. The bardoxolone methyl BEACON trial was terminated early because of excess cardiovascular events, emphasizing that systemic NRF2 activation can have tissue- and context-dependent liabilities. Broad activation is therefore not a universal solution.

The partner-code framework suggests a more selective alternative: identify and modulate the partner that is rate-limiting in the disease of interest. In Friedreich ataxia, KEAP1-linked activation is therapeutically useful. In neurodegeneration, the relevant axis may be GSK-3β–β-TrCP. In *KEAP1*-mutant NSCLC, KEAP1 is already disabled, so tractability shifts to PPIA, PIN1, HSP27, RAC3, CBP/p300, and downstream metabolic dependencies. In chronic liver disease, the appropriate direction of intervention may change across disease stages. The therapeutic aim is not simply to increase or decrease NRF2 but to correct the disease-specific partner configuration.

### 7.2. Druggable Interfaces in the Degradation Module

KEAP1 Cys151 covalent modifiers comprise the largest class. Sulforaphane is being evaluated in clinical studies for cancer prevention and neurobehavioral indications. Dimethyl fumarate is approved for relapsing-remitting multiple sclerosis and psoriasis [145,146]. Bardoxolone methyl advanced into late-stage programs for autosomal dominant polycystic kidney disease and pulmonary arterial hypertension but has faced safety-related limitations. Omaveloxolone is approved for Friedreich ataxia [147,148]. These agents validate KEAP1 as a druggable node while also illustrating the need for careful tissue-specific risk assessment.

Non-covalent KEAP1-NRF2 PPI inhibitors were developed to avoid the off-target reactivity of cysteine modifiers. ML334 (LH601A) was the first reported direct PPI inhibitor with Kd = 1.0 μM [160]. KI-696 is a fragment-based discovery probe with low nanomolar cellular potency [159], but its low oral bioavailability has limited its use to research. Newer chemotypes are at preclinical stages with improved drug-like properties [159].

The β-TrCP/Neh6 axis can be modulated indirectly through GSK-3β inhibitors. Lithium has long clinical experience in bipolar disorder, whereas tideglusib has been tested in neurodegenerative and neuromuscular indications. Both are repositioning candidates rather than established NRF2 therapies. For the GSK-3β/β-TrCP–NRF2 mechanism, the supporting evidence is mechanistic and from animal models (M; A); lithium’s established clinical use in bipolar disorder does not constitute NRF2-targeted evidence. Hrd1 and WDR23 axes remain preclinical: Hrd1 inhibitors have shown activity in ER-stress-driven liver and renal models [14,36], and WDR23–NRF2 disruption strategies are still at tool-compound or concept stages.

### 7.3. Pharmacologically Addressable Interfaces in the Cytoplasmic Scaffold Module

This module is among the more pharmacologically accessible layers of the partner code, with the important caveat that none of the relevant agents are approved for an NRF2 pathway indication. Several long-standing clinical drugs intersect the module mechanistically, but they should be considered sources of testable repositioning hypotheses rather than direct off-label recommendations.

PPIA–NRF2 interface (Tier 2—repositioning hypothesis): cyclosporin A, an approved immunosuppressant, disrupts PPIA–NRF2 binding at trans-Pro174 [90]. In NRF2-hyperactive NSCLC patient-derived xenograft models, cyclosporin A promoted NRF2 ubiquitination, suppressed glutamine-driven metabolism through KLF5–SLC1A5, and slowed tumor progression. Combination with glutaminase inhibition enhanced antitumor effects [90]. These data justify a testable repositioning hypothesis for *KEAP1*/*NFE2L2*-mutant NSCLC, but immunosuppression, calcineurin dependence, drug–drug interactions, and therapeutic-window constraints require careful evaluation. The NRF2-relevant mechanism rests on cell-system evidence only (M); although cyclosporin A has an established clinical safety profile as an immunosuppressant (CT), no clinical study has tested it for NRF2 modulation, so the (CT) evidence does not extend to the partner-code mechanism.

PIN1-NRF2 interface: KPT-6566 [107] is a covalent inhibitor with a dual mechanism—covalent binding to PIN1 Cys113 (IC_50_ = 640 nM) plus release of a quinone moiety generating ROS and DNA damage. Sulfopin [108] is the most selective PIN1 inhibitor—covalent binding to Cys113 with double-digit nanomolar potency, validated by chemoproteomics, with negligible off-target toxicity and demonstrated efficacy in *MYCN*-driven neuroblastoma and pancreatic cancer mouse models. All-trans retinoic acid and arsenic trioxide also inhibit PIN1 (to a lesser extent) and remain in clinical use for acute promyelocytic leukemia.

HSP27-NRF2 interface (Tier 3—clinical-stage): OGX-427 (apatorsen), an antisense oligonucleotide targeting HSP27 mRNA, has reached Phase II testing in castration-resistant prostate cancer and NSCLC, with several of those trials now completed or terminated rather than ongoing [86,163]. The Chen et al. finding [85] that PIPKIγ-PI(4,5)P_2_-HSP27 stabilizes NRF2 suggests that part of apatorsen’s antitumor activity may be mediated by NRF2 destabilization, although a dedicated re-analysis of trial samples for NRF2-target gene engagement would be required to test this mechanism directly. p62-KEAP1 interface (Tier 3/4): TBK1 inhibitors (BX795, GSK8612, momelotinib in part) reduce phospho-p62 S349 and decrease KEAP1 sequestration, providing an indirect route to suppress NRF2 in autophagy-deficient HCC [44,99]; momelotinib is FDA-approved for myelofibrosis but its NRF2-related repositioning remains conceptual. IQGAP1-NRF2 interface (Tier 4—preclinical/conceptual): peptidomimetics targeting the IQ domain are conceptually feasible but not yet validated (evidence origin: M only; not currently druggable); indirect modulation through Ca^2+^ channel inhibitors and MEK inhibitors provides exploratory translational handles in TRPM2-IQGAP1-driven chemoresistant tumors [82].

### 7.4. Druggable Interfaces in the Nuclear Coactivator and Chromatin Module

The nuclear module is the richest and least exploited layer for partner-selective NRF2 inhibition. Because *KEAP1*/*NFE2L2*-mutant cancers depend critically on the nuclear coactivator complex assembled at Neh4/5, and because each component is independently druggable, this module provides the most direct route to suppress oncogenic NRF2 in genotype-stratified populations.

CBP/p300–NRF2 (Neh4/5; Tier 3 clinical-stage): A-485 is a selective p300 histone acetyltransferase inhibitor with preclinical activity in *KEAP1*-mutant NSCLC. CCS1477 (inobrodib) is a CBP/p300 inhibitor in Phase I/II studies for hematological malignancies and selected solid tumors (NCT04068597). A 2025 study by the Wang/Yamamoto group [161] reported that CCS1477 represses NRF2-dependent cytoprotective transcription and resensitizes cancer cells to chemotherapy, making it one of the more advanced nuclear coactivator strategies in the partner-code map. The key concept is output suppression: these compounds leave NRF2 protein levels intact while reducing transcriptional activity at target enhancers.

RAC3/SRC-3-NRF2 (Neh4/5)—Tier 4 preclinical: Bufalin [162] was the first p160 SMI identified by HTS, with low nanomolar potency, but cardiac glycoside activity limited development; the 3-phospho-bufalin (p-Buf) prodrug [164] addresses cardiotoxicity. SI-2 [165] is more drug-like, with an IC50 in the low nM range; fluorinated SI-2 analogs with improved plasma half-life have shown efficacy against breast cancer lung metastasis. Verrucarin A and gossypol are additional first-generation SRC-3 SMIs. We propose that these compounds may suppress oncogenic NRF2 in *NCOA3*-amplified or *KEAP1*-mutant tumors, although the direct link between NCOA3 amplification and elevated NRF2 target output in human tumor specimens remains to be quantified—this is a hypothesis the framework generates rather than a finding it summarizes.

PRMT1-NRF2 (Neh4/5 synergy): GSK3368715 is a type I PRMT inhibitor in Phase I/II for AML, multiple myeloma, and select solid tumors (NCT03666988). EZM2302 is a CARM1 inhibitor at preclinical stages. For the partner-code mechanism, the evidence is mechanistic, with early clinical experience in unrelated oncology indications (M; EC). Current trials target oncology contexts unrelated to NRF2 biology, so the NRF2-selective effect remains to be directly tested.

BACH1-small Maf axis: HPPE [166], cannabidiol quinones with dual BACH1-inhibitor/NRF2-activator activity [167], and HO-1 inhibitors (zinc protoporphyrin, tin protoporphyrin) indirectly suppress BACH1 stabilization in *KEAP1*-mutant tumors. The MCT1 inhibitor AZD3965, in Phase I trials, exploits the metabolic vulnerability created by BACH1-driven glycolysis [23].

Mediator/MED16 axis (Tier 4—conceptual): (evidence origin: M only; hypothesis-generating) the finding by Sekine et al. that *MED16* disruption attenuates approximately 75% of NRF2 target-gene induction without reducing nuclear NRF2 levels makes MED16 one of the most informative nodes in the partner code [16]. However, biological importance should not be conflated with clinical druggability. The Mediator complex has broad roles in transcription, creating a high risk of pleiotropic toxicity, and no MED16-selective compound has been reported. MED16-directed degradation or selective disruption of the MED16–MED23/MED24 tail submodule is therefore a long-term research concept rather than a near-term therapeutic strategy.

### 7.5. Combination and Biomarker-Stratified Strategies

The framework supports three combination logics. First, dual-axis modulation within the same module may be useful when two degradation pathways constrain NRF2 simultaneously; for example, GSK-3β inhibition plus KEAP1 modulation could target both β-TrCP/Neh6 and KEAP1/Neh2 brakes in selected neurodegenerative contexts [69,144]. Second, partner-selective inhibition in mutant-driven cancer may combine a coactivator or scaffold inhibitor with metabolic dependencies created by NRF2 hyperactivation. Third, stage-specific bidirectional modulation may be required in diseases such as chronic liver disease, where NRF2 can be protective early but tumor-promoting later.

Clinical translation should therefore be disease- and genotype-specific. Patient-level molecular characterization—including KEAP1/NFE2L2 mutation status, BACH1 levels, p62 accumulation, HSP27/PIN1/PPIA expression, coactivator dependency, and GSK-3β activity—should guide selection of the partner to modulate and the direction of modulation. Pharmacodynamic readouts should go beyond total NRF2 protein to include target-gene induction, chromatin occupancy, coactivator recruitment, and metabolic flux (see also Supplementary Figure S1 for a 10-disease × 9-therapy-class stratification grid).

Table 5 operationalizes the framework’s prescription that disease context and biomarker-driven module selection should guide the NRF2 modulation strategy. The matrix is a framework-derived lookup tool, not a clinical decision algorithm; all entries require direct NRF2 partner-engagement validation and indication-specific safety assessment before clinical application.

**Table 5:** Partner-code decision matrix: disease-genotype-biomarker context, recommended modulation direction, candidate partner targets, and evidence level.

Disease/Genotype/Biomarker Context	Modulation Direction	Candidate Partner Targets (Compound/Status)	Evidence Level	Refs.
*KEAP1*/*NFE2L2*-mutant NSCLC (no NCOA3-amp, no PIN1-high)	Suppress NRF2 output	PPIA (CsA repositioning); glutaminase downstream (telaglenastat—Tier 3b); SLC7A11 (sulfasalazine)	Strong (KEAP1-mut NSCLC biology); Moderate (CsA-NRF2 link); Limited (telaglenastat—terminated)	[90]
KEAP1-mut + *NCOA3*-amplified NSCLC	Suppress NRF2 output	CBP/p300 (CCS1477—Tier 3a); RAC3/SRC-3 (SI-2, preclinical)	Moderate (coactivator dependency); Active trial (CCS1477)	[161]
KEAP1-mut + PIN1-high NSCLC	Suppress NRF2 output	PIN1 (ATRA, ATO—approved for APL; sulfopin—preclinical)	Strong (PIN1 inhibition in APL); Inferred (NSCLC repositioning hypothesis)	[87,106]
KEAP1-mut + p62-aggregated NSCLC	Suppress p62-driven NRF2 hyperactivation	TBK1 (BX795; off-label kinase inhibitors); p62-KIR (preclinical)	Moderate (TBK1-p62 mechanistic); Limited (no NRF2-specific drug)	[99]
KEAP1-WT + p62-aggregated HCC (NASH-driven)	Suppress p62-driven NRF2 hyperactivation	p62 KIR disruptor (conceptual); restore autophagy	Strong (Komatsu p62-HCC biology); No chemical lead	[42]
KEAP1-WT + Hrd1-induced cirrhosis	Enhance NRF2 output (rescue suppressed cytoprotection)	Hrd1 inhibitor (LS-102—preclinical); IRE1α-XBP1 axis modulators	Moderate (Wu 2014); Preclinical only	[14]
KEAP1-WT + GSK-3β-hyperactive AD (pTau biomarker positive)	Enhance NRF2 output (rescue β-TrCP-driven degradation)	GSK-3β (lithium—approved; tideglusib—Tier 3c, biomarker stratification proposed)	Moderate (GSK-3β in AD); Tideglusib PSP Phase II negative—requires biomarker stratification	[69,142]
KEAP1-WT + AKT-driven T2D/metabolic syndrome	Enhance NRF2 output (exploratory)	GSK-3β (lithium—approved); insulin sensitizers indirectly	Limited (mechanistic hypothesis; no T2D-specific NRF2 trial)	[13]
KEAP1-WT + heme-depleted ferroptosis-prone tissue	Enhance NRF2 output via BACH1 destabilization	BACH1 (hemin—approved for porphyria); HPPE (preclinical); HO-1 inhibitors	Moderate (BACH1 biology); Hemin approved for related indication	[131]
Friedreich ataxia (frataxin-deficient)	Enhance NRF2 output (broad activation)	KEAP1 (omaveloxolone—Tier 1, FDA approved 2023)	Strong (MOXIe Phase II; FDA approval)	[147]

### 7.6. Reading the Therapeutic Landscape

The partner-code pharmacopeia (Table 4) identifies approximately 25 addressable interfaces across the four modules. Two agents are Tier 1 approved NRF2-pathway therapeutics (omaveloxolone and dimethyl fumarate). Several approved drugs fall into Tier 2 repositioning hypotheses (cyclosporin A, lithium, all-trans retinoic acid, arsenic trioxide). Clinical-stage partner-adjacent agents form Tier 3 (CCS1477, GSK3368715, telaglenastat, sapanisertib, tideglusib, apatorsen, and bardoxolone methyl). Tier 4 contains mechanistically attractive but immature interfaces such as MED16, WDR23, IQGAP1, Hrd1, and direct NRF2 degradation. The translational message is intentionally conservative: partner selectivity creates opportunities, but the evidence tier must determine the pace of clinical development.

### 7.7. Limitations of the Partner-Code Framework

Several limitations of the partner-code framework, beyond those already noted in individual sections, deserve explicit acknowledgment. First, this is a structured narrative review and not a systematic review; while inclusion criteria were stated (Section 1.4) and the 22 partners and approximately 25 interfaces are enumerated in Supplementary Tables S1 and S4, author judgement enters at the level of which boundary cases (e.g., DPP3, NBR1, WTX) to exclude and how to assign bridging partners (p62, PIN1) to a primary module. Different reviewers might arrive at slightly different partner inventories.

Second, the framework conflates direct NRF2 partners (those that bind NRF2 itself, such as KEAP1, PPIA, and MED16) with indirect modulators (those that bind KEAP1 or affect NRF2 turnover without direct NRF2 contact, such as p62/SQSTM1 via the KEAP1 KIR pocket or DPP3 via the KEAP1 Kelch domain). Although Table S3 distinguishes these categories with a “Direct NRF2?” column, the four-module organization itself does not always make this distinction visually transparent. The framework should be interpreted as a regulatory architecture of NRF2 output, not as a pure structural classification of NRF2-binding proteins.

Third, clinical trial status is intrinsically volatile. The therapeutic tiering reflects ClinicalTrials.gov entries as of January 2026; several Tier 3 trials (KEAPSAKE telaglenastat, BEACON bardoxolone, GSK3368715) have already been terminated, and others may be updated before publication. The sub-stratification of Tier 3 into 3a, 3b, and 3c (Section 7) is intended to make this status volatility transparent, but readers should re-verify trial status before drawing conclusions about repositioning.

Fourth, disease-model evidence is heterogeneous across species, model systems, and pharmacodynamic readouts. WDR23 evidence draws on *C. elegans* WDR-23 plus mammalian DDB1-CUL4 data; β-TrCP/Neh6 evidence in neurodegeneration relies on mouse and cell-based models without direct human PD biomarker validation; HSP27 evidence in NRF2 partner biology comes largely from cancer cell lines. The framework should be retested in tissue- and species-matched systems before high-confidence translational claims.

Fifth, the partner-code framework should not be read as a complete inventory of NRF2 biology. Stress-sensing post-translational modifications on NRF2 itself (phosphorylation, acetylation, SUMOylation), non-canonical NRF2 functions outside transcription, and crosstalk with parallel CNC-bZIP factors (NRF1, NRF3, BACH2) are not exhaustively treated here. Downstream applications of the framework should explicitly state which extensions or adaptations they make.

Finally, because clinical trial information evolves rapidly, the trial statuses, registry identifiers, and developmental stages cited in §7 and in Supplementary Table S4 reflect the state of ClinicalTrials.gov and the relevant regulatory databases as of the manuscript submission date (January 2026). We will re-verify all trial statuses at the proof stage, and readers consulting this review at later time points should treat these annotations as time-stamped snapshots rather than the current status.

## 8. Future Directions: Five Testable Hypotheses

The partner-code framework developed in Section 2, Section 3, Section 4, Section 5, Section 6 and Section 7 generates five testable hypotheses that operate at different levels of resolution. Hypothesis 1 expands the structural map by treating interdomain linkers as regulatory territory. Hypothesis 2 predicts that individual post-translational modification states can act as binary partner switches. Hypothesis 3 proposes that transcriptional amplitude is often limited by coactivator assembly rather than by NRF2 stabilization. Hypothesis 4 evaluates whether approved partner-selective drugs can be repositioned in biomarker-defined populations. Hypothesis 5 addresses the central clinical challenge: the same partner may need to be activated in one disease and inhibited in another.

### 8.1. Hypothesis 1: Interdomain Linkers Are a Distinct Stratum of the Partner Code

The disordered linkers between Neh domains may host druggable partner interactions. Two findings motivate this hypothesis: the PPIA–NRF2 interaction at trans-Pro174 in the Neh4–Neh5 linker [90] and the PIN1 phospho-recognition site at S408 in the Neh6–Neh1 linker [88]. Test: combine AlphaFold-Multimer predictions, disorder mapping, proximity-labeling proteomics, and residue-resolved mutagenesis to prioritize and validate linker-binding partners. Success criteria should include concordant structural prediction, orthogonal biochemical binding, cellular NRF2 pharmacodynamics, and functional target-gene effects.

### 8.2. Hypothesis 2: Single-Residue Partner Switching Is a General Mechanism

The S215 example (Section 4.4)—phospho-S215 favoring PIN1-linked stabilization and unphosphorylated S215 favoring RXRα-linked repression—illustrates a general principle. Candidate switch residues include S408, which may coordinate β-TrCP and PIN1 logic, and K443 in Neh1, where acetylation and SUMO-related regulation may compete. Test: use phospho-null, phospho-mimetic, acetylation-null, and acetylation-mimetic substitutions with quantitative interactomics and reporter assays. A residue would qualify as a partner switch if reciprocal changes in competing partner binding are coupled to opposite NRF2-output phenotypes.

### 8.3. Hypothesis 3: NRF2 Transcriptional Amplitude Is Rate-Limited by Coactivator Complex Assembly

The Sekine et al. [16] study provides strong evidence that coactivator assembly can be rate-limiting: MED16 deficiency attenuated approximately 75% of NRF2 target-gene induction without reducing nuclear NRF2 levels. If this principle generalizes, a substantial fraction of NRF2 dysfunction in disease should be interpreted as a coactivator-assembly problem rather than a stability problem. Test: perform NRF2, RAC3, MED16, and p300 ChIP-seq or CUT&Tag in matched healthy and diseased tissues, integrate these data with single-cell RNA-seq, and determine whether output tracks coactivator occupancy more closely than NRF2 abundance.

### 8.4. Hypothesis 4: Approved Partner-Selective Compounds Can Be Repositioned in Biomarker-Defined NRF2 Contexts

Section 6 and Section 7 identified approved or clinically advanced drugs whose mechanisms overlap with the partner code. We propose that some will be most useful in biomarker-defined populations rather than in unselected NRF2-associated diseases. Strong candidates include cyclosporin A in *KEAP1*/*NFE2L2*-mutant NSCLC with PPIA–NRF2 dependence; lithium or tideglusib in neurodegenerative contexts with GSK-3β/β-TrCP-driven NRF2 suppression; and all-trans retinoic acid or arsenic trioxide in tumors with PIN1-dependent NRF2 stabilization. Prospective testing should require partner-engagement biomarkers and prespecified NRF2 target-gene endpoints.

### 8.5. Hypothesis 5: The Same Partner Can Require Activation in One Disease and Inhibition in Another

This hypothesis captures the translational risk of NRF2 biology. NRF2 activation can suppress primary tumorigenesis in some contexts but promote lung cancer metastasis by stabilizing BACH1 [22,23]. p62 accumulation can drive HCC oncogenesis [44] while protecting cells from ferroptosis. BACH1 represses ferroptosis-protective genes but can also shape pro-metastatic metabolic programs. Test: conduct a PRISMA-compliant systematic review and meta-analysis of primary mechanistic studies, stratified by disease, cell type, partner, direction of perturbation, and outcome. The goal would be to identify where activation, inhibition, or bidirectional stage-specific modulation is justified.

### 8.6. The Five Hypotheses as a Research Program

Together, the five hypotheses define a practical research program. H1 expands the structural map. H2 resolves partner control at single-residue precision. H3 shifts attention from NRF2 stability to transcriptional assembly. H4 translates the framework into biomarker-driven repositioning. H5 prevents oversimplified therapeutic interpretation by requiring disease-specific directionality. Each hypothesis is testable with current structural, proteomic, genomic, and pharmacological tools.

The next phase of NRF2 research is likely to be shaped less by the search for universal activators or inhibitors and more by systematic mapping of partner combinations across cell states, disease contexts, and therapeutic interventions. The partner-code framework provides a scaffold for that mapping. Its value will depend on rigorous validation: partner engagement must be measured, disease context must be specified, and claims of therapeutic readiness must remain proportional to the evidence tier.

### 8.7. The Partner Code Beyond NRF2: Relevance to Other CNC-bZIP Family Members

The framework developed in this review is centered on NRF2 because its partner repertoire is best documented and because NRF2 is the principal pharmacological target within the CNC-bZIP family. Whether the partner-code logic generalizes to NRF1 (NFE2L1), NRF3 (NFE2L3), and BACH2 is an empirical question and an immediate extension of the framework. NRF1 shares the obligate small-Maf heterodimerization step and the ARE consensus, but it is membrane-anchored at the ER and is regulated primarily by Hrd1/SYVN1 and by proteasomal feedback rather than by KEAP1, suggesting a re-weighted degradation module and a partly shared DNA/chromatin module. NRF3 has a similar membrane-anchored architecture and emerging roles in oncogenic transcription, again pointing to a degradation module that is dominated by proteostasis rather than redox sensors. BACH2 belongs to the BACH subfamily, which competes with NRF2 at shared AREs and is itself regulated by heme; it is therefore most naturally read as a DNA/chromatin-module repressor whose stoichiometry relative to NRF2 is biologically meaningful in lymphocyte differentiation. A useful next step would be to apply the four-module template to each of these factors—asking, for each, which module is rate-limiting, which partners overlap with the NRF2 set, and whether any of the partner-selective therapeutic strategies considered here have plausible specificity. The framework does not claim to predict the answers in advance, but it provides a vocabulary for posing the question consistently across the family.

## 9. Conclusions

The partner-code framework does not replace the KEAP1 model; it embeds the canonical axis within a broader architecture in which degradation, scaffolding, coactivator assembly, and chromatin competition jointly determine NRF2 output. Rather than summarizing the framework, we close by extracting four take-home rules that we believe should guide downstream applications and clinical translation.

Rule 1. NRF2 abundance is not a sufficient biomarker. Transcriptional output is also limited by coactivator assembly, competition with BACH1 for DNA/chromatin binding, and disease-specific partner rewiring. Pharmacodynamic readouts should include NRF2 target-gene programs (Supplementary Table S5), not only NRF2 protein levels.

Rule 2. The same partner can require opposite modulation in different diseases. BACH1 inhibition is protective in ferroptosis-prone neurodegeneration but should be exploited (not relieved) in *KEAP1*-mutant lung cancer, where BACH1 stabilization drives metastasis. The partner-code decision matrix (Table 5) operationalizes this directionality across ten disease-genotype combinations.

Rule 3. Clinical-stage exposure is not clinical readiness. Several Tier 3 agents (telaglenastat in KEAPSAKE, bardoxolone in BEACON, sapanisertib, GSK3368715) have already terminated; others have completed without significant benefit (tideglusib, apatorsen). The Tier 3a/3b/3c sub-stratification makes this transparent. Repositioning should follow biomarker-stratified re-evaluation, not direct extrapolation from approved indications.

Rule 4. Match evidence tier to claim strength. Direct biochemical (structural, mutagenesis) evidence supports strong claims; co-IP and interactome data support qualified claims; class-level pharmacological inference supports hypothesis-generating claims only. Supplementary Table S2 marks every partner with its evidence type, and the “NRF2 partner engagement” column of Table 4 marks every drug with whether its NRF2 interaction is directly validated or inferred.

If these four rules are observed, the partner-code framework can serve as both a synthesis tool for NRF2 biology and a discipline tool for NRF2 pharmacology. The immediate priority is experimental discipline: define the rate-limiting partner in each context, verify direct target engagement at therapeutic exposures, stratify by disease genotype and cell state, and keep therapeutic claims aligned with the evidence tier on which they rest.

## Data Availability

No new data were created or analyzed in this study. Data sharing is not applicable to this article.

## Supplementary Materials

The following supporting information can be downloaded at: https://www.mdpi.com/article/10.3390/antiox15060759/s1, S1: Glossary of partner-code framework terms; Table S1: The 22 partners included in the partner-code atlas and their inclusion criteria; Table S2: Partner summary matrix listing module assignment, binding evidence level, therapeutic tier, and disease-context directionality; Table S3: Clinical trials targeting NRF2 partner-code interfaces; Table S4: The approximately 25 pharmacologically addressable interfaces and their developmental stage; Table S5: NRF2 target gene programs by partner combination; Figure S1: Disease-genotype-cofactor stratification grid.

## Abbreviations

The following abbreviations are used in this manuscript:

AD	Alzheimer’s disease
ADPKD	autosomal dominant polycystic kidney disease
AKT	protein kinase B
ALS	amyotrophic lateral sclerosis
AML	acute myeloid leukemia
ARE	antioxidant response element
ATRA	all-trans retinoic acid
BACH1	BTB and CNC homology 1
BTB	broad-complex/tramtrack/bric-à-brac
bZIP	basic-leucine zipper
CBP	CREB-binding protein
CHD6	chromodomain helicase DNA-binding protein 6
CKD	chronic kidney disease
CNC	cap’n’collar
CRL3/CRL4	cullin-RING ligase 3/4
CRPC	castration-resistant prostate cancer
CsA	cyclosporin A
DCAF11	DDB1- and CUL4-associated factor 11
DMF	dimethyl fumarate
ER	endoplasmic reticulum
ETGE	glutamate-threonine-glycine-glutamate motif
FA	Friedreich ataxia
FDA	U.S. Food and Drug Administration
FSP1	ferroptosis suppressor protein 1
FTH1	ferritin heavy chain 1
GCLC	glutamate-cysteine ligase catalytic subunit
GPX4	glutathione peroxidase 4
GSK-3β	glycogen synthase kinase 3β
HAT	histone acetyltransferase
HCC	hepatocellular carcinoma
HMOX1/HO-1	heme oxygenase 1
HNSCC	head and neck squamous cell carcinoma
HSP27	heat shock protein 27
IQGAP1	IQ motif-containing GTPase activating protein 1
KEAP1	Kelch-like ECH-associated protein 1
KIR	KEAP1-interacting region
KLF5	Krüppel-like factor 5
MAPK	mitogen-activated protein kinase
MED16	mediator complex subunit 16
MS	multiple sclerosis
NASH	non-alcoholic steatohepatitis
NCOA3	nuclear receptor coactivator 3
Neh	NRF2-ECH homology (domain)
*NFE2L2*	nuclear factor erythroid 2-related factor 2 (gene name for NRF2)
NQO1	NAD(P)H quinone dehydrogenase 1
NRF2	nuclear factor erythroid 2-related factor 2
NSCLC	non-small-cell lung cancer
PAH	pulmonary arterial hypertension
PDX	patient-derived xenograft
PI3K	phosphoinositide 3-kinase
PIN1	peptidyl-prolyl cis-trans isomerase NIMA-interacting 1
PIPKIγ	phosphatidylinositol-4-phosphate 5-kinase type 1 gamma
PPI	protein–protein interaction
PPIA	peptidyl-prolyl isomerase A (cyclophilin A)
PRISMA	Preferred Reporting Items for Systematic Reviews and Meta-Analyses
PRMT1	protein arginine methyltransferase 1
PROTAC	proteolysis targeting chimera
PTM	post-translational modification
RAC3	receptor-associated coactivator 3 (=SRC-3 = NCOA3)
ROS	reactive oxygen species
SCF	Skp1-Cullin-F-box
SLC1A5/ASCT2	solute carrier family 1 member 5
SLC7A11	solute carrier family 7 member 11 (xCT)
SQSTM1	sequestosome 1 (=p62)
SRC-3	steroid receptor coactivator 3
SYVN1	synoviolin 1 (=Hrd1)
T2D	type 2 diabetes
TBK1	TANK-binding kinase 1
TCGA	The Cancer Genome Atlas
WDR23	WD repeat-containing protein 23

## References

[1] Moi P., Chan K., Asunis I., Cao A., Kan Y.W. (1994). Isolation of NF-E2-related factor 2 (Nrf2), a NF-E2-like basic leucine zipper transcriptional activator that binds to the tandem NF-E2/AP1 repeat of the beta-globin locus control region. Proc. Natl. Acad. Sci. USA.

[2] Itoh K., Wakabayashi N., Katoh Y., Ishii T., Igarashi K., Engel J.D., Yamamoto M. (1999). Keap1 represses nuclear activation of antioxidant responsive elements by Nrf2 through binding to the amino-terminal Neh2 domain. Genes Dev..

[3] Chan K., Lu R., Chang J.C., Kan Y.W. (1996). NRF2, a member of the NFE2 family of transcription factors, is not essential for murine erythropoiesis, growth, and development. Proc. Natl. Acad. Sci. USA.

[4] Wakabayashi N., Itoh K., Wakabayashi J., Motohashi H., Noda S., Takahashi S., Imakado S., Kotsuji T., Otsuka F., Roop D.R. (2003). Keap1-null mutation leads to postnatal lethality due to constitutive Nrf2 activation. Nat. Genet..

[5] Dinkova-Kostova A.T., Holtzclaw W.D., Kensler T.W. (2005). The role of Keap1 in cellular protective responses. Chem. Res. Toxicol..

[6] Kobayashi M., Li L., Iwamoto N., Nakajima-Takagi Y., Kaneko H., Nakayama Y., Eguchi M., Wada Y., Kumagai Y., Yamamoto M. (2009). The antioxidant defense system Keap1-Nrf2 comprises a multiple sensing mechanism for responding to a wide range of chemical compounds. Mol. Cell. Biol..

[7] Saito R., Suzuki T., Hiramoto K., Asami S., Naganuma E., Suda H., Iso T., Yamamoto H., Morita M., Baird L. (2016). Characterizations of Three Major Cysteine Sensors of Keap1 in Stress Response. Mol. Cell. Biol..

[8] Suzuki T., Yamamoto M. (2017). Stress-sensing mechanisms and the physiological roles of the Keap1-Nrf2 system during cellular stress. J. Biol. Chem..

[9] Huang H.C., Nguyen T., Pickett C.B. (2002). Phosphorylation of Nrf2 at Ser-40 by protein kinase C regulates antioxidant response element-mediated transcription. J. Biol. Chem..

[10] Cuadrado A., Manda G., Hassan A., Alcaraz M.J., Barbas C., Daiber A., Ghezzi P., León R., López M.G., Oliva B. (2018). Transcription Factor NRF2 as a Therapeutic Target for Chronic Diseases: A Systems Medicine Approach. Pharmacol. Rev..

[11] Cuadrado A., Rojo A.I., Wells G., Hayes J.D., Cousin S.P., Rumsey W.L., Attucks O.C., Franklin S., Levonen A.L., Kensler T.W. (2019). Therapeutic targeting of the NRF2 and KEAP1 partnership in chronic diseases. Nat. Rev. Drug Discov..

[12] Chowdhry S., Zhang Y., McMahon M., Sutherland C., Cuadrado A., Hayes J.D. (2013). Nrf2 is controlled by two distinct β-TrCP recognition motifs in its Neh6 domain, one of which can be modulated by GSK-3 activity. Oncogene.

[13] Rada P., Rojo A.I., Chowdhry S., McMahon M., Hayes J.D., Cuadrado A. (2011). SCF/{beta}-TrCP promotes glycogen synthase kinase 3-dependent degradation of the Nrf2 transcription factor in a Keap1-independent manner. Mol. Cell. Biol..

[14] Wu T., Zhao F., Gao B., Tan C., Yagishita N., Nakajima T., Wong P.K., Chapman E., Fang D., Zhang D.D. (2014). Hrd1 suppresses Nrf2-mediated cellular protection during liver cirrhosis. Genes. Dev..

[15] Lo J.Y., Spatola B.N., Curran S.P. (2017). WDR23 regulates NRF2 independently of KEAP1. PLoS Genet..

[16] Sekine H., Okazaki K., Ota N., Shima H., Katoh Y., Suzuki N., Igarashi K., Ito M., Motohashi H., Yamamoto M. (2016). The Mediator Subunit MED16 Transduces NRF2-Activating Signals into Antioxidant Gene Expression. Mol. Cell. Biol..

[17] Motohashi H., Katsuoka F., Engel J.D., Yamamoto M. (2004). Small Maf proteins serve as transcriptional cofactors for keratinocyte differentiation in the Keap1-Nrf2 regulatory pathway. Proc. Natl. Acad. Sci. USA.

[18] Reichard J.F., Motz G.T., Puga A. (2007). Heme oxygenase-1 induction by NRF2 requires inactivation of the transcriptional repressor BACH1. Nucleic Acids Res..

[19] Sun J., Hoshino H., Takaku K., Nakajima O., Muto A., Suzuki H., Tashiro S., Takahashi S., Shibahara S., Alam J. (2002). Hemoprotein Bach1 regulates enhancer availability of heme oxygenase-1 gene. EMBO J..

[20] Kim J.H., Yu S., Chen J.D., Kong A.N. (2013). The nuclear cofactor RAC3/AIB1/SRC-3 enhances Nrf2 signaling by interacting with transactivation domains. Oncogene.

[21] Lin W., Shen G., Yuan X., Jain M.R., Yu S., Zhang A., Chen J.D., Kong A.N. (2006). Regulation of Nrf2 transactivation domain activity by p160 RAC3/SRC3 and other nuclear co-regulators. J. Biochem. Mol. Biol..

[22] Lignitto L., LeBoeuf S.E., Homer H., Jiang S., Askenazi M., Karakousi T.R., Pass H.I., Bhutkar A.J., Tsirigos A., Ueberheide B. (2019). Nrf2 Activation Promotes Lung Cancer Metastasis by Inhibiting the Degradation of Bach1. Cell.

[23] Wiel C., Le Gal K., Ibrahim M.X., Jahangir C.A., Kashif M., Yao H., Ziegler D.V., Xu X., Ghosh T., Mondal T. (2019). BACH1 Stabilization by Antioxidants Stimulates Lung Cancer Metastasis. Cell.

[24] Inami Y., Waguri S., Sakamoto A., Kouno T., Nakada K., Hino O., Watanabe S., Ando J., Iwadate M., Yamamoto M. (2011). Persistent activation of Nrf2 through p62 in hepatocellular carcinoma cells. J. Cell Biol..

[25] Sun X., Ou Z., Chen R., Niu X., Chen D., Kang R., Tang D. (2016). Activation of the p62-Keap1-NRF2 pathway protects against ferroptosis in hepatocellular carcinoma cells. Hepatology.

[26] Sanchez-Vega F., Mina M., Armenia J., Chatila W.K., Luna A., La K.C., Dimitriadoy S., Liu D.L., Kantheti H.S., Saghafinia S. (2018). Oncogenic Signaling Pathways in The Cancer Genome Atlas. Cell.

[27] Nam L.B., Keum Y.S. (2019). Binding partners of NRF2: Functions and regulatory mechanisms. Arch. Biochem. Biophys..

[28] Poh J., Ponsford A.H., Boyd J., Woodsmith J., Stelzl U., Wanker E., Harper N., MacEwan D., Sanderson C.M. (2020). A functionally defined high-density NRF2 interactome reveals new conditional regulators of ARE transactivation. Redox Biol..

[29] Zhang D.D. (2025). Thirty years of NRF2: Advances and therapeutic challenges. Nat. Rev. Drug Discov..

[30] Yamamoto M., Kensler T.W., Motohashi H. (2018). The KEAP1-NRF2 System: A Thiol-Based Sensor-Effector Apparatus for Maintaining Redox Homeostasis. Physiol. Rev..

[31] Tonelli C., Chio I.I.C., Tuveson D.A. (2018). Transcriptional Regulation by Nrf2. Antioxid. Redox Signal..

[32] Robledinos-Antón N., Fernández-Ginés R., Manda G., Cuadrado A. (2019). Activators and Inhibitors of NRF2: A Review of Their Potential for Clinical Development. Oxid. Med. Cell. Longev..

[33] Kobayashi A., Kang M.I., Okawa H., Ohtsuji M., Zenke Y., Chiba T., Igarashi K., Yamamoto M. (2004). Oxidative stress sensor Keap1 functions as an adaptor for Cul3-based E3 ligase to regulate proteasomal degradation of Nrf2. Mol. Cell. Biol..

[34] McMahon M., Thomas N., Itoh K., Yamamoto M., Hayes J.D. (2006). Dimerization of substrate adaptors can facilitate cullin-mediated ubiquitylation of proteins by a “tethering” mechanism: A two-site interaction model for the Nrf2-Keap1 complex. J. Biol. Chem..

[35] Tong K.I., Katoh Y., Kusunoki H., Itoh K., Tanaka T., Yamamoto M. (2006). Keap1 recruits Neh2 through binding to ETGE and DLG motifs: Characterization of the two-site molecular recognition model. Mol. Cell. Biol..

[36] Liu M., Reddy N.M., Higbee E.M., Potteti H.R., Noel S., Racusen L., Kensler T.W., Sporn M.B., Reddy S.P., Rabb H. (2014). The Nrf2 triterpenoid activator, CDDO-imidazolide, protects kidneys from ischemia-reperfusion injury in mice. Kidney Int..

[37] Duangjan C., Irwin R.W., Curran S.P. (2024). Loss of WDR23 proteostasis impacts mitochondrial homeostasis in the mouse brain. Cell Signal..

[38] Siswanto F.M., Oguro A., Arase S., Imaoka S. (2020). WDR23 regulates the expression of Nrf2-driven drug-metabolizing enzymes. Drug Metab. Pharmacokinet..

[39] Spatola B.N., Lo J.Y., Wang B., Curran S.P. (2019). Nuclear and cytoplasmic WDR-23 isoforms mediate differential effects on GEN-1 and SKN-1 substrates. Sci. Rep..

[40] Ichimura Y., Waguri S., Sou Y.S., Kageyama S., Hasegawa J., Ishimura R., Saito T., Yang Y., Kouno T., Fukutomi T. (2013). Phosphorylation of p62 activates the Keap1-Nrf2 pathway during selective autophagy. Mol. Cell.

[41] Jain A., Lamark T., Sjøttem E., Larsen K.B., Awuh J.A., Øvervatn A., McMahon M., Hayes J.D., Johansen T. (2010). p62/SQSTM1 is a target gene for transcription factor NRF2 and creates a positive feedback loop by inducing antioxidant response element-driven gene transcription. J. Biol. Chem..

[42] Komatsu M., Kurokawa H., Waguri S., Taguchi K., Kobayashi A., Ichimura Y., Sou Y.S., Ueno I., Sakamoto A., Tong K.I. (2010). The selective autophagy substrate p62 activates the stress responsive transcription factor Nrf2 through inactivation of Keap1. Nat. Cell Biol..

[43] Sun D., Wu R., Zheng J., Li P., Yu L. (2018). Polyubiquitin chain-induced p62 phase separation drives autophagic cargo segregation. Cell Res..

[44] Umemura A., He F., Taniguchi K., Nakagawa H., Yamachika S., Font-Burgada J., Zhong Z., Subramaniam S., Raghunandan S., Duran A. (2016). p62, Upregulated during Preneoplasia, Induces Hepatocellular Carcinogenesis by Maintaining Survival of Stressed HCC-Initiating Cells. Cancer Cell.

[45] Camp N.D., James R.G., Dawson D.W., Yan F., Davison J.M., Houck S.A., Tang X., Zheng N., Major M.B., Moon R.T. (2012). Wilms tumor gene on X chromosome (WTX) inhibits degradation of NRF2 protein through competitive binding to KEAP1 protein. J. Biol. Chem..

[46] Chen W., Sun Z., Wang X.J., Jiang T., Huang Z., Fang D., Zhang D.D. (2009). Direct interaction between Nrf2 and p21(Cip1/WAF1) upregulates the Nrf2-mediated antioxidant response. Mol. Cell.

[47] Gorrini C., Baniasadi P.S., Harris I.S., Silvester J., Inoue S., Snow B., Joshi P.A., Wakeham A., Molyneux S.D., Martin B. (2013). BRCA1 interacts with Nrf2 to regulate antioxidant signaling and cell survival. J. Exp. Med..

[48] Hast B.E., Goldfarb D., Mulvaney K.M., Hast M.A., Siesser P.F., Yan F., Hayes D.N., Major M.B. (2013). Proteomic analysis of ubiquitin ligase KEAP1 reveals associated proteins that inhibit NRF2 ubiquitination. Cancer Res..

[49] Lu K., Alcivar A.L., Ma J., Foo T.K., Zywea S., Mahdi A., Huo Y., Kensler T.W., Gatza M.L., Xia B. (2017). NRF2 Induction Supporting Breast Cancer Cell Survival Is Enabled by Oxidative Stress-Induced DPP3-KEAP1 Interaction. Cancer Res..

[50] Ma J., Cai H., Wu T., Sobhian B., Huo Y., Alcivar A., Mehta M., Cheung K.L., Ganesan S., Kong A.N. (2012). PALB2 interacts with KEAP1 to promote NRF2 nuclear accumulation and function. Mol. Cell. Biol..

[51] Lo S.C., Li X., Henzl M.T., Beamer L.J., Hannink M. (2006). Structure of the Keap1:Nrf2 interface provides mechanistic insight into Nrf2 signaling. EMBO J..

[52] Padmanabhan B., Tong K.I., Ohta T., Nakamura Y., Scharlock M., Ohtsuji M., Kang M.I., Kobayashi A., Yokoyama S., Yamamoto M. (2006). Structural basis for defects of Keap1 activity provoked by its point mutations in lung cancer. Mol. Cell.

[53] Iso T., Suzuki T., Baird L., Yamamoto M. (2016). Absolute Amounts and Status of the Nrf2-Keap1-Cul3 Complex within Cells. Mol. Cell. Biol..

[54] Sekhar K.R., Rachakonda G., Freeman M.L. (2010). Cysteine-based regulation of the CUL3 adaptor protein Keap1. Toxicol. Appl. Pharmacol..

[55] Zhang D.D., Hannink M. (2003). Distinct cysteine residues in Keap1 are required for Keap1-dependent ubiquitination of Nrf2 and for stabilization of Nrf2 by chemopreventive agents and oxidative stress. Mol. Cell. Biol..

[56] Suzuki T., Muramatsu A., Saito R., Iso T., Shibata T., Kuwata K., Kawaguchi S.I., Iwawaki T., Adachi S., Suda H. (2019). Molecular Mechanism of Cellular Oxidative Stress Sensing by Keap1. Cell Rep..

[57] Singh A., Misra V., Thimmulappa R.K., Lee H., Ames S., Hoque M.O., Herman J.G., Baylin S.B., Sidransky D., Gabrielson E. (2006). Dysfunctional KEAP1-NRF2 interaction in non-small-cell lung cancer. PLoS Med..

[58] Frank R., Scheffler M., Merkelbach-Bruse S., Ihle M.A., Kron A., Rauer M., Ueckeroth F., König K., Michels S., Fischer R. (2018). Clinical and Pathological Characteristics of KEAP1- and NFE2L2-Mutated Non-Small Cell Lung Carcinoma (NSCLC). Clin. Cancer Res..

[59] Kim Y.R., Oh J.E., Kim M.S., Kang M.R., Park S.W., Han J.Y., Eom H.S., Yoo N.J., Lee S.H. (2010). Oncogenic NRF2 mutations in squamous cell carcinomas of oesophagus and skin. J. Pathol..

[60] Singh A., Boldin-Adamsky S., Thimmulappa R.K., Rath S.K., Ashush H., Coulter J., Blackford A., Goodman S.N., Bunz F., Watson W.H. (2008). RNAi-mediated silencing of nuclear factor erythroid-2-related factor 2 gene expression in non-small cell lung cancer inhibits tumor growth and increases efficacy of chemotherapy. Cancer Res..

[61] Solis L.M., Behrens C., Dong W., Suraokar M., Ozburn N.C., Moran C.A., Corvalan A.H., Biswal S., Swisher S.G., Bekele B.N. (2010). Nrf2 and Keap1 abnormalities in non-small cell lung carcinoma and association with clinicopathologic features. Clin. Cancer Res..

[62] Zhang Y., Fan H., Fang S., Wang L., Chen L., Jin Y., Jiang W., Lin Z., Shi Y., Zhan C. (2016). Mutations and expression of the NFE2L2/KEAP1/CUL3 pathway in Chinese patients with lung squamous cell carcinoma. J. Thorac. Dis..

[63] Ohta T., Iijima K., Miyamoto M., Nakahara I., Tanaka H., Ohtsuji M., Suzuki T., Kobayashi A., Yokota J., Sakiyama T. (2008). Loss of Keap1 function activates Nrf2 and provides advantages for lung cancer cell growth. Cancer Res..

[64] Hayes J.D., McMahon M. (2009). NRF2 and KEAP1 mutations: Permanent activation of an adaptive response in cancer. Trends Biochem. Sci..

[65] Kerins M.J., Ooi A. (2018). A catalogue of somatic NRF2 gain-of-function mutations in cancer. Sci. Rep..

[66] Shibata T., Ohta T., Tong K.I., Kokubu A., Odogawa R., Tsuta K., Asamura H., Yamamoto M., Hirohashi S. (2008). Cancer related mutations in NRF2 impair its recognition by Keap1-Cul3 E3 ligase and promote malignancy. Proc. Natl. Acad. Sci. USA.

[67] Galan-Cobo A., Sitthideatphaiboon P., Qu X., Poteete A., Pisegna M.A., Tong P., Chen P.H., Boroughs L.K., Rodriguez M.L.M., Zhang W. (2019). LKB1 and KEAP1/NRF2 Pathways Cooperatively Promote Metabolic Reprogramming with Enhanced Glutamine Dependence in KRAS-Mutant Lung Adenocarcinoma. Cancer Res..

[68] Jeong Y., Hellyer J.A., Stehr H., Hoang N.T., Niu X., Das M., Padda S.K., Ramchandran K., Neal J.W., Wakelee H. (2020). Role of KEAP1/NFE2L2 Mutations in the Chemotherapeutic Response of Patients with Non-Small Cell Lung Cancer. Clin. Cancer Res..

[69] Cuadrado A., Kügler S., Lastres-Becker I. (2018). Pharmacological targeting of GSK-3 and NRF2 provides neuroprotection in a preclinical model of tauopathy. Redox Biol..

[70] Patibandla C., van Aalten L., Dinkova-Kostova A.T., Honda T., Cuadrado A., Fernández-Ginés R., McNeilly A.D., Hayes J.D., Cantley J., Sutherland C. (2024). Inhibition of glycogen synthase kinase-3 enhances NRF2 protein stability, nuclear localisation and target gene transcription in pancreatic beta cells. Redox Biol..

[71] Pi J., Zhang Q., Fu J., Woods C.G., Hou Y., Corkey B.E., Collins S., Andersen M.E. (2010). ROS signaling, oxidative stress and Nrf2 in pancreatic beta-cell function. Toxicol. Appl. Pharmacol..

[72] Lastres-Becker I., García-Yagüe A.J., Scannevin R.H., Casarejos M.J., Kügler S., Rábano A., Cuadrado A. (2016). Repurposing the NRF2 Activator Dimethyl Fumarate as Therapy Against Synucleinopathy in Parkinson’s Disease. Antioxid. Redox Signal..

[73] Rojo A.I., Medina-Campos O.N., Rada P., Zúñiga-Toalá A., López-Gazcón A., Espada S., Pedraza-Chaverri J., Cuadrado A. (2012). Signaling pathways activated by the phytochemical nordihydroguaiaretic acid contribute to a Keap1-independent regulation of Nrf2 stability: Role of glycogen synthase kinase-3. Free Radic. Biol. Med..

[74] Sandberg M., Patil J., D’Angelo B., Weber S.G., Mallard C. (2014). NRF2-regulation in brain health and disease: Implication of cerebral inflammation. Neuropharmacology.

[75] DeNicola G.M., Karreth F.A., Humpton T.J., Gopinathan A., Wei C., Frese K., Mangal D., Yu K.H., Yeo C.J., Calhoun E.S. (2011). Oncogene-induced Nrf2 transcription promotes ROS detoxification and tumorigenesis. Nature.

[76] Mitsuishi Y., Taguchi K., Kawatani Y., Shibata T., Nukiwa T., Aburatani H., Yamamoto M., Motohashi H. (2012). Nrf2 redirects glucose and glutamine into anabolic pathways in metabolic reprogramming. Cancer Cell.

[77] Hasegawa K., Miwa S., Tsutsumiuchi K., Miwa J. (2010). Allyl isothiocyanate that induces GST and UGT expression confers oxidative stress resistance on C. elegans, as demonstrated by nematode biosensor. PLoS ONE.

[78] Zaffagnini G., Savova A., Danieli A., Romanov J., Tremel S., Ebner M., Peterbauer T., Sztacho M., Trapannone R., Tarafder A.K. (2018). p62 filaments capture and present ubiquitinated cargos for autophagy. EMBO J..

[79] Kageyama S., Gudmundsson S.R., Sou Y.S., Ichimura Y., Tamura N., Kazuno S., Ueno T., Miura Y., Noshiro D., Abe M. (2021). p62/SQSTM1-droplet serves as a platform for autophagosome formation and anti-oxidative stress response. Nat. Commun..

[80] Yang Y., Willis T.L., Button R.W., Strang C.J., Fu Y., Wen X., Grayson P.R.C., Evans T., Sipthorpe R.J., Roberts S.L. (2019). Cytoplasmic DAXX drives SQSTM1/p62 phase condensation to activate Nrf2-mediated stress response. Nat. Commun..

[81] Kirkin V., Lamark T., Sou Y.S., Bjørkøy G., Nunn J.L., Bruun J.A., Shvets E., McEwan D.G., Clausen T.H., Wild P. (2009). A role for NBR1 in autophagosomal degradation of ubiquitinated substrates. Mol. Cell.

[82] Bao L., Festa F., Freet C.S., Lee J.P., Hirschler-Laszkiewicz I.M., Chen S.J., Keefer K.A., Wang H.G., Patterson A.D., Cheung J.Y. (2019). The Human Transient Receptor Potential Melastatin 2 Ion Channel Modulates ROS Through Nrf2. Sci. Rep..

[83] Cheung K.L., Lee J.H., Shu L., Kim J.H., Sacks D.B., Kong A.N. (2013). The Ras GTPase-activating-like protein IQGAP1 mediates Nrf2 protein activation via the mitogen-activated protein kinase/extracellular signal-regulated kinase (ERK) kinase (MEK)-ERK pathway. J. Biol. Chem..

[84] Wei T., Choi S., Buehler D., Anderson R.A., Lambert P.F. (2020). A PI3K/AKT Scaffolding Protein, IQ Motif-Containing GTPase Associating Protein 1 (IQGAP1), Promotes Head and Neck Carcinogenesis. Clin. Cancer Res..

[85] Chen C., Carrillo N.D., Chen M., Wen T., Awasthi P., Anderson R.A., Cryns V.L. (2025). Regulation of NRF2 by stably associated phosphoinositides and small heat shock proteins in response to stress. J. Biol. Chem..

[86] Yu E.Y., Ellard S.L., Hotte S.J., Gingerich J.R., Joshua A.M., Gleave M.E., Chi K.N. (2018). A randomized phase 2 study of a HSP27 targeting antisense, apatorsen with prednisone versus prednisone alone, in patients with metastatic castration resistant prostate cancer. Investig. New Drugs.

[87] Ozleyen A., Duran G.N., Donmez S., Ozbil M., Doveston R.G., Tumer T.B. (2025). Identification and inhibition of PIN1-NRF2 protein-protein interactions through computational and biophysical approaches. Sci. Rep..

[88] Saeidi S., Kim S.J., Guillen-Quispe Y.N., Jagadeesh A.S.V., Han H.J., Kim S.H., Zhong X., Piao J.Y., Kim S.J., Jeong J. (2022). Peptidyl-prolyl cis-trans isomerase NIMA-interacting 1 directly binds and stabilizes Nrf2 in breast cancer. FASEB J..

[89] Zhang Z., Hu Q., Ye S., Xiang L. (2022). Inhibition of the PIN1-NRF2/GPX4 axis imparts sensitivity to cisplatin in cervical cancer cells. Acta Biochim. Biophys. Sin..

[90] Lu W., Cui J., Wang W., Hu Q., Xue Y., Liu X., Gong T., Lu Y., Ma H., Yang X. (2024). PPIA dictates NRF2 stability to promote lung cancer progression. Nat. Commun..

[91] Katoh Y., Itoh K., Yoshida E., Miyagishi M., Fukamizu A., Yamamoto M. (2001). Two domains of Nrf2 cooperatively bind CBP, a CREB binding protein, and synergistically activate transcription. Genes Cells.

[92] Sun Z., Chin Y.E., Zhang D.D. (2009). Acetylation of Nrf2 by p300/CBP augments promoter-specific DNA binding of Nrf2 during the antioxidant response. Mol. Cell. Biol..

[93] Moore S., Berger N.D., Luijsterburg M.S., Piett C.G., Stanley F.K.T., Schräder C.U., Fang S., Chan J.A., Schriemer D.C., Nagel Z.D. (2019). The CHD6 chromatin remodeler is an oxidative DNA damage response factor. Nat. Commun..

[94] Nioi P., Nguyen T., Sherratt P.J., Pickett C.B. (2005). The carboxy-terminal Neh3 domain of Nrf2 is required for transcriptional activation. Mol. Cell. Biol..

[95] Itoh K., Chiba T., Takahashi S., Ishii T., Igarashi K., Katoh Y., Oyake T., Hayashi N., Satoh K., Hatayama I. (1997). An Nrf2/small Maf heterodimer mediates the induction of phase II detoxifying enzyme genes through antioxidant response elements. Biochem. Biophys. Res. Commun..

[96] Wang H., Liu K., Geng M., Gao P., Wu X., Hai Y., Li Y., Li Y., Luo L., Hayes J.D. (2013). RXRα inhibits the NRF2-ARE signaling pathway through a direct interaction with the Neh7 domain of NRF2. Cancer Res..

[97] Ki S.H., Cho I.J., Choi D.W., Kim S.G. (2005). Glucocorticoid receptor (GR)-associated SMRT binding to C/EBPbeta TAD and Nrf2 Neh4/5: Role of SMRT recruited to GR in GSTA2 gene repression. Mol. Cell. Biol..

[98] Karapetian R.N., Evstafieva A.G., Abaeva I.S., Chichkova N.V., Filonov G.S., Rubtsov Y.P., Sukhacheva E.A., Melnikov S.V., Schneider U., Wanker E.E. (2005). Nuclear oncoprotein prothymosin alpha is a partner of Keap1: Implications for expression of oxidative stress-protecting genes. Mol. Cell. Biol..

[99] Saito T., Ichimura Y., Taguchi K., Suzuki T., Mizushima T., Takagi K., Hirose Y., Nagahashi M., Iso T., Fukutomi T. (2016). p62/Sqstm1 promotes malignancy of HCV-positive hepatocellular carcinoma through Nrf2-dependent metabolic reprogramming. Nat. Commun..

[100] Roy M., Li Z., Sacks D.B. (2004). IQGAP1 binds ERK2 and modulates its activity. J. Biol. Chem..

[101] Hedman A.C., Smith J.M., Sacks D.B. (2015). The biology of IQGAP proteins: Beyond the cytoskeleton. EMBO Rep..

[102] Kim J.H., Xu E.Y., Sacks D.B., Lee J., Shu L., Xia B., Kong A.N. (2013). Identification and functional studies of a new Nrf2 partner IQGAP1: A critical role in the stability and transactivation of Nrf2. Antioxid. Redox Signal..

[103] Lu K.P., Zhou X.Z. (2007). The prolyl isomerase PIN1: A pivotal new twist in phosphorylation signalling and disease. Nat. Rev. Mol. Cell Biol..

[104] Driver J.A., Zhou X.Z., Lu K.P. (2015). Pin1 dysregulation helps to explain the inverse association between cancer and Alzheimer’s disease. Biochim. Biophys. Acta.

[105] Bao L., Kimzey A., Sauter G., Sowadski J.M., Lu K.P., Wang D.G. (2004). Prevalent overexpression of prolyl isomerase Pin1 in human cancers. Am. J. Pathol..

[106] Liang C., Shi S., Liu M., Qin Y., Meng Q., Hua J., Ji S., Zhang Y., Yang J., Xu J. (2019). PIN1 Maintains Redox Balance via the c-Myc/NRF2 Axis to Counteract Kras-Induced Mitochondrial Respiratory Injury in Pancreatic Cancer Cells. Cancer Res..

[107] Campaner E., Rustighi A., Zannini A., Cristiani A., Piazza S., Ciani Y., Kalid O., Golan G., Baloglu E., Shacham S. (2017). A covalent PIN1 inhibitor selectively targets cancer cells by a dual mechanism of action. Nat. Commun..

[108] Dubiella C., Pinch B.J., Koikawa K., Zaidman D., Poon E., Manz T.D., Nabet B., He S., Resnick E., Rogel A. (2021). Sulfopin is a covalent inhibitor of Pin1 that blocks Myc-driven tumors in vivo. Nat. Chem. Biol..

[109] Davis T.L., Walker J.R., Campagna-Slater V., Finerty P.J., Paramanathan R., Bernstein G., MacKenzie F., Tempel W., Ouyang H., Lee W.H. (2010). Structural and biochemical characterization of the human cyclophilin family of peptidyl-prolyl isomerases. PLoS Biol..

[110] Wang P., Heitman J. (2005). The cyclophilins. Genome Biol..

[111] Itoh K., Igarashi K., Hayashi N., Nishizawa M., Yamamoto M. (1995). Cloning and characterization of a novel erythroid cell-derived CNC family transcription factor heterodimerizing with the small Maf family proteins. Mol. Cell. Biol..

[112] Rushmore T.H., Morton M.R., Pickett C.B. (1991). The antioxidant responsive element. Activation by oxidative stress and identification of the DNA consensus sequence required for functional activity. J. Biol. Chem..

[113] Wasserman W.W., Fahl W.E. (1997). Functional antioxidant responsive elements. Proc. Natl. Acad. Sci. USA.

[114] Katsuoka F., Yamamoto M. (2016). Small Maf proteins (MafF, MafG, MafK): History, structure and function. Gene.

[115] Motohashi H., O’Connor T., Katsuoka F., Engel J.D., Yamamoto M. (2002). Integration and diversity of the regulatory network composed of Maf and CNC families of transcription factors. Gene.

[116] Oyake T., Itoh K., Motohashi H., Hayashi N., Hoshino H., Nishizawa M., Yamamoto M., Igarashi K. (1996). Bach proteins belong to a novel family of BTB-basic leucine zipper transcription factors that interact with MafK and regulate transcription through the NF-E2 site. Mol. Cell Biol..

[117] Suzuki H., Tashiro S., Hira S., Sun J., Yamazaki C., Zenke Y., Ikeda-Saito M., Yoshida M., Igarashi K. (2004). Heme regulates gene expression by triggering Crm1-dependent nuclear export of Bach1. EMBO J..

[118] Watanabe-Matsui M., Muto A., Matsui T., Itoh-Nakadai A., Nakajima O., Murayama K., Yamamoto M., Ikeda-Saito M., Igarashi K. (2011). Heme regulates B-cell differentiation, antibody class switch, and heme oxygenase-1 expression in B cells as a ligand of Bach2. Blood.

[119] Ortega E., Rengachari S., Ibrahim Z., Hoghoughi N., Gaucher J., Holehouse A.S., Khochbin S., Panne D. (2018). Transcription factor dimerization activates the p300 acetyltransferase. Nature.

[120] Kawai Y., Garduño L., Theodore M., Yang J., Arinze I.J. (2011). Acetylation-deacetylation of the transcription factor Nrf2 (nuclear factor erythroid 2-related factor 2) regulates its transcriptional activity and nucleocytoplasmic localization. J. Biol. Chem..

[121] Yu T., Ding C., Peng J., Liang G., Tang Y., Zhao J., Li Z. (2025). SIRT7-mediated NRF2 deacetylation promotes antioxidant response and protects against chemodrug-induced liver injury. Cell Death Dis..

[122] Lonard D.M., O’Malley B.W. (2006). The expanding cosmos of nuclear receptor coactivators. Cell.

[123] Yi P., Wang Z., Feng Q., Pintilie G.D., Foulds C.E., Lanz R.B., Ludtke S.J., Schmid M.F., Chiu W., O’Malley B.W. (2015). Structure of a biologically active estrogen receptor-coactivator complex on DNA. Mol. Cell.

[124] Bedford M.T., Clarke S.G. (2009). Protein arginine methylation in mammals: Who, what, and why. Mol. Cell.

[125] Allen B.L., Taatjes D.J. (2015). The Mediator complex: A central integrator of transcription. Nat. Rev. Mol. Cell Biol..

[126] Soutourina J. (2018). Transcription regulation by the Mediator complex. Nat. Rev. Mol. Cell Biol..

[127] Marfella C.G., Imbalzano A.N. (2007). The Chd family of chromatin remodelers. Mutat. Res..

[128] Lacher S.E., Lee J.S., Wang X., Campbell M.R., Bell D.A., Slattery M. (2015). Beyond antioxidant genes in the ancient Nrf2 regulatory network. Free Radic. Biol. Med..

[129] Romero R., Sayin V.I., Davidson S.M., Bauer M.R., Singh S.X., LeBoeuf S.E., Karakousi T.R., Ellis D.C., Bhutkar A., Sánchez-Rivera F.J. (2017). Keap1 loss promotes Kras-driven lung cancer and results in dependence on glutaminolysis. Nat. Med..

[130] Binkley M.S., Jeon Y.J., Nesselbush M., Moding E.J., Nabet B.Y., Almanza D., Kunder C., Stehr H., Yoo C.H., Rhee S. (2020). KEAP1/NFE2L2 Mutations Predict Lung Cancer Radiation Resistance That Can Be Targeted by Glutaminase Inhibition. Cancer Discov..

[131] Igarashi K., Nishizawa H., Saiki Y., Matsumoto M. (2021). The transcription factor BACH1 at the crossroads of cancer biology: From epithelial-mesenchymal transition to ferroptosis. J. Biol. Chem..

[132] Nishizawa H., Matsumoto M., Shindo T., Saigusa D., Kato H., Suzuki K., Sato M., Ishii Y., Shimokawa H., Igarashi K. (2020). Ferroptosis is controlled by the coordinated transcriptional regulation of glutathione and labile iron metabolism by the transcription factor BACH1. J. Biol. Chem..

[133] Nishizawa H., Yamanaka M., Igarashi K. (2023). Ferroptosis: Regulation by competition between NRF2 and BACH1 and propagation of the death signal. FEBS J..

[134] Friedmann Angeli J.P., Schneider M., Proneth B., Tyurina Y.Y., Tyurin V.A., Hammond V.J., Herbach N., Aichler M., Walch A., Eggenhofer E. (2014). Inactivation of the ferroptosis regulator Gpx4 triggers acute renal failure in mice. Nat. Cell Biol..

[135] Doll S., Freitas F.P., Shah R., Aldrovandi M., da Silva M.C., Ingold I., Goya Grocin A., Xavier da Silva T.N., Panzilius E., Scheel C.H. (2019). FSP1 is a glutathione-independent ferroptosis suppressor. Nature.

[136] Cuadrado A. (2022). Brain-Protective Mechanisms of Transcription Factor NRF2: Toward a Common Strategy for Neurodegenerative Diseases. Annu. Rev. Pharmacol. Toxicol..

[137] Suh J.H., Shenvi S.V., Dixon B.M., Liu H., Jaiswal A.K., Liu R.M., Hagen T.M. (2004). Decline in transcriptional activity of Nrf2 causes age-related loss of glutathione synthesis, which is reversible with lipoic acid. Proc. Natl. Acad. Sci. USA.

[138] Branca C., Ferreira E., Nguyen T.V., Doyle K., Caccamo A., Oddo S. (2017). Genetic reduction of Nrf2 exacerbates cognitive deficits in a mouse model of Alzheimer’s disease. Hum. Mol. Genet..

[139] Dinkova-Kostova A.T., Kostov R.V., Kazantsev A.G. (2018). The role of Nrf2 signaling in counteracting neurodegenerative diseases. FEBS J..

[140] Pajares M., Cuadrado A., Rojo A.I. (2017). Modulation of proteostasis by transcription factor NRF2 and impact in neurodegenerative diseases. Redox Biol..

[141] Yamazaki H., Tanji K., Wakabayashi K., Matsuura S., Itoh K. (2015). Role of the Keap1/Nrf2 pathway in neurodegenerative diseases. Pathol. Int..

[142] Salazar M., Rojo A.I., Velasco D., de Sagarra R.M., Cuadrado A. (2006). Glycogen synthase kinase-3beta inhibits the xenobiotic and antioxidant cell response by direct phosphorylation and nuclear exclusion of the transcription factor Nrf2. J. Biol. Chem..

[143] del Ser T., Steinwachs K.C., Gertz H.J., Andrés M.V., Gómez-Carrillo B., Medina M., Vericat J.A., Redondo P., Fleet D., León T. (2013). Treatment of Alzheimer’s disease with the GSK-3 inhibitor tideglusib: A pilot study. J. Alzheimers Dis..

[144] Di Martino R.M.C., Pruccoli L., Bisi A., Gobbi S., Rampa A., Martinez A., Pérez C., Martinez-Gonzalez L., Paglione M., Di Schiavi E. (2020). Novel Curcumin-Diethyl Fumarate Hybrid as a Dualistic GSK-3β Inhibitor/Nrf2 Inducer for the Treatment of Parkinson’s Disease. ACS Chem. Neurosci..

[145] Gold R., Kappos L., Arnold D.L., Bar-Or A., Giovannoni G., Selmaj K., Tornatore C., Sweetser M.T., Yang M., Sheikh S.I. (2012). Placebo-controlled phase 3 study of oral BG-12 for relapsing multiple sclerosis. N. Engl. J. Med..

[146] Linker R.A., Lee D.H., Ryan S., van Dam A.M., Conrad R., Bista P., Zeng W., Hronowsky X., Buko A., Chollate S. (2011). Fumaric acid esters exert neuroprotective effects in neuroinflammation via activation of the Nrf2 antioxidant pathway. Brain.

[147] Lynch D.R., Chin M.P., Delatycki M.B., Subramony S.H., Corti M., Hoyle J.C., Boesch S., Nachbauer W., Mariotti C., Mathews K.D. (2021). Safety and Efficacy of Omaveloxolone in Friedreich Ataxia (MOXIe Study). Ann. Neurol..

[148] Lynch D.R., Goldsberry A., Rummey C., Farmer J., Boesch S., Delatycki M.B., Giunti P., Hoyle J.C., Mariotti C., Mathews K.D. (2024). Propensity matched comparison of omaveloxolone treatment to Friedreich ataxia natural history data. Ann. Clin. Transl. Neurol..

[149] Cuadrado A. (2015). Structural and functional characterization of Nrf2 degradation by glycogen synthase kinase 3/β-TrCP. Free Radic. Biol. Med..

[150] Nezu M., Suzuki N. (2020). Roles of Nrf2 in Protecting the Kidney from Oxidative Damage. Int. J. Mol. Sci..

[151] Howden R. (2013). Nrf2 and cardiovascular defense. Oxid. Med. Cell. Longev..

[152] Cho H.Y., Kleeberger S.R. (2015). Association of Nrf2 with airway pathogenesis: Lessons learned from genetic mouse models. Arch. Toxicol..

[153] Holmström K.M., Kostov R.V., Dinkova-Kostova A.T. (2016). The multifaceted role of Nrf2 in mitochondrial function. Curr. Opin. Toxicol..

[154] Strom J., Xu B., Tian X., Chen Q.M. (2016). Nrf2 protects mitochondrial decay by oxidative stress. FASEB J..

[155] Hochmuth C.E., Biteau B., Bohmann D., Jasper H. (2011). Redox regulation by Keap1 and Nrf2 controls intestinal stem cell proliferation in Drosophila. Cell Stem Cell.

[156] Sykiotis G.P., Bohmann D. (2010). Stress-activated cap’n’collar transcription factors in aging and human disease. Sci. Signal..

[157] Türei D., Papp D., Fazekas D., Földvári-Nagy L., Módos D., Lenti K., Csermely P., Korcsmáros T. (2013). NRF2-ome: An integrated web resource to discover protein interaction and regulatory networks of NRF2. Oxid. Med. Cell. Longev..

[158] Hammad A., Namani A., Elshaer M., Wang X.J., Tang X. (2019). “NRF2 addiction” in lung cancer cells and its impact on cancer therapy. Cancer Lett..

[159] Crisman E., Duarte P., Dauden E., Cuadrado A., Rodríguez-Franco M.I., López M.G., León R. (2023). KEAP1-NRF2 protein-protein interaction inhibitors: Design, pharmacological properties and therapeutic potential. Med. Res. Rev..

[160] Hu L., Magesh S., Chen L., Wang L., Lewis T.A., Chen Y., Khodier C., Inoyama D., Beamer L.J., Emge T.J. (2013). Discovery of a small-molecule inhibitor and cellular probe of Keap1-Nrf2 protein-protein interaction. Bioorganic Med. Chem. Lett..

[161] Wang K., Baird L., Yamamoto M. (2025). The clinical-grade CBP/p300 inhibitor CCS1477 represses the global NRF2-dependent cytoprotective transcription program and re-sensitizes cancer cells to chemotherapeutic drugs. Free Radic. Biol. Med..

[162] Wang Y., Lonard D.M., Yu Y., Chow D.C., Palzkill T.G., Wang J., Qi R., Matzuk A.J., Song X., Madoux F. (2014). Bufalin is a potent small-molecule inhibitor of the steroid receptor coactivators SRC-3 and SRC-1. Cancer Res..

[163] Spigel D.R., Shipley D.L., Waterhouse D.M., Jones S.F., Ward P.J., Shih K.C., Hemphill B., McCleod M., Whorf R.C., Page R.D. (2019). A Randomized, Double-Blinded, Phase II Trial of Carboplatin and Pemetrexed with or without Apatorsen (OGX-427) in Patients with Previously Untreated Stage IV Non-Squamous-Non-Small-Cell Lung Cancer: The SPRUCE Trial. Oncologist.

[164] Song X., Zhang C., Zhao M., Chen H., Liu X., Chen J., Lonard D.M., Qin L., Xu J., Wang X. (2015). Steroid Receptor Coactivator-3 (SRC-3/AIB1) as a Novel Therapeutic Target in Triple Negative Breast Cancer and Its Inhibition with a Phospho-Bufalin Prodrug. PLoS ONE.

[165] Song X., Chen J., Zhao M., Zhang C., Yu Y., Lonard D.M., Chow D.C., Palzkill T., Xu J., O’Malley B.W. (2016). Development of potent small-molecule inhibitors to drug the undruggable steroid receptor coactivator-3. Proc. Natl. Acad. Sci. USA.

[166] Ahuja M., Ammal Kaidery N., Attucks O.C., McDade E., Hushpulian D.M., Gaisin A., Gaisina I., Ahn Y.H., Nikulin S., Poloznikov A. (2021). Bach1 derepression is neuroprotective in a mouse model of Parkinson’s disease. Proc. Natl. Acad. Sci. USA.

[167] Casares L., García V., Garrido-Rodríguez M., Millán E., Collado J.A., García-Martín A., Peñarando J., Calzado M.A., de la Vega L., Muñoz E. (2020). Cannabidiol induces antioxidant pathways in keratinocytes by targeting BACH1. Redox Biol..

