# Supplementary material for: Beyond KEAP1: The Context-Specific NRF2 Partner Code in Disease and Therapy
Source: Antioxidants (Basel). 2026 Jun 16;15(6):759. doi: 10.3390/antiox15060759 (PMC13295323; doi:10.3390/antiox15060759)
Supplement: Supplementary file 1 [file antioxidants-15-00759-s001.zip › antioxidants-4363549-supplementary.pdf]

Supplementary Materials

"Beyond KEAP1: The Context-Specific NRF2 Partner Code in Disease and Therapy"

Contents

S1. Glossary of partner-code framework terms
Table S1. The 22 partners included in the partner-code atlas
Table S2. Partner summary matrix
Table S3. Clinical trials targeting NRF2 partner-code interfaces
Table S4. The ~25 pharmacologically addressable interfaces
Table S5. NRF2 target gene programs by partner combination
Figure S1. Disease-genotype-cofactor stratification grid

S1. Glossary of partner-code framework terms

Term	Definition (as used in this review)
Partner code	A combinatorial regulatory system in which NRF2 transcriptional output is determined by which subset of partners is engaged across four partly independent modules (degradation, cytoplasmic scaffold, nuclear coactivator, and DNA/chromatin). The partner code is operational rather than metaphorical: the criteria for module assignment, partner inclusion, and evidence grading are specified in Box 1 and Supplementary Tables S1 and S3.
Module	A functional grouping of NRF2 partners that share a mechanism class (e.g., E3-ligase-mediated turnover, cytoplasmic scaffolding, nuclear coactivation, or DNA/chromatin engagement). Modules are partly independent (semi-orthogonal): partners within a module act on a shared mechanistic step, while different modules contribute distinguishable layers of regulation. Modules are not fully orthogonal — bridging partners exist (see below).
Bridging partner	A partner that satisfies the assignment criteria for two modules. Bridging partners are listed in their primary module per Box 1 and explicitly cross-referenced. Examples: p62/SQSTM1 (primary: degradation via KEAP1 sequestration; secondary: cytoplasmic scaffold via PB1 condensate formation); PIN1 (primary: cytoplasmic scaffold via prolyl isomerase activity; secondary: degradation via β-TrCP/Neh6 conformational priming).
Combinatorial assembly	The mechanism by which a finite NRF2 partner alphabet (~22 partners in the current atlas) generates diverse transcriptional outputs through different on-DNA combinations. The key prediction is that distinct partner combinations at the same NRF2 produce distinct target-gene programs (see Supplementary Table S5), rather than a single, uniform NRF2 output.
Rate-limiting partner	In a given cellular or disease context, the partner whose function (or pharmacological perturbation) most strongly determines NRF2 output. The identity of the rate-limiting partner is context-dependent: KEAP1 in physiological stress, β-TrCP/GSK-3β in metabolic disease, MED16 in coactivator-limited transcription. Identifying the rate-limiting partner per context is a central prediction the framework offers for biomarker-driven therapy.
Partner-selective therapy	A therapeutic strategy that targets a specific partner-NRF2 interface (e.g., PPIA-NRF2 by cyclosporin A, RAC3/SRC-3-NRF2 by SI-2/CCS1477, BACH1 by hemin) rather than NRF2 itself or KEAP1. The framework's central pharmacological hypothesis is that partner-selective intervention can deliver disease-specific NRF2 modulation with a more favorable therapeutic window than broad NRF2 pathway activation or inhibition.
Partner-stratified pharmacopeia	A disease-genotype-specific drug selection schema in which the partner-code module containing the rate-limiting partner determines the appropriate therapeutic class. The pharmacopeia is stratified into four tiers (Table 3 of the main text; Supplementary Table S2): Tier 1 approved NRF2-pathway therapeutics, Tier 2 approved drugs with repositioning hypotheses, Tier 3 clinical-stage partner-adjacent agents, Tier 4 preclinical or conceptual interfaces.
Disease rewiring	Context-specific perturbation of the partner code in which one module or one partner becomes dominantly perturbed in a disease state, while the other modules remain largely intact. Examples: KEAP1 loss-of-function in NSCLC rewires the degradation module while leaving scaffold, coactivator, and DNA modules intact; chronic β-TrCP overactivity in neurodegeneration rewires the degradation module via the Neh6 phosphodegron; BACH1 stabilization in lung cancer rewires the DNA/chromatin module.
Linker partner zone	The interdomain linker regions of NRF2 — outside the canonical Neh domains — that are emerging as partner-binding territory. Examples include the Neh2-Neh4 linker (Pro174 region engaged by PPIA) and the Neh6-Neh1 linker (Ser408 region engaged by PIN1). Hypothesis 1 of the main text predicts that systematic mapping of interdomain linkers will reveal additional partners with druggable interfaces.
Coactivator complex assembly	The combinatorial recruitment of acetyltransferases (CBP, p300), p160 coactivators (RAC3/SRC-3), arginine methyltransferases (PRMT1, CARM1), and Mediator subunits (MED16, MED23, MED24) at NRF2 Neh4/5. The framework's Hypothesis 3 predicts that NRF2 transcriptional amplitude in many contexts is rate-limited by coactivator assembly rather than by NRF2 protein stability, making coactivator-module interventions (e.g., CCS1477) a distinct therapeutic axis from KEAP1-pathway activation.

These ten terms are used consistently in the main text and across Supplementary Tables S1-S5. Downstream applications of the framework should adopt or explicitly redefine these terms; "partner code" is a neologism whose operational definition (see entry above and Box 1) anchors the framework's testability.

Table S1. The 22 partners included in the partner-code atlas

This supplementary table lists the 22 partners projected onto the Neh-domain coordinate atlas in Figure 3 and Section 5, together with their primary module assignment, inclusion rationale, and notes on boundary cases. Partners were included if (i) they had at least one mechanistically characterized direct or indirect interaction with NRF2 supported by biochemical, structural, or mutagenesis data, and (ii) they had been independently reproduced or had functional consequences for NRF2 output validated in at least one cellular or disease-model context. Excluded boundary partners are listed at the end of the table.

Counting rules for the 22-partner inventory. Three decisions explain how the count of 22 is reached. (i) Paralog families are counted once. Small Maf proteins (MafF, MafG, MafK) are counted as a single entry because they share the obligate-heterodimer binding mode at the Neh1 bZIP. β-TrCP1 and β-TrCP2 (FBXW11) are counted as a single entry for the SCF-Neh6 axis. CBP and p300 are listed separately because each has a distinct K588/K591 acetylation pattern on NRF2 with non-redundant transcriptional consequences. BACH1 and BACH2 are listed separately because they have tissue-restricted expression patterns and partly non-overlapping ARE/MARE target genes. (ii) Bridging partners are counted once. p62/SQSTM1 satisfies the operational criteria of both the degradation and the cytoplasmic scaffold modules; in this inventory, it is counted once and assigned to its primary module (degradation, via KEAP1 KIR motif competition), with the secondary scaffold role flagged in the Primary module column. PIN1 is similarly counted once with primary = cytoplasmic scaffold and secondary = degradation. This convention prevents partner count inflation and keeps the framework testable. (iii) Indirect partners are excluded if they do not bind NRF2 directly. KEAP1 sequestration partners that engage KEAP1 rather than NRF2 (DPP3, WTX/AMER1, NBR1, PALB2, p21/CDKN1A) are listed in the excluded-boundary section at the foot of the table; the same applies to upstream modulators (SIRT enzymes, KAP1/TRIM28, PTMA) that act on NRF2 indirectly without forming a stable binding interface. p62 is the only sequestration partner counted in the 22 because it satisfies an explicit module assignment via KEAP1 KIR + condensate role; the boundary cases lack a comparable bi-axial assignment.

Partner	Primary module	Inclusion rationale	Notes on evidence / boundary cases
KEAP1	Degradation	Direct E3 substrate adaptor; co-crystal with NRF2 ETGE; knockout stabilizes NRF2	Canonical anchor of the framework
β-TrCP (FBXW1A/FBXW11)	Degradation	Direct binder of GSK-3-primed Neh6 phosphodegron; SCF E3 ligase	Mutagenesis-validated (Rada/Cuadrado 2011)
Hrd1/SYVN1	Degradation	ER-resident E3 ligase ubiquitinating cytoplasmic NRF2 in liver cirrhosis	Co-IP + functional KO [14]
WDR23/DCAF11	Degradation	DDB1-CUL4 substrate adaptor binding Neh2 DIDLID motif; KEAP1-independent	Mutagenesis-validated [15]
p62 / SQSTM1	Degradation (bridging)	KIR motif (DPSTGE) competes with NRF2 ETGE; condensate-forming via PB1	Bridging partner; condensate function is cytoplasmic scaffold (Box 1)
IQGAP1	Cytoplasmic scaffold	Ca^{2+}/MEK-ERK relay; direct NRF2 binding shown in TRPM2-NRF2-IQGAP1 axis	Direct binding [78]
PIPKIγ–HSP27	Cytoplasmic scaffold	Phosphoinositide-coupled chaperone scaffold; recently described	Evidence maturity: low; remains to be independently validated
PIN1	Cytoplasmic scaffold (bridging)	Prolyl isomerase; conformational switching across pNRF2 at three Neh	Bridging partner; affects KEAP1 access via conformational change

Partner	Primary module	Inclusion rationale	Notes on evidence / boundary cases
		domains	
PPIA / cyclophilin A	Cytoplasmic scaffold	Direct NRF2 binding obstructs KEAP1 access; drugged by cyclosporin A	Direct binding [86]
CBP	Nuclear coactivator	Acetyltransferase binding Neh4/5; required for canonical NRF2 output	Anchor of the coactivator complex
p300	Nuclear coactivator	CBP paralog; distinct K588/K591 acetylation specificity	Direct binding; partial redundancy with CBP
RAC3 / SRC-3 / NCOA3	Nuclear coactivator	p160 coactivator; binds Neh4/5; CCS1477-targetable	Direct binding; clinical-stage drug (CCS1477)
PRMT1	Nuclear coactivator	Arginine methyltransferase; GSK3368715-targetable	Direct activity; clinical-stage inhibitor
CARM1	Nuclear coactivator	Methylates H3R17 at NRF2-bound enhancers; modulates amplitude	Indirect but consistently associated with NRF2 enhancers
MED16	Nuclear coactivator	Mediator tail subunit binding Neh4/5 + Neh1; disruption abolishes ~75% of NRF2 targets	Direct binding [16]; rate-limiting node, druggability low
MED23	Nuclear coactivator	Mediator subunit in NRF2 enhancer-to-promoter looping	Inferred; evidence maturity moderate
MED24	Nuclear coactivator	Mediator subunit; reported NRF2 cofactor in metabolic gene programs	Inferred; evidence maturity lower than MED16
Small Maf (MafF/G/K)	DNA/chromatin	Obligate heterodimer partner at Neh1 bZIP; defines ARE binding	Required for DNA binding; foundational
BACH1	DNA/chromatin	Competitive repressor at ARE/MARE; ratio with NRF2 sets ferroptosis outcome	Heme-sensing; lung cancer metastasis
BACH2	DNA/chromatin	BACH1 paralog; tissue-specific ARE competitor	Direct DNA-binding competitor
CHD6	DNA/chromatin	Chromatin remodeler at NRF2-bound enhancers	Direct co-occupancy; evidence maturity moderate
RXRα	DNA/chromatin	Nuclear receptor crosstalk binding NRF2 at Neh7; modulates output	Direct binding [92]

Excluded boundary partners: DPP3, WTX/AMER1, NBR1, PALB2, p21/CDKN1A — KEAP1 sequestration partners listed in Box 1 but excluded from the 22-partner atlas because they do not bind NRF2 directly. SIRT1/SIRT6/SIRT7 — modulate NRF2 PTMs but operate as enzymes rather than module partners. KAP1/TRIM28, PTMA — broader chromatin cofactors with non-NRF2-specific roles. GR (glucocorticoid receptor) — peripheral crosstalk; not consistently NRF2-dependent in published datasets.

Table S2. Partner summary matrix

This supplementary matrix tabulates each of the 22 partners in Table S1 along eight dimensions: primary module, secondary module (if bridging), whether direct NRF2 binding is established, whether the partner acts KEAP1-dependently or KEAP1-independently, the highest-quality evidence type supporting partner status, the corresponding therapeutic tier (1 approved NRF2-pathway therapeutic; 2 approved drug with repositioning hypothesis; 3 clinical-stage partner-adjacent agent; 4 preclinical/conceptual), and the disease-context directionality of partner modulation. Evidence type abbreviations: Struc = co-crystal structure; Mut = site-directed mutagenesis; coIP = co-immunoprecipitation; Inter = interactome screen; Funct = functional KO/KD with phenotype.

Partner	Primary	Secondary	Direct NRF2?	KEAP1-dep / indep	Evidence	Tier	Directionality
KEAP1	Deg	—	Yes	—	Struc + Mut	1	Inhibition desirable in most disease contexts; activation can be oncogenic in KEAP1-mutant cancer
β-TrCP	Deg	—	Yes	KEAP1-indep	Mut + Funct	2	Inhibition desirable in neurodegeneration and metabolic disease via GSK-3β axis
Hrd1/SYVN1	Deg	—	Yes	KEAP1-indep	coIP + Funct	4	Inhibition desirable in chronic liver disease
WDR23/DCAF11	Deg	—	Yes	KEAP1-indep	Mut + Funct	4	Context-dependent; nuclear NRF2 turnover
p62 / SQSTM1	Deg	Scaff	No (binds KEAP1)	KEAP1-dep	Struc + Funct	4	Context-dependent — protective in ferroptosis, oncogenic in HCC
IQGAP1	Scaff	—	Yes	KEAP1-indep	coIP + Funct	4	Inhibition may be desirable in KEAP1-mutant cancer
PIPKIγ–HSP27	Scaff	—	Inferred	Mixed	coIP	3	Inhibition tested in HSP27-driven cancer (apatorsen)
PIN1	Scaff	Deg	Yes	Mixed	coIP + Funct	2	Inhibition desirable in breast cancer (ATRA, ATO)
PPIA / CypA	Scaff	—	Yes	KEAP1-indep	coIP + Funct	2	Inhibition desirable in KEAP1-mutant NSCLC (cyclosporin A hypothesis)
CBP	Coact	—	Yes	—	coIP + Funct	3	Inhibition desirable in KEAP1-mutant cancer (CCS1477)
p300	Coact	—	Yes	—	coIP + Funct	3	Inhibition desirable in KEAP1-mutant cancer (CCS1477)
RAC3/SRC-3	Coact	—	Yes	—	coIP + Funct	4	Inhibition tested in TNBC, prostate cancer (SI-2, bufalin)
PRMT1	Coact	—	Yes (enzyme)	—	Funct	3	Inhibition tested in lymphoid malignancies (GSK3368715)
CARM1	Coact	—	Indirect	—	Inter + Funct	4	Modulates transcriptional amplitude
MED16	Coact	—	Yes	—	Mut + Funct	4	Conceptual only; ~75% of NRF2 targets depend on MED16

Partner	Primary	Secondary	Direct NRF2?	KEAP1-dep / indep	Evidence	Tier	Directionality
MED23	Coact	—	Indirect	—	Inter	4	Conceptual; enhancer-promoter looping
MED24	Coact	—	Indirect	—	Inter	4	Conceptual; metabolic gene programs
Small Maf	DNA	—	Yes (obligate)	—	Struc	4	Activation desirable but not directly drugged
BACH1	DNA	—	DNA-binding competitor	—	Struc + Funct	3	Inhibition desirable in KEAP1-mutant lung cancer; activation desirable in ferroptosis-prone tissue
BACH2	DNA	—	DNA-binding competitor	—	Mut + Funct	4	Tissue-specific competitor
CHD6	DNA	—	Indirect	—	Inter + Funct	4	Activation may enhance NRF2 output (conceptual)
RXRα	DNA	—	Yes	KEAP1-indep	coIP + Funct	2	Modulation via bexarotene repositioning hypothesis

Tier definitions: Tier 1 = FDA-approved NRF2-pathway therapeutic; Tier 2 = FDA-approved drug for a different indication with mechanistic basis for NRF2-related repositioning hypothesis; Tier 3 = clinical-stage partner-adjacent agent; Tier 4 = preclinical or conceptual.

Table S3. Clinical trials targeting NRF2 partner-code interfaces

This supplementary table catalogs registered clinical trials (ClinicalTrials.gov) that evaluate compounds targeting interfaces in the NRF2 partner code. Trials are included if (i) the primary or secondary mechanism plausibly engages an NRF2 partner-code module (degradation, cytoplasmic scaffold, nuclear coactivator, DNA/chromatin, or a validated downstream metabolic dependency), and (ii) the trial reached registration with a defined intervention and indication. Mechanism interpretation under the partner-code framework is provided in the rightmost column. Trial information was retrieved from ClinicalTrials.gov as of January 2026; status may have changed since this date and should be re-verified at the trial registry before any clinical decision.

NCT number	Compound	Module / target interface	Indication	Phase	Status (Jan 2026)	Partner-code interpretation
NCT02255435	Omaveloxolone (MOXIe)	Deg / KEAP1 Cys151	Friedreich ataxia	Phase II	Completed; FDA approved 2023	Tier 1 — covalent KEAP1 activator; reference for FA-stage neurodegeneration
NCT00420212 (DEFINE)	Dimethyl fumarate	Deg / KEAP1 Cys151	Multiple sclerosis	Phase III	Completed; FDA approved 2013	Tier 1 — paired with CONFIRM Phase III as approval basis
NCT00451451 (CONFIRM)	Dimethyl fumarate	Deg / KEAP1 Cys151	Multiple sclerosis	Phase III	Completed	Tier 1 — confirmatory Phase III for DMF MS indication
NCT01777763 (BEACON)	Bardoxolone methyl	Deg / KEAP1 Cys151	Type 2 diabetic CKD	Phase III	Terminated (cardiovascular safety)	Cautionary case — broad NRF2 activation safety liability
NCT03918447 (FALCON)	Bardoxolone methyl	Deg / KEAP1 Cys151	ADPKD	Phase III	Terminated (program discontinuation)	Reinforces tissue-specific safety constraints
NCT03068130 (RANGER)	Bardoxolone methyl	Deg / KEAP1 Cys151	Pulmonary arterial hypertension	Phase III	Terminated	Same compound, same liability — supports tissue-context framework
NCT04265534 (KEAPSAKE)	Telaglenastat (CB-839) + pembrolizumab/chemo	Downstream metabolic dependency (SLC1A5)	KEAP1-mutant NSCLC	Phase II	Terminated	Glutamine dependency in KEAP1-mut NSCLC; outcome consistent with downstream-only inhibition being insufficient
NCT03872427 (BeGIN)	Telaglenastat	Downstream metabolic dependency (SLC1A5)	KEAP1/NFE2L2-mut NSCLC	Phase II	Completed	Genotype-stratified entry; framework predicts biomarker stratification improves signal
NCT02417701	Sapanisertib (TAK-228)	Downstream (mTORC1/2)	KEAP1/NFE2L2-mut NSCLC	Phase II	Completed	Genotype-stratified mTOR targeting; informs combination logic
NCT04250545	Sapanisertib	Downstream (mTORC1/2)	KEAP1-mut squamous NSCLC	Phase II	Active	Squamous-specific KEAP1-mut indication
NCT01049399	Tideglusib (NP031112)	Deg / β-TrCP–Neh6 (via GSK-3β)	Progressive supranuclear palsy	Phase II	Completed (negative)	GSK-3β inhibition in tauopathy; negative outcome shapes biomarker-stratified redesign hypothesis

NCT number	Compound	Module / target interface	Indication	Phase	Status (Jan 2026)	Partner-code interpretation
NCT02586935	Tideglusib	Deg / β-TrCP–Neh6	Autism spectrum disorder (adolescents)	Phase II	Completed	Off-label GSK-3β indication; informs β-TrCP–NRF2 axis exposure-response
NCT00867282	Lithium carbonate	Deg / β-TrCP–Neh6 (via GSK-3β)	Amyotrophic lateral sclerosis	Phase II/III	Completed (negative on survival)	Unstratified ALS lithium trial; framework predicts biomarker stratification needed
NCT00874770	Lithium	Deg / β-TrCP–Neh6	Huntington disease	Phase II	Completed	GSK-3β inhibition in polyQ-driven neurodegeneration
Multiple (historical)	All-trans retinoic acid (ATRA)	Scaff / PIN1 active site	Acute promyelocytic leukemia	FDA approved	Standard of care	PIN1 inhibition proof-of-concept; partner-code framework reads as scaffold-module precedent
Multiple (historical)	Arsenic trioxide (ATO)	Scaff / PIN1 active site	Acute promyelocytic leukemia	FDA approved	Standard of care	Validated in PML-RARA fusion APL; partner-code analog for non-APL contexts
NCT01120470	Apatorsen (OGX-427)	Scaff / HSP27 (PIPKIγ–HSP27 axis)	Castration-resistant prostate cancer	Phase II	Completed (no signal)	HSP27 antisense in CRPC; framework reads negative outcome as scaffold-engagement uncertainty
NCT01829113	Apatorsen	Scaff / HSP27	NSCLC (RAINIER)	Phase II	Completed (negative)	Reinforces HSP27 evidence maturity caveat
NCT03568656	CCS1477 (inobrodib)	Coact / CBP-p300 BRD	Hematologic malignancies	Phase I/II	Active	Coactivator-module inhibitor; framework prediction for KEAP1-mut cancer combination
NCT03568656 (extension)	CCS1477	Coact / CBP-p300 BRD	Solid tumors (including CRPC)	Phase I/II	Active	Coactivator-module logic applicable across solid tumors
NCT03666988	GSK3368715	Coact / PRMT1	Hematologic / solid tumors	Phase I	Terminated	First-in-class PRMT1 inhibitor; informs coactivator-module druggability
Multiple (historical)	Hemin (Panhematin)	DNA-chrom / BACH1	Acute intermittent porphyria	FDA approved	Standard of care	BACH1 destabilization via heme; partner-code precedent for ferroptosis-protective indication
Multiple (RA, IBD)	Sulfasalazine	Downstream / SLC7A11 (xCT)	Rheumatoid arthritis, IBD	FDA approved	Standard of care	xCT inhibition; framework-implicated ferroptosis sensitizer in NRF2-high cancer
Multiple (CTCL)	Bexarotene	DNA-chrom / RXRα (Neh7 crosstalk)	Cutaneous T-cell lymphoma	FDA approved	Standard of care	RXRα agonism; partner-code repositioning hypothesis (Tier 2)
(no NRF2 trial)	Cyclosporin A	Scaff / PPIA–NRF2	KEAP1-mut NSCLC (hypothesis)	Repositioning hypothesis	Not yet trialed	Framework prediction (Section 7.3); biomarker-stratified investigator-initiated trial proposed

NCT number	Compound	Module / target interface	Indication	Phase	Status (Jan 2026)	Partner-code interpretation
Preclinical	KI-696	Deg / KEAP1 Kelch pocket	(various preclinical)	Preclinical	No clinical trial	First non-covalent KEAP1-PPI inhibitor; reference compound for non-electrophilic strategy
Various (cyclophilin)	NIM-811	Scaff / PPIA active site (non-immunosuppressive cyclosporin)	HCV (historical), repositioning hypothesis	Phase II (HCV, completed)	Discontinued for HCV	Non-immunosuppressive cyclophilin inhibitor; relevant for PPIA-NRF2 repositioning without calcineurin liability
Preclinical	Sulfopin	Scaff / PIN1 active site (covalent)	MYC-driven cancer (preclinical)	Preclinical	No clinical trial	Selective covalent PIN1 inhibitor; reference for PIN1-module druggability
Preclinical	SI-2	Coact / SRC-3 (RAC3)	TNBC, prostate cancer (preclinical)	Preclinical	No clinical trial	Coactivator-module tool compound; NRF2-specific engagement not yet validated

Stage color key: green = FDA-approved (for the listed indication); amber = clinically tested/active; gray = terminated, completed-negative, or preclinical; blue = repositioning hypothesis (not yet trialed). NCT numbers shown are representative; many compounds have multiple registered trials across indications. Status is from ClinicalTrials.gov as of January 2026 and should be re-verified at the registry.

Table S4. The ~25 pharmacologically addressable interfaces

This supplementary table enumerates the pharmacologically addressable interfaces identified across the four NRF2 modules. The list defines what is counted in the abstract claim of "approximately 25 pharmacologically addressable interfaces." Interfaces are stratified by developmental stage (FDA-approved indication; clinical / phase II–III; preclinical; conceptual only). Drugs in the FDA-approved column are approved for the indication shown but not all are approved for an NRF2-pathway indication; repositioning hypotheses are explicitly noted in Table 3 of the main text.

#	Interface	Pharmacological approach	Representative compound(s)	Stage
1	KEAP1 Kelch pocket	Small-molecule ETGE mimetics	KI-696, RA839	Preclinical
2	KEAP1 Cys151	Covalent electrophiles	Omaveloxolone, bardoxolone, DMF	FDA approved (omaveloxolone, DMF); bardoxolone programs terminated
3	KEAP1 Cys273/Cys288	Distinct electrophile chemistries	Various Michael acceptors	Preclinical
4	NRF2 KIR / ETGE	Peptide mimetic / PROTAC	Conceptual lead	Conceptual
5	β-TrCP–Neh6	GSK-3β inhibitors	Lithium, tideglusib, CHIR-99021	FDA approved (lithium); Phase II (tideglusib)
6	Hrd1/SYVN1 ERAD	Direct E3 inhibition	LS-102	Preclinical
7	WDR23 DIDLID	Substrate-adaptor disruptor	No lead	Conceptual
8	PPIA–NRF2	PPI disruption	Cyclosporin A (PPIA inhibitor)	FDA approved (for transplant); Tier 2 repositioning
9	PIN1 active site	Active-site inhibitor or covalent modifier	Sulfopin, KPT-6566, ATRA, ATO	FDA approved (ATRA, ATO for APL); Preclinical (sulfopin)
10	HSP27	Antisense oligonucleotide	Apatorsen / OGX-427	Phase II (terminated)
11	IQGAP1 IQ domain	Peptidomimetic	No lead	Conceptual
12	p62 KIR motif	Small-molecule disruptor	No lead	Preclinical
13	p62 PB1 (condensates)	Anti-condensate strategy	Tool compounds	Preclinical
14	PIPKIγ kinase domain	Kinase inhibitor (selectivity TBD)	No selective lead	Conceptual
15	CBP/p300 BRD	BRD inhibitor	CCS1477 / inobrodib	Phase I/II
16	CBP/p300 HAT	Catalytic HAT inhibitor	A-485, ML334	Preclinical
17	RAC3/SRC-3	Coactivator inhibitor	SI-2, bufalin	Preclinical
18	PRMT1	Methyltransferase inhibitor	GSK3368715	Phase I (terminated)
19	MED16 / Mediator tail	Disruption / PROTAC	No lead	Conceptual only
20	BACH1	Heme-mimetic / iron-loading	HPPE, hemin	Preclinical (HPPE); FDA approved (hemin for porphyria)
21	CHD6	Chromatin remodeler	No lead	Conceptual

#	Interface	Pharmacological approach	Representative compound(s)	Stage
22	RXRα (Neh7 crosstalk)	RXR agonist	Bexarotene	FDA approved (for CTCL); Tier 2 repositioning
23	SLC7A11/xCT (downstream)	System Xc⁻ inhibitor	Sulfasalazine	FDA approved (RA, IBD); Tier 2 repositioning
24	SLC1A5 / ASCT2 (downstream)	Glutaminase inhibitor (metabolic dependency)	Telaglenastat (CB-839)	Phase II (KEAPSAKE, terminated)
25	GPX4 (downstream of NRF2:BACH1)	Ferroptosis-related	RSL3, ML162 (research tools)	Preclinical

Stage color key: green = FDA-approved (for an NRF2-pathway indication); amber = clinically tested (approved for other indication OR clinical-stage); gray = preclinical; blue = conceptual only.

Table S5. NRF2 target gene programs by partner combination

This supplementary table operationalizes a central prediction of the partner-code framework: that distinct combinations of partners assemble at NRF2 produce distinct target-gene programs, rather than a single "NRF2 transcriptional output." Fifteen representative programs are listed below, each defined by (i) the partner combination predicted to assemble at the relevant ARE/MARE elements, (ii) the resulting gene program category, (iii) representative target genes, (iv) the cellular or disease context in which the program is dominant, (v) the confidence level supported by current evidence (A = directly validated by ChIP-seq plus genetic perturbation; B = supported by complementary biochemical and functional evidence; C = inferred from individual studies or framework reasoning).

#	Partner combination	Gene program category	Representative target genes	Cellular / disease context	Confidence
1	NRF2 + small Maf + CBP/p300 + RAC3 + MED16	Cytoprotective core	NQO1, GCLC, GCLM, HMOX1, GPX2, TXN1	Physiological stress response (most cell types)	A
2	NRF2 + small Maf + (low BACH1) + p300	Iron / ferroptosis-protective	FTH1, FTL, SLC7A11, GPX4, FSP1, AKR1B	Ferroptosis-prone tissue (renal tubule, hepatocyte, neuron)	A
3	NRF2 + (high BACH1) + small Maf	Pro-metastatic (NRF2 paradox)	Repression of ferroptosis genes + EMT/glycolysis activation (HK2, PDK1)	KEAP1-mutant lung cancer (metastasis)	A
4	NRF2 + p300 + (TBK1-phospho-p62) — KEAP1-loss context	Glutamine import / metabolic reprogramming	SLC1A5, KLF5, GLS, ASNS	KEAP1-mutant NSCLC (oncogenic metabolism)	A
5	NRF2 + small Maf + p300/CBP	NADPH / pentose phosphate	G6PD, PGD, TKT, IDH1	Proliferation, redox buffering	B
6	NRF2 + small Maf + p300/CBP	Phase II drug metabolism	GSTA1, GSTM1, GSTP1, UGT1A, NQO1	Hepatocyte xenobiotic response	A
7	NRF2 + small Maf	Phase III drug efflux	ABCC1 (MRP1), ABCC2, ABCG2 (BCRP)	Chemoresistance in KEAP1-mut cancer; pharmacokinetic barrier	B
8	NRF2 + small Maf + (BACH1 displaced by heme)	Heme detoxification (BACH1 switch)	HMOX1 (induction-specific)	Erythroid stress, hemolysis, inflammation	A
9	NRF2 + p300 + MED16	Proteostasis / autophagy	SQSTM1 (p62), PSMA, PSMB, ATG7	Proteostasis stress, neurodegeneration	B
10	NRF2 + (NRF1) + PGC-1α (cross-talk)	Mitochondrial biogenesis	TFAM, NRF1 (gene), mtDNA replication factors	Skeletal muscle, brown adipocyte, hepatocyte under metabolic stress	C
11	NRF2 + p300 + (BRCA1–PALB2 axis)	DNA damage response	RAD51, FANCD2, BRCA1 target genes	Genotoxic stress (cancer therapy response)	C
12	NRF2 + p300 + RXRα (Neh7 crosstalk)	Lipid metabolism / anti-fibrotic	FABP4, PPARα-related, anti-fibrotic genes	Hepatic stellate cell, lipid-loaded hepatocyte	C
13	NRF2 + p300 + (NF-κB p65 crosstalk)	Inflammation suppression	Repression of IL6, MMP9; induction of TNFAIP3	Activated macrophages, hepatic inflammation	B
14	NRF2 + RAC3 (in NCOA3-amplified	Stem cell / proliferation	MYC targets, proliferation genes	Stem-like cancer cells, NCOA3-amplified	C

#	Partner combination	Gene program category	Representative target genes	Cellular / disease context	Confidence
	context)			breast/prostate	
15	NRF2 + p300 + RAC3 + MED16 + PIN1 (KEAP1-mut)	Oncogenic / stress survival reprogramming	Mixed: SLC1A5, KLF5, drug-efflux ABCs, survival genes	KEAP1-mut cancer under therapy	B

Confidence color key: green (A) = directly validated by ChIP-seq plus genetic perturbation; yellow (B) = supported by complementary biochemical and functional evidence; orange (C) = inferred from individual studies or framework reasoning. Several programs share overlapping target genes (e.g., NQO1 appears in programs #1 and #6) because the partner combinations differ in context rather than in promoter occupancy. The framework predicts that compounds disrupting one program may have minimal effect on another, providing the mechanistic rationale for partner-selective rather than NRF2-pathway-wide pharmacology.

Figure S1. Disease-genotype-cofactor stratification grid

Disease-genotype context	KEAP1	β-TrCP	PPIA	PIN1	HSP27	CBP/p300	BACH1	Downstream	Conceptual
1. KEAP1-mut + NCOA3-amp NSCLC (Tier I)	— *KEAP1 axis lost, not applicable*	— *Not the rate-limiting axis*	**Cyclosporin A** *PPIA-NRF2 disruption hypothesis*	**ATRA / ATO** *PIN1-scaffold module dependence (analogical)*	— *Not the rate-limiting axis*	**CCS1477** *Coactivator hyperactivation via amplified NCOA3*	— *BACH1 not dominant in this subtype*	**Telaglenastat** *SLC1A5 dependency (KEAPSAKE Ph II)*	**MED16 disruptor** *MED16-dependent program (~75% of NRF2 targets)*
2. KEAP1-mut + PIN1-high NSCLC (Tier I)	— *KEAP1 axis lost*	— *Not dominant*	**Cyclosporin A** *Alternative scaffold disruption*	**Sulfopin** *Selective covalent PIN1 inhibitor (preclinical)*	— *Not dominant*	**CCS1477** *Coactivator-module combination*	— *Not dominant*	**Telaglenastat** *Metabolic dependency*	— —
3. KEAP1-mut + p62-aggregated NSCLC (Tier I)	— *KEAP1 sequestered by p62 condensates*	**BX795 (TBK1i)** *Disrupts TBK1-driven p62-condensate*	**Cyclosporin A** *Alternative scaffold disruption*	**ATRA** *PIN1-scaffold module*	— —	**CCS1477** *Coactivator combination*	— —	**Telaglenastat** *Metabolic dependency*	**p62-KIR disruptor** *Direct p62-KEAP1 PPI disruption*
4. KEAP1-WT + p62-aggregated HCC (Tier I)	**KI-696** *Restore KEAP1 access via Kelch PPI inhibitor*	— —	— —	— —	— —	— —	— —	**Sulfasalazine** *Ferroptosis sensitization (NRF2-high HCC)*	**p62-KIR disruptor** *Restore KEAP1 function*
5. KEAP1-WT + Hrd1-induced cirrhosis (Tier II)	— *KEAP1 intact; not rate-limiting*	— —	— —	— —	— —	— —	— —	— —	**LS-102** *Hrd1 inhibitor; IRE1α-XBP1 axis modulation*
6. KEAP1-WT + GSK-3β-hyperactive AD (Tier II)	— *Not rate-limiting*	**Lithium** *GSK-3β inhibition; biomarker-stratified pTau*	— —	— —	— —	— —	— —	— —	**Tideglusib** *Phase II GSK-3β; biomarker-stratified subset*
7. KEAP1-WT + GSK-3β-hyperactive PD / ALS (Tier II)	**Omaveloxolone** *Approved for FA; analogical for neurodegeneration*	**Lithium** *GSK-3β inhibition (unstratified trials negative; stratification proposed)*	— —	— —	— —	— —	— —	— —	**Tideglusib** *Phase II GSK-3β*
8. KEAP1-WT + AKT-driven T2D (Tier III)	**Bardoxolone (terminated)** *Phase III terminated for safety; cautionary*	**Lithium** *GSK-3β axis; exploratory for T2D*	— —	— —	— —	— —	— —	— —	— —
9. KEAP1-WT + heme-depleted ferroptosis-prone tissue (Tier I)	**DMF / Omaveloxolone** *Broad NRF2 activation (caution on safety)*	— —	— —	— —	— —	— —	**Hemin** *Approved for porphyria; destabilizes BACH1*	— —	**HPPE** *BACH1 destabilizer (preclinical)*
10. Friedreich ataxia (frataxin-deficient) (Tier I)	**Omaveloxolone (SKYCLARYS)** *FDA approved 2023 for FA*	— —	— —	— —	— —	— —	— —	— —	— —

Therapeutic readiness

FDA-approved | Clinical-stage | Preclinical only | Conceptual interface | No framework prediction

This grid is a framework-derived lookup tool, not a clinical-decision algorithm. All Tier 2 repositioning suggestions require partner-engagement validation before clinical use. Safety liabilities (e.g., immunosuppression for cyclosporin A; cardiovascular for bardoxolone; narrow therapeutic window for lithium) are not encoded here and must be considered separately.

Figure S1 legend

Rows represent ten disease-genotype-cofactor combinations spanning the strongly-supported, mechanistically-plausible, and exploratory tiers of partner-code rewiring (see Section 6 of the main text). Columns represent the partner-code-implicated therapy classes (KEAP1 modulators, β-TrCP/GSK-3β axis modulators, PPIA inhibitors, PIN1 inhibitors, HSP27 antisense, CBP/p300 inhibitors, BACH1 destabilizers, downstream metabolic inhibitors, and conceptual interfaces). Cell color encodes therapeutic readiness: green = FDA-approved (for the indication shown or another indication with mechanistic basis for partner-code repositioning); amber = clinical-stage; gray = preclinical only; blue = conceptual (no chemical lead). Cell label gives the representative compound name. Cell size or text weight may be used to encode evidence strength when rendered (small/regular = single study or analogical reasoning; large/bold = multiple independent studies or completed clinical trial).
